# A comprehensive ruminant microbial catalog (CRMC) reveals convergent selection for key vitamin-synthesizing pathways and genes across ruminants and human

**DOI:** 10.1093/gigascience/giag016

**Published:** 2026-02-25

**Authors:** Tong Feng, Yingjian Wu, Yixue Xu, Wei-Hua Chen

**Affiliations:** Key Laboratory of Molecular Biophysics of the Ministry of Education, Hubei Key Laboratory of Bioinformatics and Molecular Imaging, Center for Artificial Biology, Department of Bioinformatics and Systems Biology, College of Life Science and Technology, Huazhong University of Science and Technology, Wuhan 430074, Hubei, China; Key Laboratory of Molecular Biophysics of the Ministry of Education, Hubei Key Laboratory of Bioinformatics and Molecular Imaging, Center for Artificial Biology, Department of Bioinformatics and Systems Biology, College of Life Science and Technology, Huazhong University of Science and Technology, Wuhan 430074, Hubei, China; State Key Laboratory for Conservation and Utilization of Subtropical Agro-Bioresources, Guangxi University, Nanning 530005, China; Key Laboratory of Molecular Biophysics of the Ministry of Education, Hubei Key Laboratory of Bioinformatics and Molecular Imaging, Center for Artificial Biology, Department of Bioinformatics and Systems Biology, College of Life Science and Technology, Huazhong University of Science and Technology, Wuhan 430074, Hubei, China; School of Biological Science, Jining Medical University, Rizhao 272111, China

**Keywords:** ruminant, gastrointestinal tract, microbiome, metagenome-assembled genomes, vitamin synthesizing, cross-species comparison

## Abstract

**Background:**

The ruminant gastrointestinal tract (GIT) serves as a natural microbial reservoir in which vitamin-synthesizing microbes play key integrated roles in digestion, nutrient absorption, and metabolic balance; however, studies systematically elucidating their functional characteristics and ecological roles remain limited due to the lack of a large-scale reference genome catalog for ruminant gastrointestinal vitamin-synthesizing microbes. Here, based on 2,325 metagenomic samples from 8 ruminant hosts, we comprehensively reconstructed and analyzed the ruminant GIT microbiome and the distribution patterns of vitamin-synthesizing microbes.

**Results:**

We reconstructed a unified ruminant gastrointestinal microbiome catalog (CRMC) with 39,696 MAGs, achieving the highest mapping rate (~83.45%) among 2,325 metagenomic datasets, surpassing GTDB, RGMGC, and other catalogs. Across the 8 ruminant hosts, we identified a total of 17,349 vitamin-synthesizing microbes spanning 9 biosynthetic pathways (thiamine, riboflavin, niacin, pantothenate, pyridoxine, biotin, folate, cobalamin, and menaquinone). These microbes exhibited unified pathway selection patterns consistent with those in the human gut microbiome. Furthermore, within the major vitamin-synthesizing pathways commonly selected across ruminants, vitamin-synthesizing microbes displayed concentrated co-selection of specific functional gene nodes, revealing that despite taxonomic differences among gastrointestinal vitamin-synthesizing communities, they share highly convergent pathway preferences and common node-level selection patterns.

**Conclusions:**

Together, by reconstructing the ruminant GIT microbiome reference genome catalog (CRMC), we elucidated the core microbial taxa and their functional features across ruminants, as well as the pathway preferences and distribution patterns of vitamin-synthesizing microbes. These findings provide an effective reference for advancing ruminant GIT microbiome research, offering gene co-selection insights for microbial synthetic biology design, and guiding microbiome-based interventions in ruminant systems.

## Introduction

Ruminants possess a highly specialized gastrointestinal tract (GIT) system that efficiently converts low-quality forage into high-value products such as milk and meat, conferring substantial economic importance and representing one of the most intricate symbiotic systems in nature [1–5]. The ruminant GIT microorganisms include bacteria, archaea, anaerobic fungi, protozoa, and viruses [4, 5]. These microorganisms cooperatively deconstruct cellulose and lignin, depolymerize plant polysaccharides, and ferment soluble substrates [6–8]. The ruminant GIT microorganisms also synthesize microbial protein, a wide spectrum of volatile fatty acids, and essential micronutrients such as B-vitamins and vitamin K₂, thereby supporting host nutritional balance and reducing the need for exogenous supplementation [9–11]. Together, the unique anatomical structure and microbial features of the ruminant GIT confer remarkable roughage tolerance, digestive capacity, and nutritional value.

Ruminants, with their exceptionally rich GIT microbial reservoirs, have inspired diverse research perspectives [3, 5–8, 12]. In our previous work, we established reference catalogs of the intestinal microbiota in buffalo and goats, and elucidated that microbial community structures in ruminants are markedly shaped by intestinal location and dietary composition [6, 7]. Similarly, Fei et al. (2021) constructed a comprehensive ruminant microbial genome catalog from 370 metagenomic samples, comprising nearly 10,000 metagenome-assembled genomes (MAGs), which provided a foundational framework for exploring ruminant GIT microbiota [4]. Nevertheless, given the current scale of ruminant microbiome research, there remains a pressing need for reference collections with larger sample sizes and broader species coverage, encompassing independent microbial genomes from multiple ruminant hosts to enable deeper exploration of microbiome diversity and function.

Vitamins are indispensable cofactors and regulators in numerous physiological processes, including energy metabolism, immune modulation, and cellular homeostasis, and their adequate supply is essential for growth, reproduction, and health maintenance in mammals [13–17]. Increasing evidence has shown that the GIT microbiota contributes substantially to vitamin provisioning: specific bacterial taxa are capable of synthesizing B-group vitamins and vitamin K, thereby complementing dietary intake and supporting host nutritional balance [18–21]. In ruminants, rumen microbes play a pivotal role in the production of water-soluble B vitamins and vitamin K₂. Jiang et al. (2022) revealing that most microbial genomes possess only limited biosynthetic capacity and that cobalamin synthesis is particularly sensitive to dietary composition, being inhibited under high-grain diets [9]. However, a systematic study of vitamin-synthesizing bacteria based on a more comprehensive reference genome catalog of the ruminant GIT microbiome is still lacking, limiting our understanding of their ecological distribution and functional contributions.

We reconstructed a comprehensive ruminant microbial genome catalog (CRMC) based on 2,333 metagenomic samples, comprising 39,696 MAGs, together with species-specific reference catalogs for 8 ruminant hosts (buffalo, cattle, goat, sheep, yak, roe deer, water deer, and moose) generated under a unified analytical framework. This effort fills a critical gap in genomic resources and enables robust cross-species comparisons of microbial composition and functional repertoires, revealing both conserved and host-specific features. Building on these standardized catalogs, we further characterized the distribution of vitamin-synthesizing microbes, identifying consistent biosynthetic pathways and core metabolic preferences across ruminant’s vitamin-synthesizing microbes and found that humans exhibited the same vitamin biosynthetic pathway preferences as observed in ruminants. Overall, the CRMC provides an updated and robust genomic foundation for ruminant GIT microbiome research, while the systematic mapping of vitamin-synthesizing taxa yields novel insights into their ecological roles and establishes fundamental genomic principles of vitamin-synthesizing microbes derived from natural ruminant GIT, thereby informing functional microbiota mining, synthetic community design, and synthetic biology applications.

## Methods

### Data collection, quality control, and host/food genome removal

**Figure 1:**
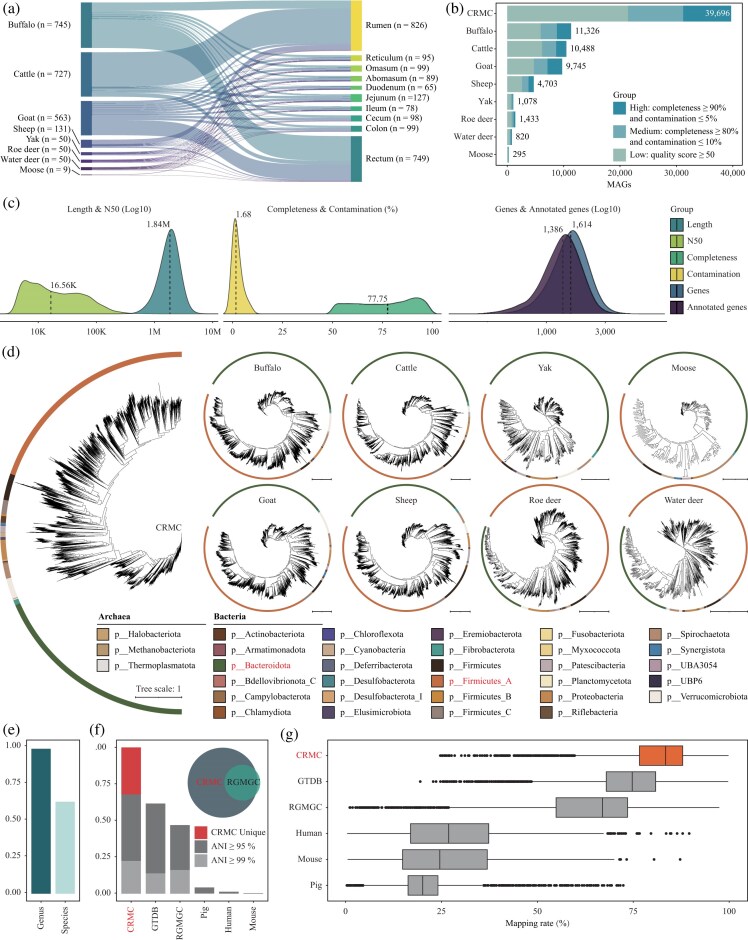
Construction and evaluation of the ruminant GIT microbiome reference genome catalog (CRMC). (a) Distribution of 2,325 metagenomic samples from 8 ruminant hosts across 10 GIT regions. Connections indicate host–GIT site relationships, with numbers in parentheses representing sample counts. The GIT sites were divided into three sections: stomach (rumen, reticulum, omasum, and abomasum), small intestine (duodenum, jejunum, and ileum), and large intestine (cecum, colon, and rectum). (b) Quality distribution of the CRMC and host-specific reference genome catalogs reconstructed in parallel. Numbers indicate the total MAGs in each catalog. Quality thresholds were defined following Bowers et al. [71]. Different colors represent MAG quality: high-quality (≥90% completeness and ≤5% contamination), medium-quality (≥80% completeness and ≤10% contamination), and low-quality (≥50% completeness). (c) Distribution of CRMC MAGs length, N50, completeness, contamination, number of protein-coding genes, and number of annotated genes. Different colors represent distinct categories, and dashed lines indicate medians. (d) Taxonomic classification of the CRMC and host-specific catalogs based on GTDB-Tk [60]. (e) Taxonomic classification rates of the CRMC MAGs at genus and species levels according to GTDB-Tk [60]. (f) Pairwise ANI [69] comparisons (95% and 99%) between the CRMC and selected public datasets (GTDB-db [60], RGMGC [4], pig [66], human [67], and mouse [68]). The Venn diagram shows the coverage of the RGMGC (based on 370 samples) [4] by the CRMC at the species level (ANI 95%). (g) Reads mapping rates of 2,325 ruminant metagenomic clean data against the CRMC and selected public datasets (GTDB-db [60], RGMGC [4], pig [66], human [67], and mouse [68]).

To comprehensively reconstruct a reference genome catalog for ruminant GIT microbiota, we collected 2,325 publicly available metagenomic samples from 21 NCBI projects (Supplement Table S1) [4, 6, 7, 22–37]. These samples covered 10 GIT regions (rumen, reticulum, omasum, abomasum, duodenum, jejunum, ileum, cecum, colon, and rectum) from 8 representative ruminant species (buffalo, cattle, goat, sheep, yak, roe deer, water deer, and moose; Fig. 1a; Supplement Table S1). Raw reads were processed using Trimmomatic (v0.39) [38] with the parameters “ILLUMINACLIP:TruSeq3-PE.fa:2:30:10 SLIDINGWINDOW:4:15 MINLEN:50 LEADING:3 TRAILING:3” to remove low-quality bases and adapter sequences. To eliminate host- and diet-associated contamination, quality-filtered reads were mapped against a set of host and food reference genomes using Bowtie2 (v2.3.5.1) [39] with the “–very-sensitive” parameter. The reference genome panel included *Capra hircus* (GCF_001704415.1) [40], *Bubalus bubalis* (GCA_004794615.1) [41], *Camelus bactrianus* (GCF_000767855.1) [42], *Camelus dromedarius* (GCF_000803125.2) [43], *Bos taurus* (GCF_002263795.1) [44], *Alces alces* (GCA_007570765.1) [45], *Cervus elaphus* (GCF_910594005.1) [46], *Rangifer tarandus caribou* (GCA_019903745.1) [47], *Capreolus capreolus* (GCA_000751575.1) [48], *Ovis aries* (GCF_016772045.1) [49], *Hydropotes inermis* (GCA_020226075.1) [50], *Bos grunniens* (GCA_005887515.2) [51], as well as ruminant food genomes including *Glycine max* (GCF_000004515.6) [52], *Zea mays* (GCF_902167145.1) [53], and *Medicago truncatula* (GCF_003473485.1) [54]. The remaining reads after decontamination were defined as clean data and used for subsequent analyses.

### Assembly, binning, quality assessment, dereplication, and construction of the CRMC and species-specific catalogs

Each clean metagenomic sample was processed using identical parameters, with unspecified settings kept at their defaults (Supplementary Fig. 1):

First, assemblies were generated with MEGAHIT (v1.2.8) [55] using the parameter “–min-contig-len 1000” to retain contigs of at least 1 kb.

Second, contig binning was performed by mapping reads back to assemblies with BWA-MEM (v0.7.17) and calculating depth profiles with Samtools (v1.9) [56] and the “jgi_summarize_bam_contig_depths” function of MetaBAT2 (v2.12.1) [57], followed by binning.

Third, genome bins were subjected to quality filtering and dereplication using CheckM (v1.1.1) [58] (completeness ≥ 50%, contamination ≤ 10%) and dRep (v2.3.2) [59] (strain-level dereplication at ANI 99%) to construct the comprehensive ruminant microbial genome catalog (CRMC).

Due to the large number of genomes generated by the initial binning, we first classified all bins taxonomically with GTDB-Tk (v1.2.0) [60] and then applied dRep (v2.3.2) [59] dereplication at the genus level to reduce redundancy at the strain level. In parallel, we independently reconstructed species-specific microbial genome catalogs for the 8 ruminant hosts by applying the same quality filtering and dereplication strategy as used for the CRMC.

After dereplication, the CRMC contained a total of 16,431 MAGs at the species-level and 39,696 MAGs at the strain-level. The species-specific catalogs contained 11,326 MAGs in buffalo, 10,488 in cattle, 9,745 in goat, 4,703 in sheep, 1,078 in yak, 1,433 in roe deer, 820 in water deer, and 296 in moose, respectively. These catalogs were subsequently used for cross-species comparisons of microbial composition and functional repertoires.

### Taxonomic annotation, gene prediction, and functional annotation

To determine the taxonomic composition and functional potential of the CRMC and the 8 species-specific ruminant microbial genome catalogs, we applied a uniform annotation pipeline. Each MAG was taxonomically classified using the “classify_wf” workflow of GTDB-Tk (v1.2.0) [60], and phylogenetic relationships were inferred with the “infer” function. The resulting phylogenetic trees, together with annotation metadata, were visualized and edited in iTOL (v6) [61].

For functional annotation, protein-coding genes were predicted from each MAG using Prokka (v1.14.5) [62]. Predicted proteins were subsequently annotated with eggnog-mapper (v2.1.12) [63] for KEGG pathways and COG functions, run_dbcan (v4.1.0) [64] for CAZy family carbohydrate-active enzymes, barrnap (v0.9) for rRNA genes, and tRNAscan-SE (v2.0.12) [65] for tRNA structures. This combined pipeline provided comprehensive functional and structural annotations for downstream analyses of ruminant microbial genomes.

### Comparative evaluation with public genome catalogs and reads mapping rate analysis

To evaluate the novelty of the CRMC, we calculated pairwise average nucleotide identity (ANI) between MAGs in the CRMC and those in existing public datasets, including GTDB-db [60], RGMGC [4], pig [66], human [67], and mouse [68] catalogs, using fastANI (v1.1) [69]. At ANI thresholds of 95% and 99%, we counted the number of MAGs shared between the CRMC and each public dataset and further calculated the proportion of MAGs unique to the CRMC.

Moreover, we compared the read mapping rates between the CRMC and public datasets. Clean reads from 2,325 ruminant GIT metagenomic samples were aligned against the CRMC and each public catalog using BWA-MEM (v0.7.17), and mapping rates were calculated with Samtools (v1.9) [56] to provide a direct measure of the enhancement offered by the CRMC over public datasets.

### Cross-species comparison of microbial composition and functions

To compare the commonalities and differences of GIT microbiomes across ruminant hosts, we analyzed the microbial community structure and functional features based on the 8 species-specific genome catalogs. Pairwise ANI between MAGs in each host-specific catalog and those in the other seven hosts was calculated using fastANI (v1.1) [69], with MAGs defined as shared or host-specific at an ANI threshold of 99%.

To identify taxa that tend to be shared or specific to ruminant hosts, Fisher tests were performed based on the phylum-level taxonomic annotations of MAGs. A significance threshold of P value ≤ 0.05 was applied to determine whether specific phyla were significantly enriched in either shared or host-specific groups.

Furthermore, Fisher tests (*P-*value ≤ 0.05) were performed based on substrate annotations of protein-coding genes from shared and host-specific MAGs to evaluate whether carbohydrate utilization patterns displayed commonalities or host-specific characteristics across ruminants.

### Relative abundance estimation of MAGs

To obtain more accurate estimates of relative abundance, we calculated species-specific MAG abundances for each ruminant host using a unified pipeline. First, reference indexes were built for each catalog with the “sketch” function of Sylph (v0.6.1) [70]. Next, the “profile” function of Sylph (v0.6.1) [70] was applied to estimate the relative abundance of MAGs in each catalog. Unlike coverage-based approaches, the k-mer–based strategy implemented in Sylph enables both efficient and precise resolution of MAG relative abundances, thereby improving the accuracy of cross-sample and cross-species comparisons.

### Identification and abundance estimation of vitamin-synthesizing microbes

To identify vitamin-synthesizing microbes and estimate their relative abundances in each ruminant GIT, we combined functional annotations of host-specific microbial genome catalogs with KEGG vitamin biosynthesis pathways (Table S2). Candidate vitamin-synthesizing MAGs were defined according to the following criteria (the same screening workflow was applied to the human IMGG dataset):

1. Only MAGs encoding the full complement of genes for an entire vitamin biosynthesis pathway were considered as vitamin-synthesizing candidates. Specifically, the identification criteria required the presence of all pathway genes within a single MAG, but not necessarily on a single contig.
2. For intermediate pathway nodes involving multiple genes, only MAGs containing at least one of the relevant genes were considered to encode the corresponding function.
3. Based on the relative abundance matrices of each host-specific microbial genome catalog, MAG abundances associated with the same vitamin biosynthesis pathway were summed to obtain the total relative abundance of vitamin-synthesizing microbes for that pathway.

This approach allowed us to systematically extract vitamin-synthesizing MAGs and quantify their contributions to vitamin biosynthesis across different ruminant hosts.

### Regional distribution of vitamin-synthesizing microbes within ruminant GITs

To further investigate the distribution of vitamin-synthesizing microbes across GIT regions in different ruminant hosts, we partitioned the identified vitamin-synthesizing MAGs according to host species, GIT location, and vitamin biosynthesis type. The relative abundance distributions of vitamin-synthesizing microbes were then characterized across different hosts, GIT regions, and biosynthetic pathways. Relative abundance values were obtained from the standardized MAG abundance matrices described above. Visualization of distribution patterns was performed using the ggridges (v0.5.7) package in R.

### Frequency analysis of biosynthetic pathway nodes in vitamin-synthesizing microbes

To further characterize the usage of multi-gene nodes within vitamin biosynthesis pathways across different ruminant hosts, we quantified the proportions of protein-coding genes in vitamin-synthesizing MAGs mapped to each pathway node. For each ruminant host and vitamin biosynthesis pathway, we calculated both (i) the number of MAGs assigned to the pathway and (ii) the proportion of MAGs containing genes corresponding to each multi-gene node.

Pathway structures were obtained from KEGG vitamin biosynthesis pathways (Table S2). Visualization of pathway node usage patterns was conducted using the pheatmap (v1.0.12) package in R.

### Pattern analysis of vitamin-synthesizing microbes biosynthetic pathway node preferences across ruminants

Based on the node usage frequencies of vitamin biosynthesis pathways, we further analyzed the co-selection patterns of multi-gene pathway nodes within individual MAGs and characterized how these patterns varied across different ruminant hosts. The analysis consisted of two steps:

1. For each vitamin-synthesizing MAG, gene information corresponding to selected pathway nodes was extracted, and MAGs carrying the same set of node genes were assigned to the same pattern category.
2. For each ruminant host and vitamin biosynthesis pathway, we calculated both the number and relative abundance of MAGs belonging to each pattern category.

Visualization of the patterns of multi-gene node usage by vitamin-synthesizing microbes across different ruminant hosts was carried out using the ggplot2 (v3.5.1) package in R.

### Statistics

In addition to the software, data filtering and processing were performed using custom Perl and R scripts. Unless otherwise specified, statistical significance between groups was assessed using the Wilcoxon rank-sum test. All statistical analyses were conducted on the dataset comprising 2,325 ruminant GIT metagenomic samples. The software commands and scripts used in this study are available on GitHub (see the “Availability of source code and requirements” section).

## Results

### Reconstruction of the comprehensive ruminant microbial catalog

To enable consistent functional annotation of microbial genomes of the ruminant GIT, we reconstructed the microbial genomes using 2,325 metagenome data from 8 ruminant hosts (buffalo, cattle, goat, sheep, yak, roe deer, water deer, and moose; Fig. 1a; Supplement Table S1) covering 10 GIT regions (rumen, reticulum, omasum, abomasum, duodenum, jejunum, ileum, cecum, colon, and rectum; Fig. 1a; Supplement Table S1). All samples were processed through a unified pipeline, which involved the removal of vector sequences, low-quality bases, short reads, and host/food genomes.

After assembly, binning, quality control (with completeness ≥ 50%, contamination ≤ 10%, and contig length ≥ 200 kb), and strain-level dereplication (99% ANI), we obtained a Comprehensive Ruminant Gastrointestinal Tract Microbiome Reference Genome Catalog (CRMC) consisting of 16,431 species-level MAGs and 39,696 strain-level MAGs (Fig. 1b; Supplementary Fig. 1). In parallel, we also constructed host-specific microbial genome catalogs for each of the 8 ruminants (Fig. 1b). The median size of the CRMC MAGs was 1.84 Mb (235.18 kb ~ 8.36 Mb; Fig. 1c), with a median N50 of 16.56 kb (3.30 kb ~ 1.17 Mb; Fig. 1c). The median completeness was 77.75%, and the median contamination was 1.68% (Fig. 1c). The median number of protein-coding genes per MAG was 1,614 (231 ~ 8,083), of which 1,386 (186 ~ 7,324) were functionally annotated, accounting for approximately 85.87% of the protein-coding genes (Fig. 1c). Approximately 93.74% of the MAGs contained multiple tRNA genes (≥10 types), with an average of 15.37 tRNA types per MAG. According to the quality standards defined by Bowers et al. [71], approximately 46.06% of the CRMC MAGs were classified as medium-to-high quality (Fig. 1b), 24.64% (9,781 MAGs) classified as medium-quality (completeness > 80%, contamination < 10%; Fig. 1b), and 21.42% (8,502 MAGs) classified as high-quality (completeness > 90%, contamination < 5%; Fig. 1b).

Taxonomic annotation of the CRMC was performed using the GTDB database, revealing that the catalog consists of 981 archaea MAGs (2.47%) and 38,715 bacteria MAGs (97.53%). The archaeal MAGs mainly belong to three phyla: Halobacteriota (predominantly family Methanocorpusculaceae and genus *Methanocorpusculum*), Methanobacteriota (predominantly family Methanobacteriaceae and genus *Methanobrevibacter*), and Thermoplasmatota (predominantly family Methanomethylophilaceae and genus *UBA71*). Among the bacterial phyla, Firmicutes_A and Bacteroidota were overwhelmingly dominant, with 15,252 MAGs (38.42%) and 14,305 MAGs (36.03%), respectively, comprising most of the ruminant GIT microbiome (Fig. 1d). These two phyla were followed at much lower numbers by Firmicutes (1,826 MAGs, 4.60%), Verrucomicrobiota (1,429 MAGs, 3.60%), and Proteobacteria (1,371 MAGs, 3.45%). We found that the Firmicutes_A phylum was entirely composed of the Clostridia class, with most of its MAGs belonging to the orders Oscillospirales (7,865 MAGs, 51.57%), Lachnospirales (3,461 MAGs, 22.69%), and Christensenellales (2,427 MAGs, 15.91%), collectively accounting for 90.17% of the Firmicutes_A phylum. Similarly, the Bacteroidota phylum was entirely composed of the Bacteroidia class, with the order Bacteroidales (14,258 MAGs, 99.67%) forming the core of the phylum (Supplement Table S2). These findings further underscore the central role of the Oscillospirales, Lachnospirales, Christensenellales, and Bacteroidales orders in the ruminant GIT microbiome. Interestingly, although Bacteroidota and Firmicutes_A were the dominant phyla in all 8 ruminant hosts GIT microbiomes, their diversity varied across species (Supplementary Figs 2, 3; Supplement Tables S3–S10). Notably, the dominant phyla Bacteroidota and Firmicutes_A exhibited relatively stable proportions in confinement-fed ruminants but showed pronounced fluctuations in free-ranging species. The ratio of Firmicutes_A to Bacteroidota MAGs remained close to 1.1 in confinement-fed ruminants (buffalo, 0.93; cattle, 0.98; sheep, 1.23; goat, 1.28; Supplement Table S11). In contrast, this ratio varied markedly among free-ranging ruminants: large-bodied species such as yak (0.36) and moose (0.32) displayed higher proportions of Bacteroidota, whereas small-bodied species such as roe deer (2.18) and water deer (2.08) exhibited greater abundances of Firmicutes_A (Fig. 1d; Supplement Table S11). These results suggest that confinement-fed ruminants maintain a more balanced ratio of dominant phyla due to the stability of their feed composition, while in free-ranging ruminants, body size–associated differences in intestinal surface area may contribute to the observed divergence.

The CRMC significantly expanded the ruminant MAGs by demonstrating high coverage over existing MAG catalogs and containing additional novel ones. Specifically, at the species level (ANI 95%), the CRMC nearly encompasses the entire RGMGC catalog (8,881 MAGs constructed from 370 ruminant GIT microbiome samples [4], 85.62%; Fig. 1f). Additionally, CRMC contains 12,756 (32.13%) novel ones that shared ≤ 95% ANIs with the microbial genomes in major public database (GTDB and RGMGC) and of model species (human, mouse, pig) (Fig. 1f). Consistently, the novelty of the CRMC was further supported by the presence of 15,059 unannotated MAGs (37.94%) according to the GTDB database, which showed low similarity to existing reference genomes (ANI ≤ 95%; Fig. 1e).

As expected, the CRMC catalog showed the highest representation of the 2,325 metagenomic samples by recruiting ~83.45% of clean sequencing reads, significantly higher than any other datasets such as GTDB (74.75%), RGMGC (66.99%), and others (Fig. 1g; Supplement Table S12).

### Shared and distinctive microbial taxonomical and functional capacities across ruminant hosts

**Figure 2:**
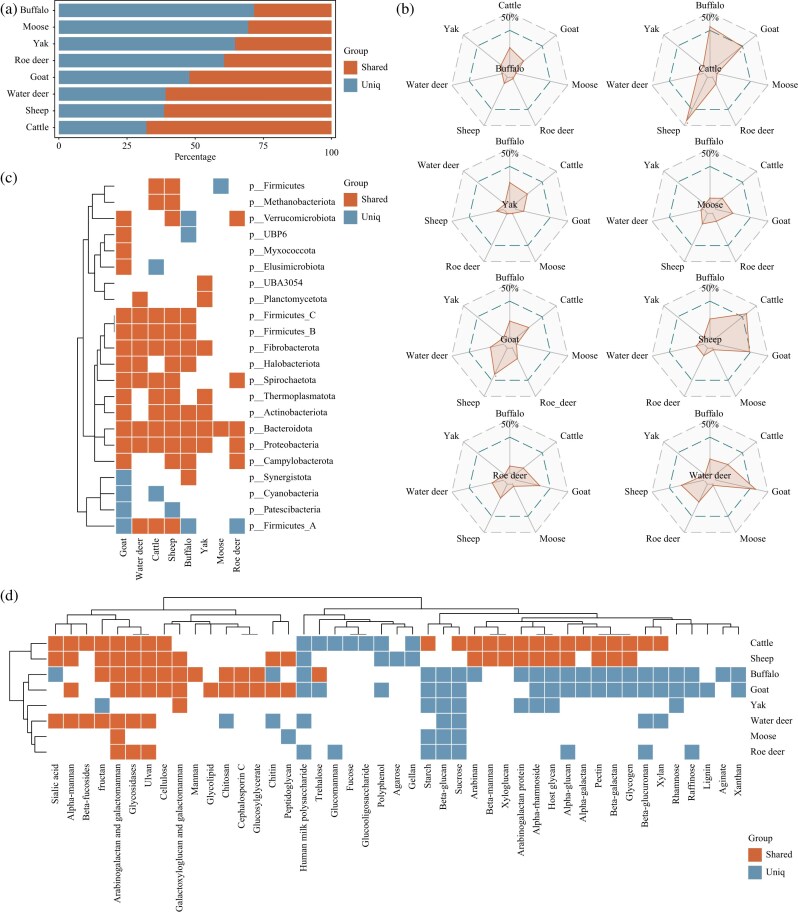
Cross-host comparisons of the ruminant GIT microbiome. (a) Proportions of shared and host-specific MAGs at the species level across different ruminants. Species-level comparisons were defined using an ANI threshold of 95%. MAGs with ANI ≥95% to at least one other ruminant were considered shared, whereas MAGs below this threshold were classified as unique. (b) Radar plot showing the proportion of shared taxa within each ruminant host relative to its own MAG collection. Shared MAGs were defined same as (a). Shaded areas indicate the proportion of shared MAGs. (c) Distribution of significantly shared and host-specific phyla among ruminants. Statistical criteria for defining significantly shared or specific phyla are described in the Methods. (d) Distribution of significantly shared and host-specific protein-coding genes related to substrate utilization among ruminants. Statistical criteria for defining significantly shared or specific substrate-utilizing genes are described in the Methods.

To further explore the compositional and functional similarities and differences among ruminant GIT microbiomes, we performed a cross-species comparison based on the independently reconstructed reference genome catalogs of 8 ruminant hosts. Using 95% ANI as the species-level threshold, each host-specific catalog was compared with those of the other seven hosts (Supplementary Fig. 4; Supplement Table S13). The results showed that cattle had the highest proportion of shared species with other ruminants (6,850 MAGs, 65.31%; Fig. 2a), followed by sheep (2,711 MAGs, 57.64%; Fig. 2a) and goat (4,691 MAGs, 48.14%; Fig. 2a), both of which are typically fed under more confinement feeding. In contrast, buffalo, which are more commonly raised under dispersed or semi-free-range conditions, exhibited the lowest proportion of shared species (2,822 MAGs, 24.92%; Fig. 2a). These findings suggest that indoor feeding may drive a certain degree of convergence in the GIT microbiomes of ruminants, whereas grazing or free-range management tends to preserve host-specific microbial lineages.

We further examined the distributional features of microbial taxa shared between each ruminant GIT microbiome and those of the other hosts. Among the 8 ruminant hosts, cattle exhibited the highest proportion of shared MAGs, with the majority overlapping with other ruminants raised under indoor feeding, including sheep (45.05%; Fig. 2b), goat (32.62%; Fig. 2b), and buffalo (36.08%; Fig. 2b). In contrast, similarity between cattle and the other hosts was much lower, including roe deer (7.03%; Fig. 2b), water deer (6.11%; Fig. 2b), yak (2.73%; Fig. 2b), and moose (1.22%; Fig. 2b). Smaller-bodied ruminants (goat, sheep, roe deer, and water deer) also shared a moderate proportion of microbial taxa, with goat and sheep exhibiting nearly 50% overlap (Fig. 2b). Roe deer and water deer further shared 23.03% and 38.05% of taxa with goat (Fig. 2b), respectively. Consistent with the slightly higher representation of Firmicutes_A in smaller-bodied ruminants, these results suggest that host body size may partially shape the similarity of GIT microbiomes. By contrast, free-ranging or wild ruminants, such as buffalo, yak, and moose, shared only a limited set of core symbionts with the other hosts (<25%; Fig. 2b), highlighting the role of feeding practices in maintaining host-specific microbial lineages.

Based on the shared and host-specific microbial taxa among the 8 ruminant hosts, we further investigated the distribution of bacterial and archaeal phyla as well as functional pathways associated with substrate utilization (Supplementary Fig. 5). Notably, the dominant phylum Bacteroidota exhibited a significant trend of being shared across all hosts (Fig. 2c), likely reflecting its critical role in the degradation of fibrous plant materials in the ruminant foregut. In addition, most hosts showed significant sharing of phyla, including Firmicutes_C, Firmicutes_B, Fibrobacterota, Spirochaetota, Actinobacteriota, Proteobacteria, and the archaeal groups Thermoplasmatota and Halobacteriota (Fig. 2c), which collectively encompass the core taxa responsible for carbohydrate digestion, nutrient absorption, and methane production. By contrast, the dominant phylum Firmicutes_A was significantly shared only among cattle, sheep, and water deer (Fig. 2c), a pattern that may be associated with its variable representation between large- and small-bodied ruminants. Furthermore, Firmicutes and the methanogenic archaeal phylum Methanobacteriota were significantly shared only between cattle and sheep (Fig. 2c), suggesting that, beyond the core phyla, the GIT microbiomes of these indoor feeding ruminants have developed additional convergence in functional groups linked to digestion and methanogenesis.

Further analysis of protein-coding genes from shared and host-specific MAGs elucidated clear substrate utilization patterns across the 8 ruminant hosts. Substrates that exhibited a clear trend of being commonly utilized across most ruminant GIT microbiomes included plant-derived polysaccharides such as alpha-mannan, fructan, arabinogalactan, galactomannan, ulvan, cellulose, and galactoxyloglucan, together with glycosidases involved in polysaccharide breakdown (Fig. 2d). These findings indicate that the core functional traits of shared microbial taxa in ruminant GIT microbiomes are primarily centered on the utilization and absorption of dietary polysaccharides. In contrast, ruminants raised under indoor feeding, particularly cattle and sheep, displayed unique patterns of convergence involving substrates enriched in plant-derived polysaccharides (arabinan, beta-mannan, xyloglucan, arabinogalactan protein, alpha-glucan, pectin, beta-galactan, and glycogen) as well as small-molecule derivatives such as alpha-rhamnoside, which in most other ruminant hosts tended to occur as host-specific features (Fig. 2d). Notably, some substrates commonly present in formulated feeds [72–74], including beta-mannan, pectin, beta-galactan, alpha-glucan, and glycogen, were exclusively shared between cattle and sheep (Fig. 2d). These results suggest that, in addition to convergent features in digestion and methanogenesis, indoor feeding cattle and sheep have also developed distinctive convergence in the utilization of plant-derived polysaccharides, differentiating them from other ruminant hosts.

### Consistent strategies for vitamin biosynthesis among ruminant GIT microbes

**Figure 3:**
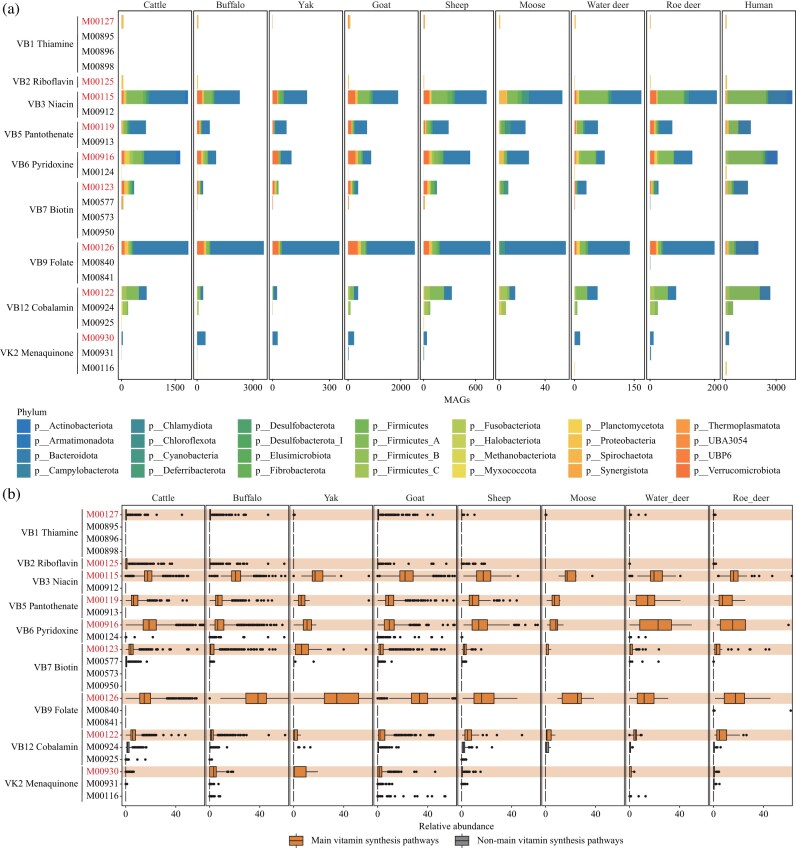
Identification and functional pathway assessment of vitamin-synthesizing microbes in the ruminant GIT microbiome. (a) Distribution of vitamin-synthesizing MAGs across the 8 ruminant hosts. Pathway information was obtained from the KEGG database (Table S2). Vitamin-synthesizing MAGs were defined as those containing the complete set of pathway nodes, with multi-gene nodes required to include at least one functional enzyme. Each color represents a different phylum, and bar length indicates the number of MAGs. The color coding for phyla is consistent between the ruminant and human datasets. (b) Relative abundance of vitamin-synthesizing MAGs assigned to each pathway across different ruminant hosts. Relative abundances were calculated by aggregating the total abundance of MAGs assigned to the corresponding pathway shown in panel a. Each point represents clean data from an individual ruminant GIT sample.

To characterize vitamin-synthesizing microbes in the ruminant GIT microbiome, we identified taxa carrying at least one complete biosynthetic pathway based on KEGG vitamin-synthesizing modules (thiamine, riboflavin, niacin, pantothenate, pyridoxine, biotin, folate, cobalamin, and menaquinone; Supplement Table S14). The vitamin-synthesizing microbes in the ruminant GIT were mainly derived from the dominant phyla Bacteroidota and Firmicutes_A, with additional contributions from Verrucomicrobiota, indicating that these major lineages not only function in digestion and nutrient absorption but also play important roles in host vitamin provisioning (Fig. 3a; Supplement Table S15).

Across the 8 ruminants, we observed a consistent selection of the same major biosynthetic pathways by vitamin-synthesizing microbes. For thiamine, riboflavin, niacin, pantothenate, pyridoxine, and folate, nearly all vitamin-synthesizing microbes relied exclusively on a single biosynthetic pathway (M00127, M00125, M00115, M00119, M00916, and M00126, respectively; Fig. 3a; Supplement Table S15). Similarly, although multiple KEGG modules exist for biotin, cobalamin, and menaquinone biosynthesis, the microbes consistently converged on one major pathway (M00123, M00122, and M00930, respectively; Fig. 3a; Supplement Table S15). Similarly, human gut vitamin-synthesizing microbes also exhibited a unified pathway preference for each vitamin, suggesting selection on convergent vitamin biosynthesis functions across ruminants and human.

Despite this functional convergence, the taxonomic composition of vitamin-synthesizing microbes varied among hosts. In line with overall differences in microbial community structure, smaller-bodied ruminants harbored a relatively higher proportion of Firmicutes_A vitamin-synthesizers compared to larger-bodied hosts, whereas Bacteroidota were more dominant in larger-bodied hosts (Fig. 3a). Notably, the human gut microbiome also exhibited a higher representation of Firmicutes_A vitamin-synthesizers (Fig. 3a), paralleling patterns observed in smaller-bodied ruminants.

To further investigate the pathway selection preferences of different vitamin biosynthetic routes across the 8 ruminant hosts, we aggregated the relative abundances of these microbes for each pathway across the different ruminant hosts. The results elucidated that, at the relative abundance level, all ruminant hosts exhibited consistent core vitamin biosynthesis pathway preferences (Fig. 3a, b). Microbial taxa responsible for the synthesis of niacin, pyridoxine, and folate, which are essential for host nutrition, showed relatively high abundances (Fig. 3b). Notably, niacin and pyridoxine are vitamins that the host cannot synthesize on its own and relies on GIT microbes for supply. Although the number of vitamin-synthesizing MAGs varied across hosts, the overall abundance of microbes corresponding to each biosynthetic pathway remained similar among the ruminants (Fig. 3b; Supplementary Fig. 10). This indicates an interaction potential between the ruminant host and its GIT vitamin-synthesizing microbiota, maintaining a stable level of microbial abundance to meet the host’s nutritional needs.

### Abundance of vitamin-synthesizing microbes varies across intestinal compartments and ruminant hosts

**Figure 4:**
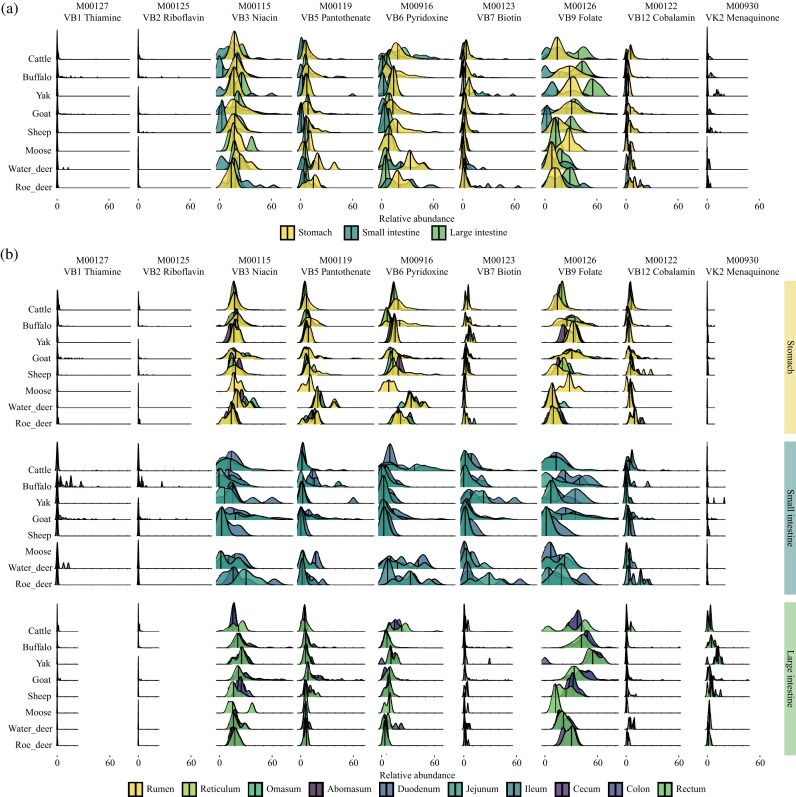
Distribution of vitamin-synthesizing microbes across ruminant GIT regions. (a) Distribution of vitamin-synthesizing microbes in the stomach, small intestine, and large intestine across different ruminant hosts. Samples were grouped into three GIT sections: stomach (rumen, reticulum, omasum, and abomasum), small intestine (duodenum, jejunum, and ileum), and large intestine (cecum, colon, rectum). Different colors represent the major GIT sections. Vertical lines indicate median values. (b) Distribution of vitamin-synthesizing microbes across 10 GIT locations in ruminants. Different colors represent individual GIT sites. Vertical lines indicate median.

To further investigate the distribution patterns of vitamin-synthesizing microbes across different ruminant hosts and GIT regions, we analyzed the relative abundance distribution of these microbes in 8 ruminant hosts across various GIT locations. The results showed that vitamin-synthesizing microbes with relatively low overall abundance, such as those for thiamine, riboflavin, and menaquinone, exhibited low relative abundance across all three GIT regions (stomach, small intestine, and large intestine; Supplementary Fig. 6). In contrast, vitamin-synthesizing microbes with higher overall abundance, such as those for niacin, pyridoxine, and folate, were more significantly differentiated across the GIT regions (Fig. 4a). Therefore, both the stomach and large intestine are key sites for vitamin-synthesizing microbe distribution in ruminants (Supplementary Figs 7–9). Notably, folate-synthesizing microbes, which had the highest relative abundance, were more abundant in the large intestine than the stomach in most ruminant hosts (Fig. 4a; Supplementary Figs 7–9). While the relative abundance of vitamin-synthesizing microbes across ruminant hosts was largely consistent, folate-synthesizing microbes showed a significantly higher abundance in the large intestine of larger-bodied ruminants, such as cattle, buffalo, and yak, compared to smaller-bodied species (Fig. 4a; Supplementary Figs 7–9). This suggests that the larger GIT volume in these larger ruminants may provide more ecological space for these microbes to thrive.

Further analysis of the 10 GIT locations in ruminants elucidated that vitamin-synthesizing microbes with low relative abundance, such as those for thiamine and riboflavin, were consistently distributed in the duodenum and jejunum of the small intestine across different ruminant hosts (Fig. 4b). Menaquinone-synthesizing microbes were predominantly found in the rectum of the large intestine, demonstrating their location-specific distribution (Fig. 4b). Vitamin-synthesizing microbes for niacin, pantothenate, pyridoxine, biotin, folate, and cobalamin, which were distributed across all 10 GIT locations, showed relatively stable abundance in the rumen, reticulum, omasum, and abomasum of the stomach (Fig. 4b). Biotin-synthesizing microbes were predominantly located in the small intestine (Fig. 4b). While folate-synthesizing microbes were more abundant in the large intestine than in the stomach and small intestine, cobalamin-synthesizing microbes were found at significantly lower levels in the large intestine compared to both the stomach and small intestine (Fig. 4b).

### Consistent node utilization within core vitamin biosynthetic pathways across ruminant hosts

**Figure 5:**
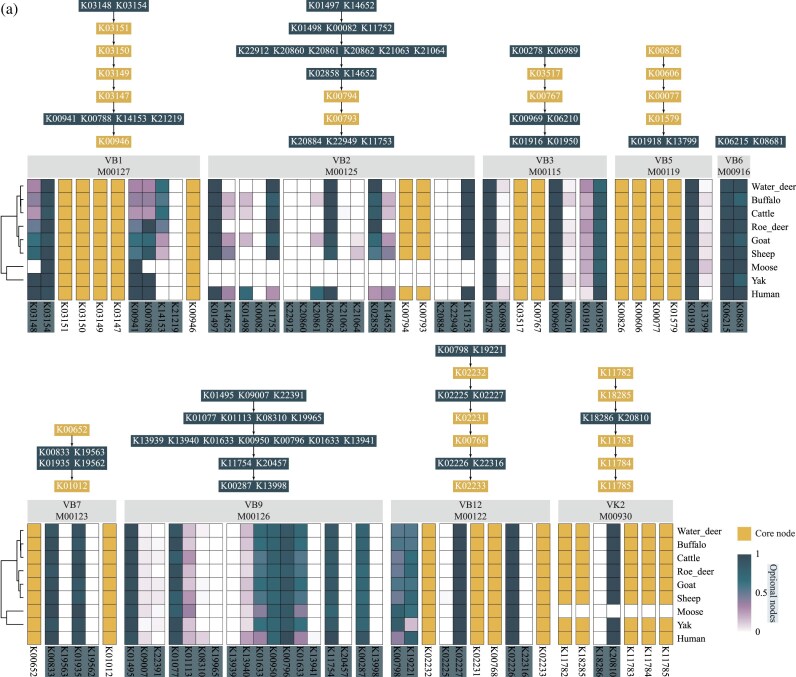
Usage frequency of protein-coding genes at pathway nodes in vitamin-synthesizing microbes across ruminant GIT microbiomes. Pathway information was obtained from the KEGG database (Table S2). The structures of the vitamin-synthesizing pathways follow the KEGG database. The heatmap color scale indicates the percentage of vitamin-synthesizing MAGs in the corresponding pathway that contained the respective node genes.

To investigate the characteristics of gene usage within vitamin biosynthesis pathways, we conducted a comprehensive analysis of protein-coding genes in vitamin-synthesizing microbes across the 8 ruminant hosts (Supplement Tables S15–S24). The results elucidated that, at multi-gene nodes where alternative enzymatic options exist, different ruminants exhibited distinct preferences. In the thiamine biosynthesis pathway, buffalo and cattle showed lower usage frequencies of K0218, K00941, and K00788 compared with goat and sheep, but a higher frequency of K12153 (Fig. 5a). Since K00941, K00788, and K12153 belong to the same multi-gene node, these vitamin-synthesizing microbes maintained functional capacity through compensatory preferences, which differed from the human gut microbiome, where K12153 was not utilized but K00941 and K00788 were frequently used (Fig. 5a). In the riboflavin biosynthesis pathway, buffalo, cattle, goat, and sheep, like humans, simultaneously utilized both K14652 and K01497 to enhance the efficiency of the multi-gene node, whereas the other ruminants relied exclusively on K01497 (Fig. 5a). In contrast, for the niacin, pantothenate, pyridoxine, biotin, folate, cobalamin, and menaquinone pathways, both ruminant and human vitamin-synthesizing microbes displayed consistent usage frequencies, showing common preferences for specific genes. In the biotin biosynthesis pathway, microbes consistently tended to utilize K00833 and K01935 while avoiding K19563 and K19562 (Fig. 5a).

### Convergent selection of key steps in microbial vitamin biosynthesis pathways in the ruminant GIT

**Figure 6:**
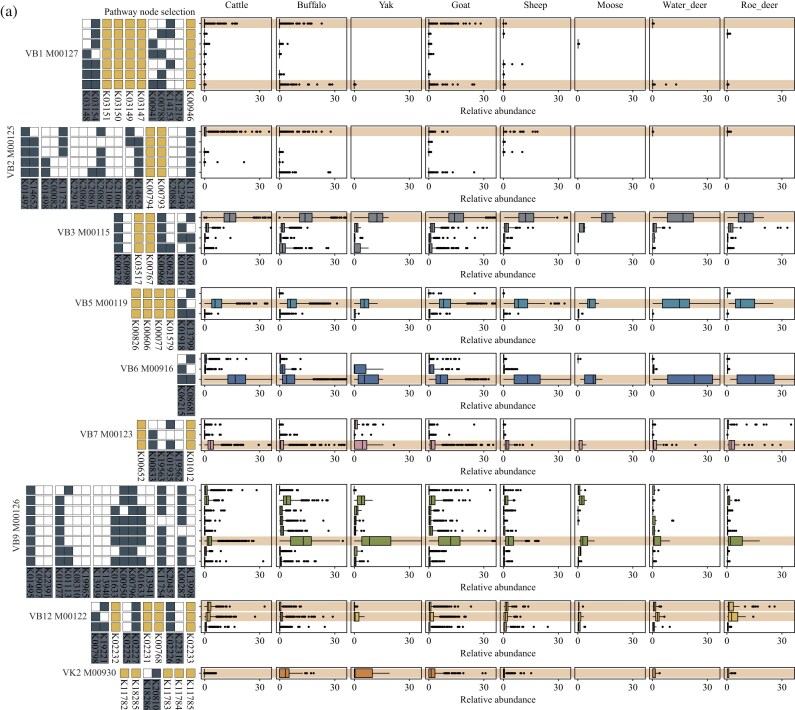
Relative abundance of co-selection patterns at multi-gene nodes in vitamin-synthesizing microbes across ruminant GIT microbiomes. Pathway information was obtained from the KEGG database (Table S2). The structures of the vitamin-synthesizing pathways follow the KEGG database. Relative abundance was calculated as the sum of MAGs harboring the same co-selected multi-gene nodes. Each point represents clean data from an individual ruminant GIT sample.

To further confirm the characteristics of multi-gene node co-selection within vitamin-synthesizing MAGs of the ruminant GIT microbiome, we categorized all vitamin biosynthesis pathways across the 8 ruminant hosts and aggregated the relative abundances of microbes that consistently selected the same multi-gene nodes. The results showed that nearly all vitamin-synthesizing microbes exhibited consistent co-selection of multi-gene nodes across ruminants (Fig. 6a; Supplementary Fig. 11). However, these co-selections did not necessarily involve choosing the maximum number of genes to ensure pathway efficiency. In the thiamine pathway, although genes such as K03148, K00941, and K00788 were additionally selected in vitamin-synthesizing MAGs, a higher relative abundance of microbes relied on K03154 and K14153 (Fig. 6a). Similarly, in the riboflavin pathway, while some microbes employed K14652, the majority primarily used K01497 (Fig. 6a). Comparable patterns were also observed in other pathways, including niacin (higher relative abundance of K01950 compared to K01916; Fig. 6a), pantothenate (K01918 compared to K13799; Fig. 6a), and folate (K01495, K01077, K01633, K00950, and K00796 compared to K09007, K22391, K01113, and K13940; Fig. 6a). In contrast, the pyridoxine and cobalamin pathways consistently exhibited multi-gene co-selection across ruminants, with pyridoxine-synthesizing microbes simultaneously selecting K06215 and K08681 (Fig. 6a), and cobalamin-synthesizing microbes selecting K00798 and K19221 (Fig. 6a).

Overall, we identified two major patterns: (i) thiamine, riboflavin, niacin, pantothenate, and folate pathways were characterized by the preferential selection of core multi-gene nodes without further expansion to additional redundant genes, and (ii) pyridoxine and cobalamin pathways involved multi-gene co-selection to enhance node functionality. Importantly, we observed that these patterns were consistent across all 8 ruminant hosts, and the relative abundances of microbes following each selection mode remained at similar levels, which highlights the functional stability of vitamin-synthesizing microbes across ruminant hosts, despite variations in GIT microbial diversity.

## Discussion

The ruminant-specific stomach provides a rich natural reservoir of microbial resources, which plays a critical role in digestion, nutrient absorption, and maintaining host homeostasis [1–5]. Although Fei et al. (2021) constructed a ruminant gastrointestinal tract microbiome genome catalog (RGMGC) based on 370 samples [4], given the current scale of ruminant microbiome research, there remains a pressing need for reference collections with larger sample sizes and broader species coverage.

To fill this gap, we comprehensively reconstructed the ruminant GIT microbiome reference genome catalog (CRMC) using 2,325 metagenomic clean data (from 21 NCBI projects; Supplement Table S1), which encompassed 8 ruminant hosts (buffalo, cattle, goat, sheep, yak, roe deer, water deer, and moose; Fig. 1a; Supplement Table S1) and across 10 GIT regions (rumen, reticulum, omasum, abomasum, duodenum, jejunum, ileum, cecum, colon, and rectum; Fig. 1a; Supplement Table S1). In parallel, we applied the same standardized analytical pipeline to independently generate species-specific reference genome catalogs for each host, enabling cross-host comparative analyses. The CRMC comprises 39,696 non-redundant MAGs (Fig. 1b), including 981 archaeal MAGs (2.47%) and 38,715 bacterial MAGs (97.53%; Fig. 1c). Although this study did not involve third-generation sequencing data, resulting in fewer MAGs reaching complete status, the CRMC not only nearly covered the existing RGMGC (8,881 MAGs, 85.62%; Fig. 1f) but also provided 15,059 MAGs (37.94%; Fig. 1e) lacking species-level annotations, and contributed 12,756 novel MAGs (32.13%; Fig. 1f) beyond current datasets. Furthermore, reads mapping rates improved markedly, from 66.99% with RGMGC to 83.35% with the CRMC (Fig. 1g), significantly expanding the scope of ruminant GIT microbiome reference systems and providing a direct and reliable dataset for future studies. In future studies, the incorporation of third-generation sequencing data will further enhance the CRMC, yielding more complete-level MAGs. Beyond serving as a comprehensive reference, the CRMC also provides a robust platform for functional microbiota mining, comparative host-microbiome analyses, and the rational design of synthetic microbial communities, thereby advancing both fundamental understanding of ruminant digestion and nutrient utilization and practical applications in animal husbandry.

Based on the reconstructed ruminant GIT microbiome reference genome catalog, we conducted cross-host comparative analyses among the 8 ruminant hosts to explore shared and host-specific microbial features.

First, we elucidated host body size- and diet-driven patterns of shared and host-specific microbial structures and functional features across the 8 ruminant hosts. Core phyla including Bacteroidota, Firmicutes_C, Firmicutes_B, Fibrobacterota, Spirochaetota, Actinobacteriota, and Proteobacteria were prevalently shared across ruminant GIT microbiomes (Fig. 2c). These phyla collectively underpin the essential functions of digestion and methanogenesis, whereas the dominant phylum Firmicutes_A was significantly shared only between cattle and sheep under indoor feeding conditions (Fig. 2c). At the functional level, the utilization of dietary polysaccharides such as alpha-mannan, fructan, arabinogalactan, galactomannan, ulvan, cellulose, and galactoxyloglucan, together with glycosidases involved in polysaccharide breakdown, constituted core features consistently shared across ruminants (Fig. 2d). By contrast, the utilization of arabinan, beta-mannan, xyloglucan, arabinogalactan protein, alpha-glucan, pectin, beta-galactan, glycogen, and small-molecule derivatives such as alpha-rhamnoside tended to show host-specific patterns (Fig. 2d). Notably, some substrates commonly present in formulated feeds, including beta-mannan, pectin, beta-galactan, alpha-glucan, and glycogen, were exclusively shared between cattle and sheep under indoor feeding conditions (Fig. 2d). These findings indicate that ruminants harbor a common set of core microbial taxa and functional genes. However, host body size and feeding practices can drive divergence in microbial composition and function. Under indoor feeding conditions, the frequent presence of feed-associated polysaccharides appears to have promoted functional convergence between cattle and sheep.

Second, we focused on the biosynthetic characteristics and GIT distribution of vitamin-synthesizing microbes across the 8 ruminant hosts. Vitamin-synthesizing microbes were primarily derived from Bacteroidota, Firmicutes_A, and Verrucomicrobiota, indicating that the dominant phyla Bacteroidota and Firmicutes_A not only participate in digestion and nutrient absorption but also play critical roles in microbial regulation of vitamin levels (Fig. 3a). Moreover, members of Verrucomicrobiota (Fig. 3a), including putative probiotics, may exert beneficial effects through their involvement in vitamin biosynthesis. Despite taxonomic differences in vitamin-synthesizing communities among ruminants (including comparisons with humans), microbes consistently selected the same pathway for each vitamin, and the total relative abundance of microbes within each biosynthetic route remained at similar levels (Fig. 3b). We speculate that this functional convergence is driven by shared environmental selective pressures within the gut, such as stable temperature, anaerobic conditions, and consistent physicochemical properties. Consequently, the vitamin-synthesizing bacteria that stably colonize these gut environments hold significant potential for identification and application as next-generation probiotics. This functional convergence highlights the stability of vitamin biosynthesis across diverse host-associated microbiomes. At the spatial level, analysis of vitamin-synthesizing microbes across different GIT locations elucidated distinct distributional patterns. Thiamine-, riboflavin-, and menaquinone-synthesizing microbes were predominantly distributed in the small intestine or large intestine (Fig. 4b). Biotin-synthesizing microbes were mainly concentrated in the small intestine, whereas cobalamin-synthesizing microbes were markedly less abundant in the large intestine compared with the stomach and small intestine (Fig. 4b). In contrast, vitamin-synthesizing microbes in the stomach were evenly distributed across all four compartments (Fig. 4b). Together, these findings demonstrate that while ruminant GIT microbiomes maintain stable overall vitamin biosynthetic potential, the distribution of vitamin-synthesizing microbes is location-specific. This underscores the importance of conducting targeted studies that account for the spatial distribution of vitamin-synthesizing microbes within the ruminant GIT.

Last, we further uncovered the node-level characteristics of vitamin-synthesizing microbes in the ruminant GIT, focusing on their selection preferences and co-selection patterns at multi-gene nodes. The results elucidated that, at multi-gene nodes where alternative enzymatic options exist, different ruminants exhibited distinct preferences. In the thiamine biosynthesis pathway, buffalo and cattle showed lower usage frequencies of K0218, K00941, and K00788 compared with goat and sheep, but a higher frequency of K12153 (Fig. 5a). Since K00941, K00788, and K12153 belong to the same multi-gene node, these vitamin-synthesizing microbes maintained functional capacity through compensatory selection preferences (Fig. 5a). This pattern contrasted with the human gut microbiome, where K12153 was not utilized but K00941 and K00788 were frequently employed (Fig. 5a). These findings indicate that ruminant GIT vitamin-synthesizing microbes exhibit host-specific selection preferences in thiamine and riboflavin pathways. Nevertheless, they overall maintain consistent patterns of functional gene usage rather than indiscriminately expanding all alternative genes at multi-gene nodes. Within the multi-gene co-selection patterns of vitamin biosynthetic pathways, vitamin-synthesizing microbes among the 8 ruminant hosts showed remarkable consistency (Fig. 6a). Despite differences in the frequency of individual gene usage within multi-gene nodes, the thiamine, riboflavin, niacin, pantothenate, and folate pathways were characterized by the preferential selection of core multi-gene nodes without further expansion to redundant genes (Fig. 6a). By contrast, the pyridoxine and cobalamin pathways consistently involved multi-gene co-selection to enhance node functionality (Fig. 6a). Importantly, the relative abundance of microbes following these core co-selection patterns remained at similar levels across ruminant hosts, which highlights the functional stability of vitamin-synthesizing microbes despite variations in GIT microbial diversity.

In summary, by comprehensively reconstructing the ruminant GIT microbiome reference genome catalog CRMC together with species-specific catalogs from 8 ruminant hosts, we provided an integrated framework that reveals both the compositional structure and functional distribution patterns of ruminant GIT microbiomes. This resource not only lays the foundation for strain-level investigations of ruminant-associated microbes but also offers critical insights for elucidating microbial contributions to digestion and vitamin synthesizing.

## Conclusions

In this study, we reconstructed the comprehensive ruminant GIT microbiome reference genome catalog CRMC, together with 8 host-specific vitamin genome catalogs, thereby effectively expanding the reference range of ruminant GIT microbiomes. Furthermore, we elucidated the core microbial taxa and functional features across ruminants, as well as the compositional diversity, distribution patterns, biosynthetic pathway characteristics, and functional stability of vitamin-synthesizing microbes.

## Availability of source code and requirements

Project name: CRMC

Project homepage: https://github.com/fengtong-bio/CRMC

License: MIT License

Operating system: Linux

Programming language: Shell, Perl, R

Package management: Conda

Hardware requirements: High-performance computing nodes with ≥128 cores and ≥1 TB RAM

## Supplementary Material

giag016_Supplemental_Material

giag016_Authors_Response_To_Reviewer_Comments_original_submission

giag016_GIGA-D-25-00528_original_submission

giag016_GIGA-D-25-00528_Revision_1

giag016_Reviewer_1_Report_original_submission Reviewer 1 -- 1/11/2026

giag016_Reviewer_1_Report_revision_1 Reviewer 1 -- 1/30/2026

giag016_Reviewer_2_Report_original_submission Reviewer 2 -- 1/15/2026

giag016_Reviewer_2_Report_revision_1 Reviewer 2 -- 2/2/2026

## Data Availability

The ruminant GIT microbiome reference genome catalog CRMC used in this study are available in the Figshare database under accession code 30580403 (CRMC strain level catalog part 1) [75], 30580667 (CRMC strain level catalog part 2, CRMC species level catalog and CRMC-sheep strain level catalog) [76] and 30580790 (CRMC-buffalo, CRMC-cattle, CRMC-goat, CRMC-yak, CRMC-moose, CRMC-water deer and CRMC-roe deer strain level catalog) [77].

## Additional files

**Supplementary Figure S1:** Methodological overview of our metagenomic analysis workflow.

**Supplementary Figure S2:** Proportion of MAGs phylum distribution in the reference genome of gastrointestinal microbiota in 8 ruminant hosts.

**Supplementary Figure S3:** Annotation ratio of MAGs at different taxonomic annotation levels for reference genomes of gastrointestinal microbiota in 8 ruminant hosts.

**Supplementary Figure S4:** Distribution of MAGs Shared/Uniq between 8 ruminant hosts and other ruminants in different phyla proportions.

**Supplementary Figure S5:** Distribution of MAGs Shared/Uniq between 8 ruminant hosts and other ruminants in different functional substrate proportions.

**Supplementary Figure S6:** Relative abundance distribution of core vitamin-synthesis pathway bacteria in the gastrointestinal tract of 8 ruminant hosts.

**Supplementary Figure S7:** Relative abundance distribution of core vitamin-synthesis pathway bacteria in the gastrointestinal tract of 8 ruminant hosts in stomach.

**Supplementary Figure S8:** Relative abundance distribution of core vitamin-synthesis pathway bacteria in the gastrointestinal tract of 8 ruminant hosts in small intestine.

**Supplementary Figure S9:** Relative abundance distribution of core vitamin-synthesis pathway bacteria in the gastrointestinal tract of 8 ruminant hosts in large intestine.

**Supplementary Figure S10:** Distribution of relative abundance of MAGs synthesized from different vitamins in the gastrointestinal tract of 8 ruminant hosts.

**Supplementary Figure S11:** Relative abundance distribution of MAGs in core node patterns of different vitamin synthesis bacteria in the gastrointestinal tract of 8 ruminant hosts.

**Supplementary Table S1:** Sampling information for 2325 samples.

**Supplementary Table S2:** Number of MAGs at different genera in CRMC.

**Supplementary Table S3:** Number of MAGs at different genera in buffalo.

**Supplementary Table S4:** Number of MAGs at different genera in cattle.

**Supplementary Table S5:** Number of MAGs at different genera in goat.

**Supplementary Table S6:** Number of MAGs at different genera in moose.

**Supplementary Table S7:** Number of MAGs at different genera in roe deer.

**Supplementary Table S8:** Number of MAGs at different genera in sheep.

**Supplementary Table S9:** Number of MAGs at different genera in water deer.

**Supplementary Table S10:** Number of MAGs at different genera in yak.

**Supplementary Table S11:** Proportion of Firmicutes-A MAGs/Bacteroidota MAGs in different ruminant animals.

**Supplementary Table S12:** 2325 clean data reads mapping rate (%).

**Supplementary Table S13:** Proportion of MAGs shared among ruminants.

**Supplementary Table S14:** KEGG vitamin biosynthesis pathways.

**Supplementary Table S15:** Number of MAGs in each vitamin-synthesis map.

**Supplementary Table S16:** Frequency of node usage in vitamin-synthesis map VB1 (M00127).

**Supplementary Table S17:** Frequency of node usage in vitamin-synthesis map VB2 (M00125).

**Supplementary Table S18:** Frequency of node usage in vitamin-synthesis map VB3 (M00115).

**Supplementary Table S19:** Frequency of node usage in vitamin-synthesis map VB5 (M00119).

**Supplementary Table S20:** Frequency of node usage in vitamin-synthesis map VB6 (M00916).

**Supplementary Table S21:** Frequency of node usage in vitamin-synthesis map VB7 (M00123).

**Supplementary Table S22:** Frequency of node usage in vitamin-synthesis map VB9 (M00126).

**Supplementary Table S23:** Frequency of node usage in vitamin-synthesis map VB12 (M00122).

**Supplementary Table S24:** Frequency of node usage in vitamin-synthesis map VK2 (M00930).
