# Supplementary material for: A comprehensive ruminant microbial catalog (CRMC) reveals convergent selection for key vitamin-synthesizing pathways and genes across ruminants and human
Source: Gigascience. 2026 Feb 25;15:giag016. doi: 10.1093/gigascience/giag016 (PMC13108260; doi:10.1093/gigascience/giag016)
Supplement: giag016_Supplemental_Material [file giag016_supplemental_material.zip › Feng_etal_Ruminant_CRMC_vitamin_Supplement_Figure Ver 1.1.pdf]

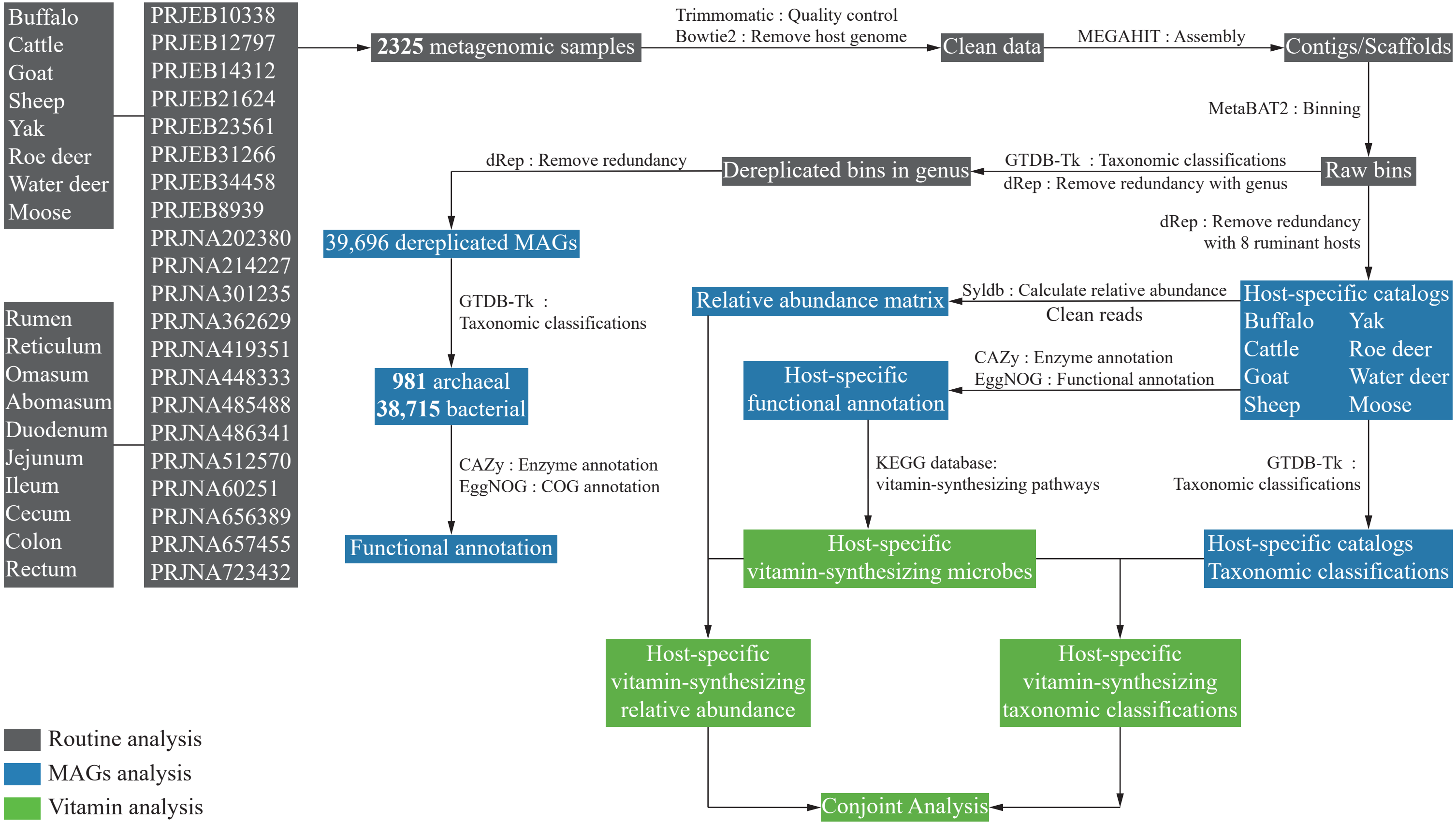

Supplementary Fig. 1 Methodological overview of our metagenomic analysis workflow.

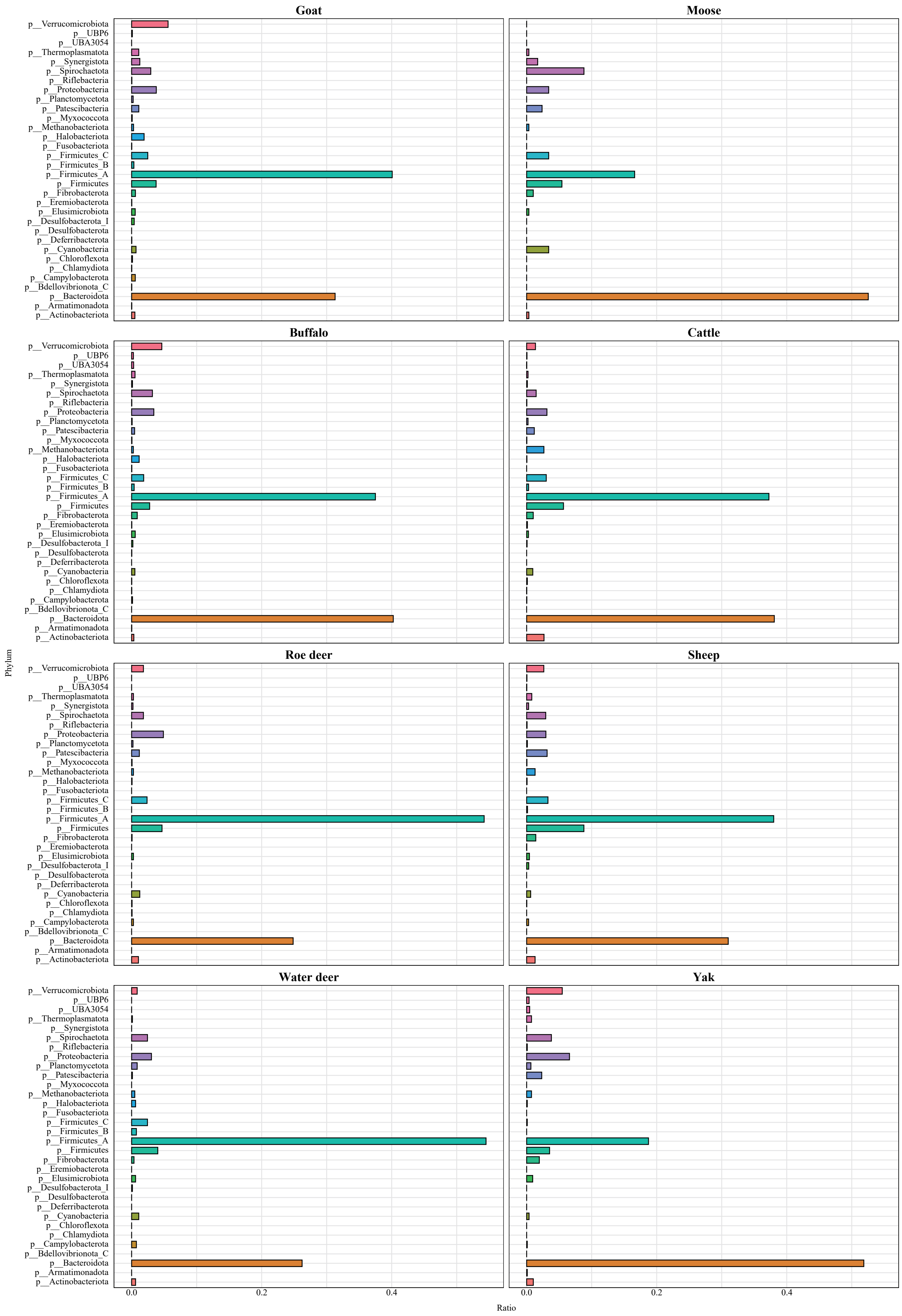

Supplementary Fig. 2 Proportion of MAGs phylum distribution in the reference genome of gastrointestinal microbiota in 8 ruminant hosts.

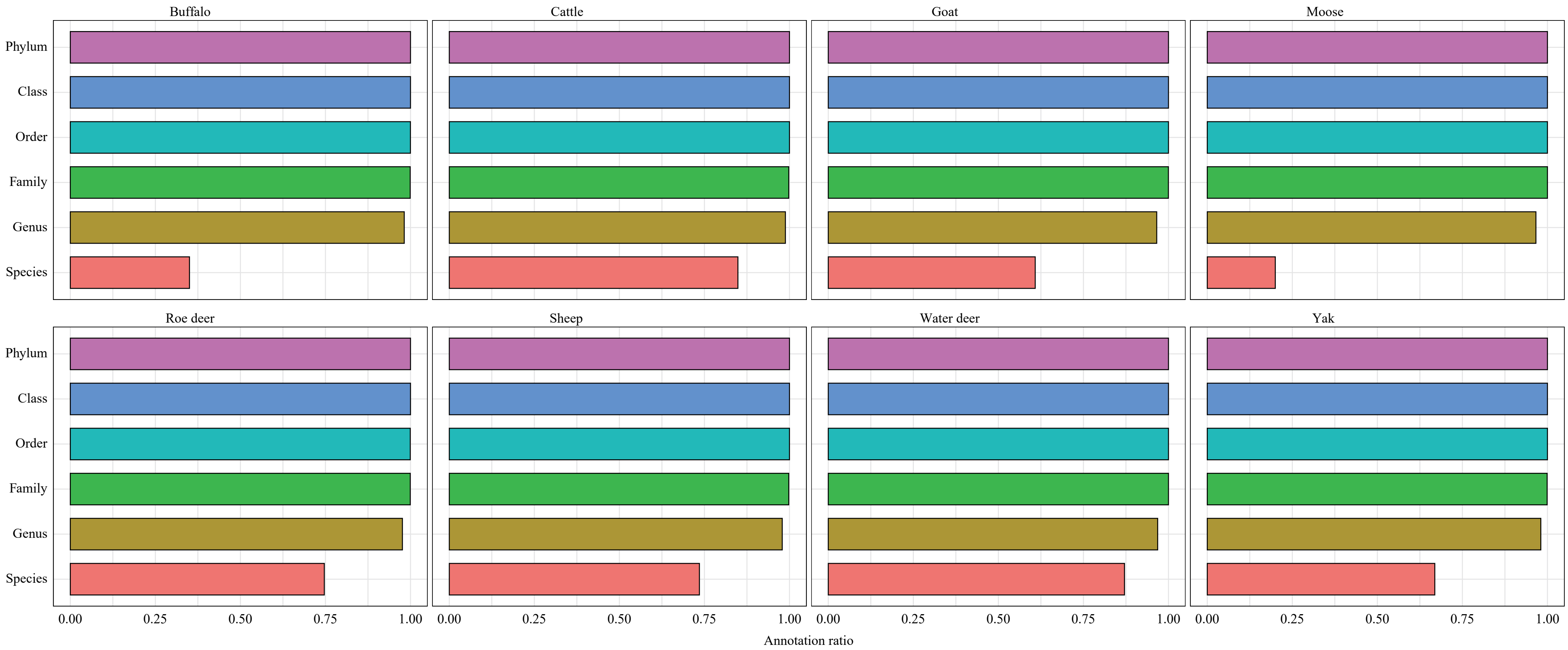

Supplementary Fig. 3 Annotation ratio of MAGs at different taxonomic annotation levels for reference genomes of gastrointestinal microbiota in 8 ruminant hosts.

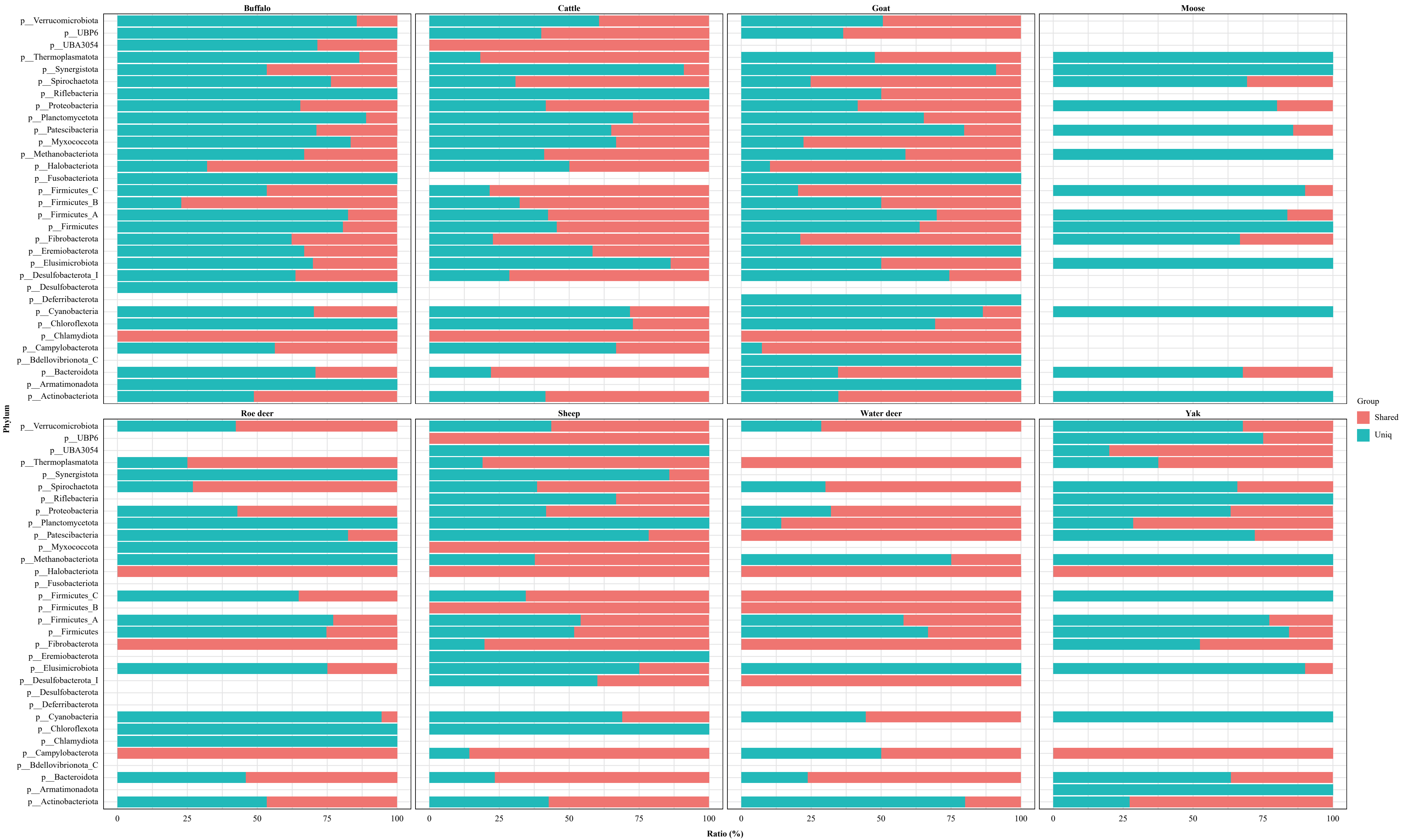

Supplementary Fig. 4 Distribution of MAGs Shared/Uniq between 8 ruminant hosts and other ruminants in different phyla proportions.

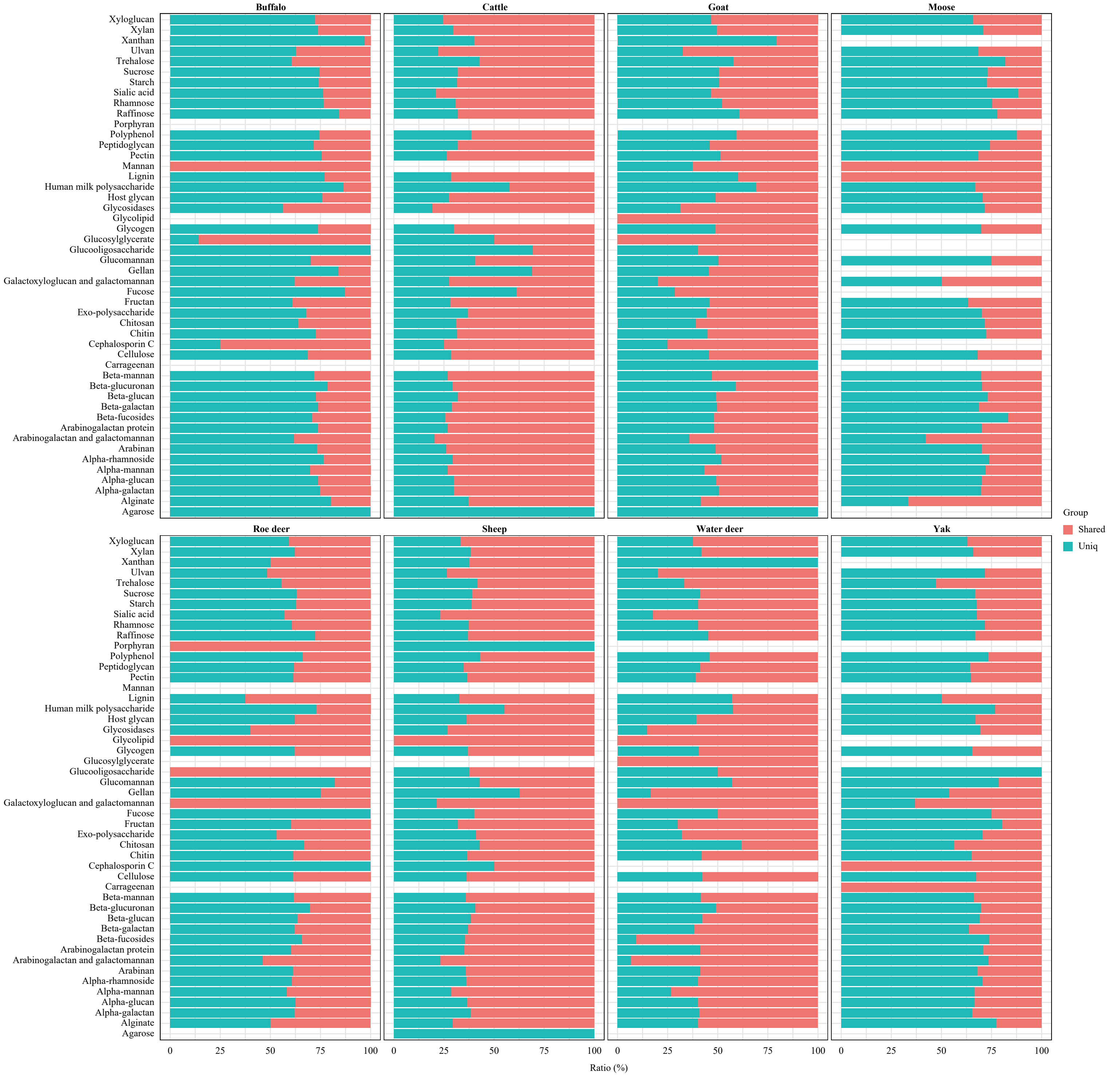

Supplementary Fig. 5 Distribution of MAGs Shared/Uniq between 8 ruminant hosts and other ruminants in different functional substrate proportions.

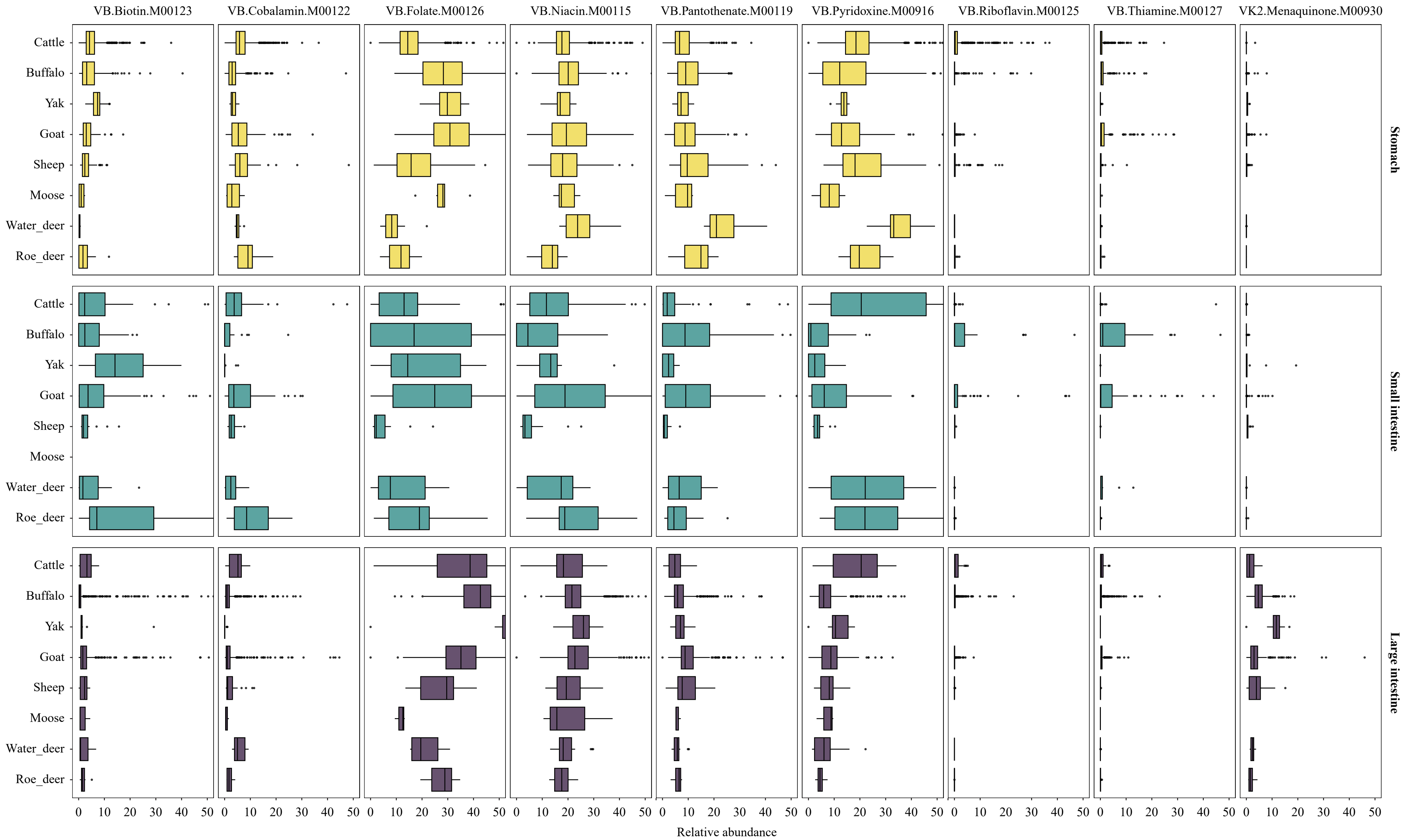

Supplementary Fig. 6 Relative abundance distribution of core vitamin-synthesis pathway bacteria in the gastrointestinal tract of 8 ruminant hosts.

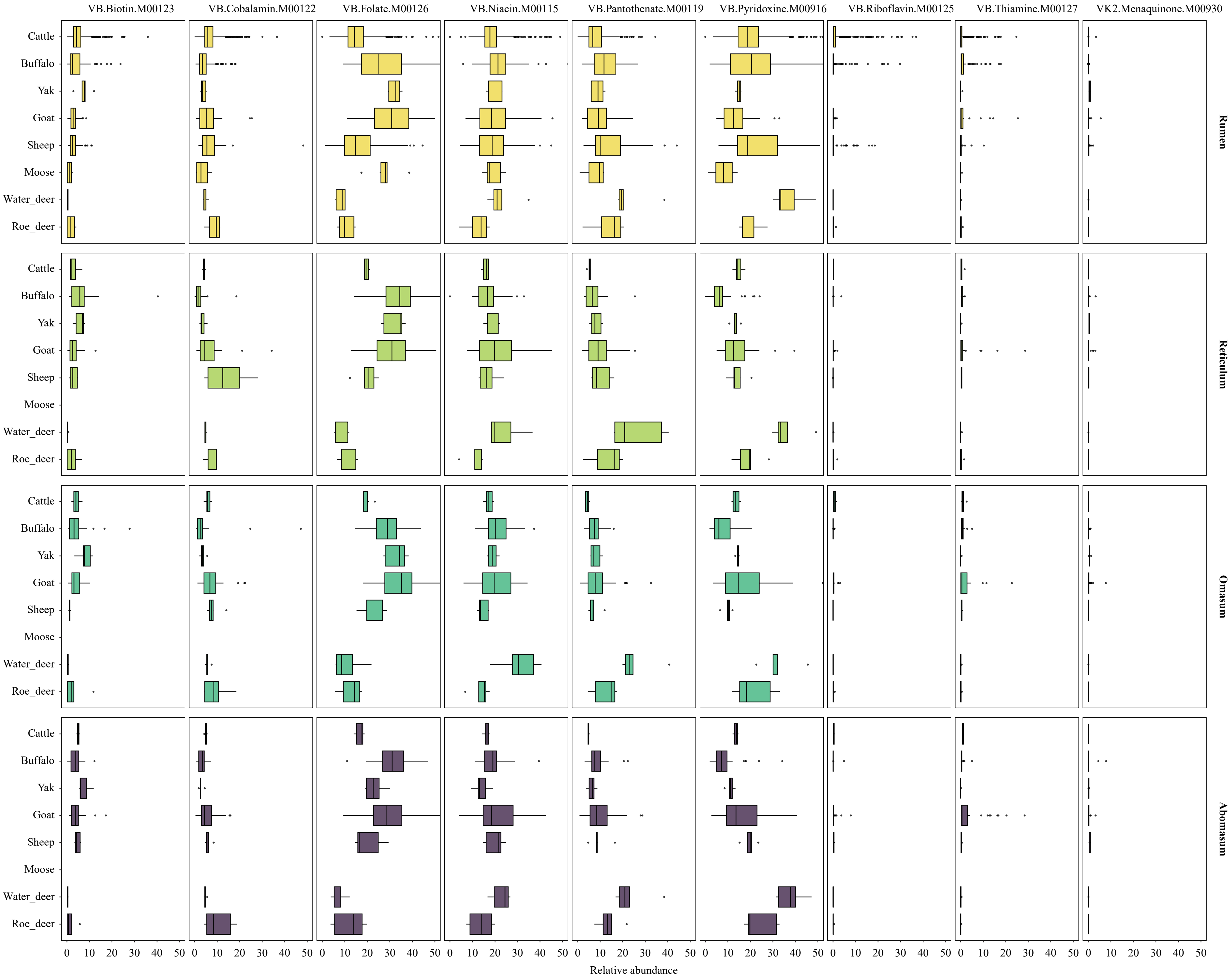

Supplementary Fig. 7 Relative abundance distribution of core vitamin-synthesis pathway bacteria in the gastrointestinal tract of 8 ruminant hosts in stomach.

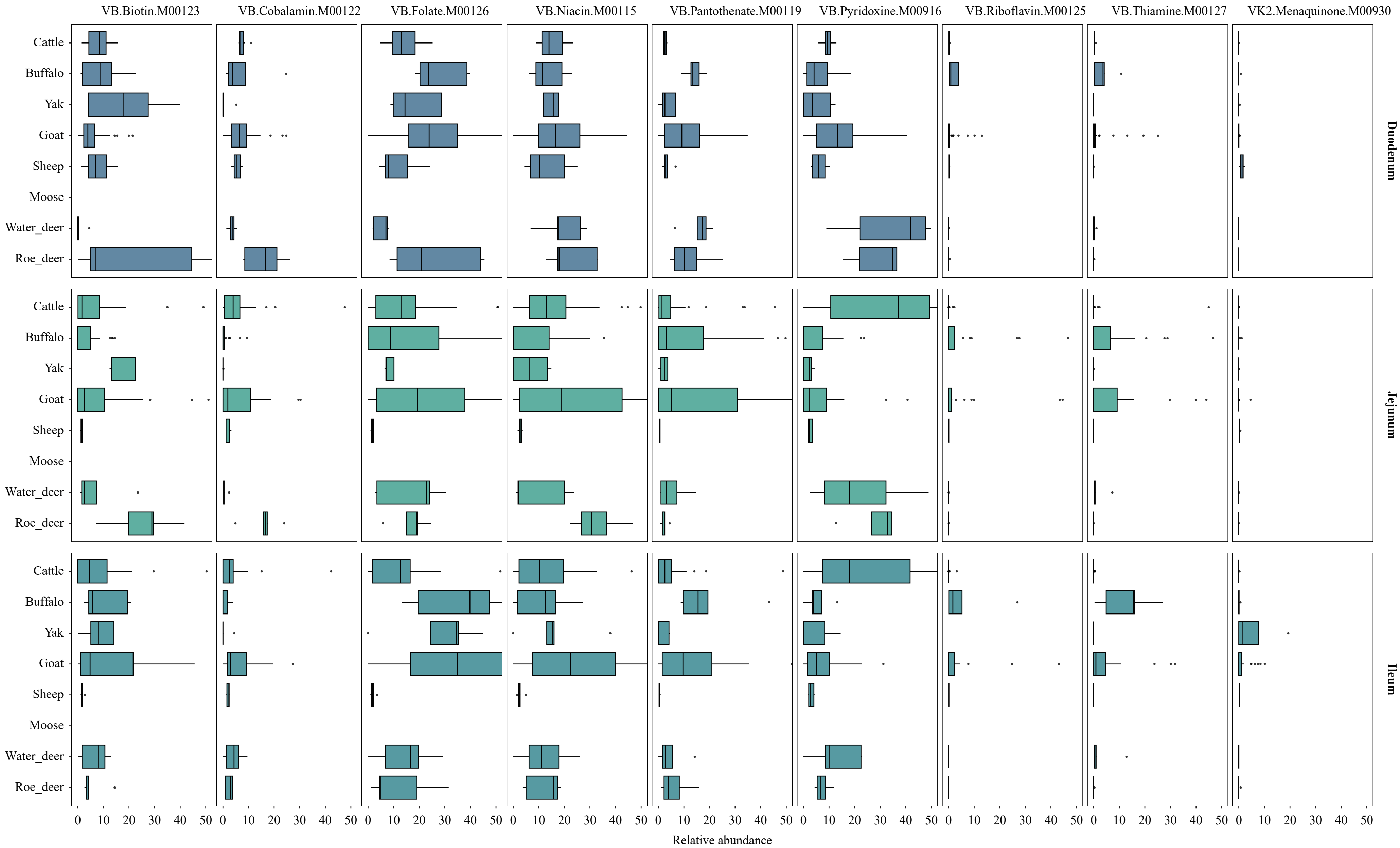

Supplementary Fig. 8 Relative abundance distribution of core vitamin-synthesis pathway bacteria in the gastrointestinal tract of 8 ruminant hosts in small intestine.

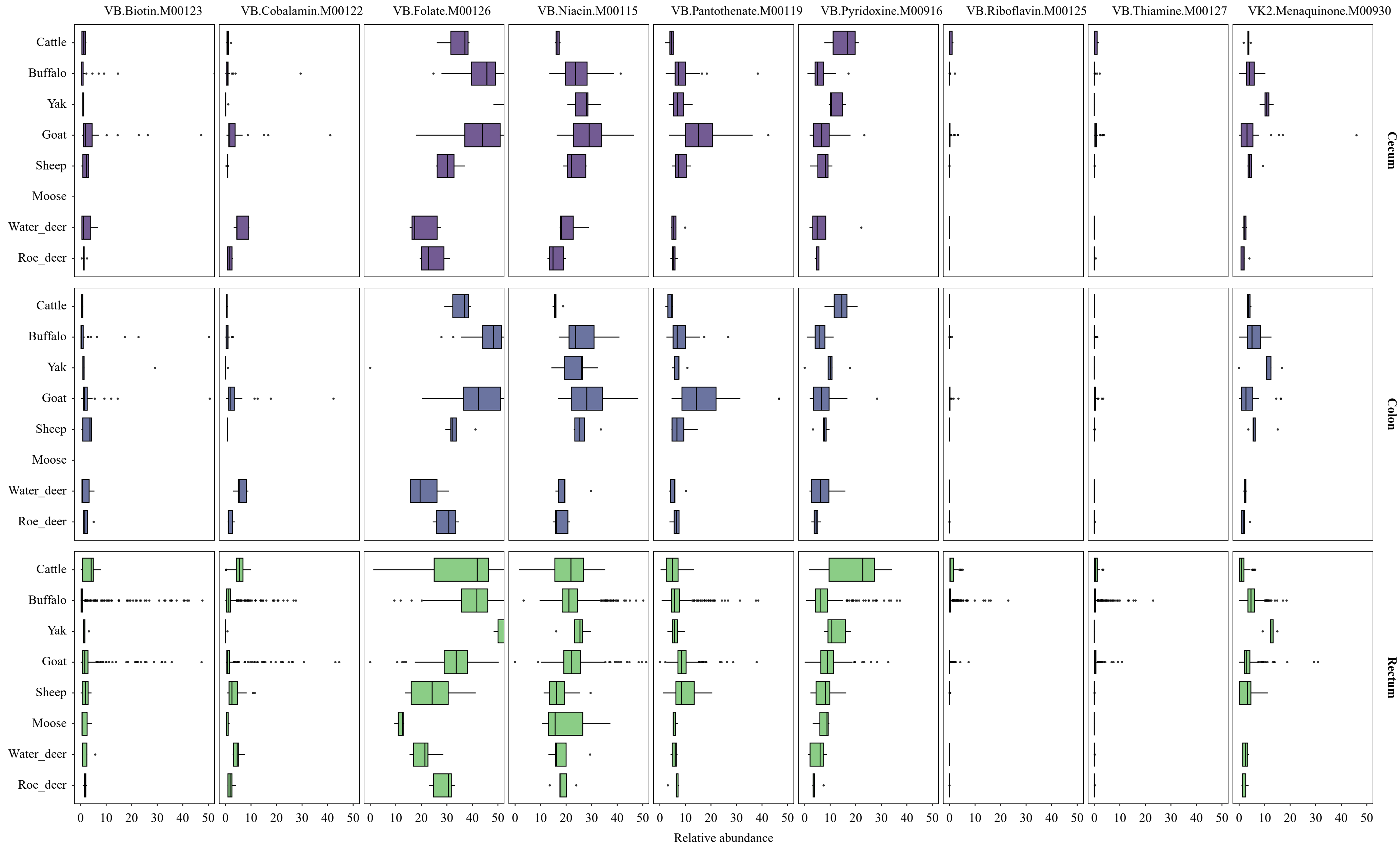

Supplementary Fig. 9 Relative abundance distribution of core vitamin-synthesis pathway bacteria in the gastrointestinal tract of 8 ruminant hosts in large intestine.

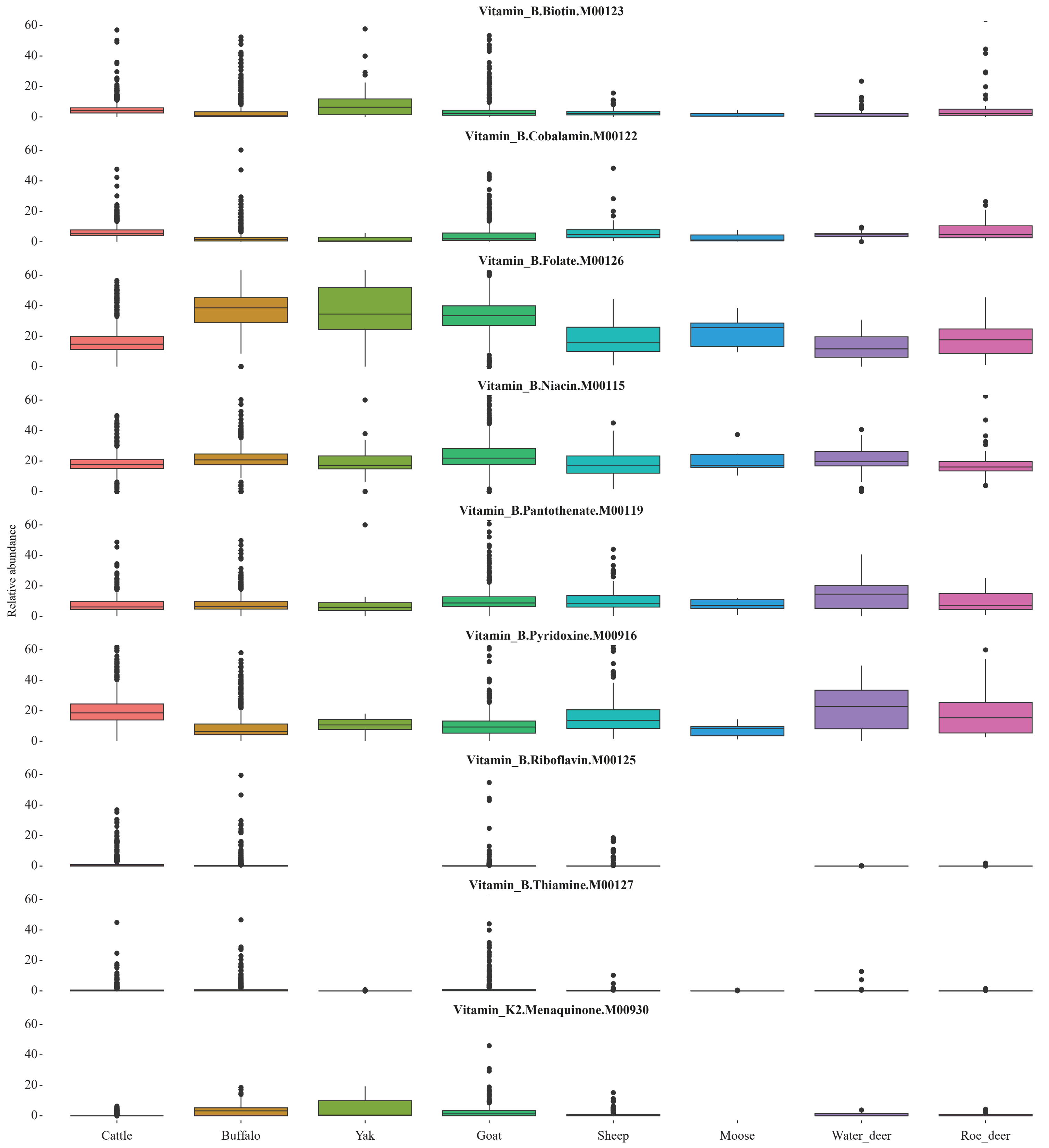

Supplementary Fig. 10 Distribution of relative abundance of MAGs synthesized from different vitamins in the gastrointestinal tract of 8 ruminant hosts.

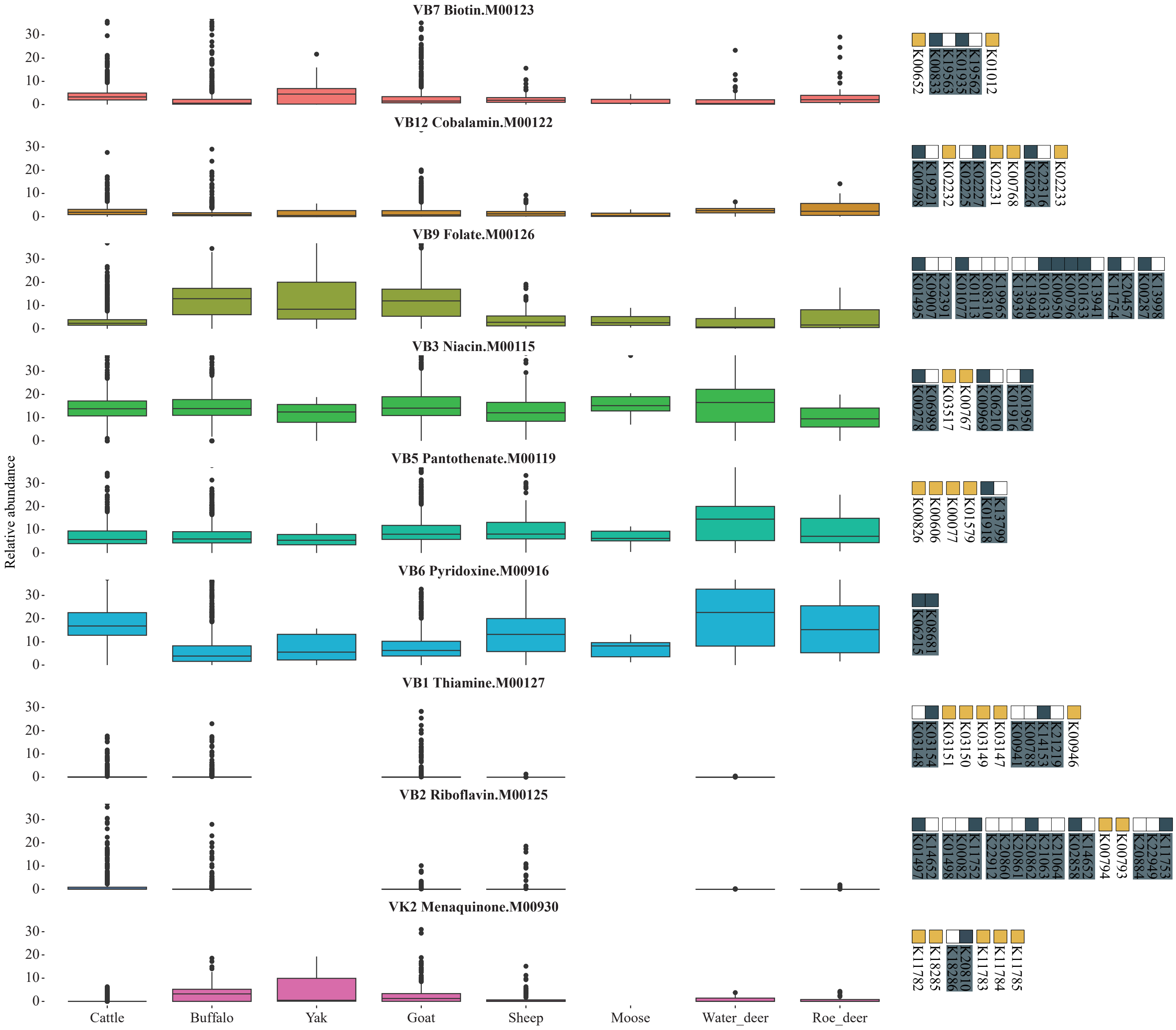

Supplementary Fig. 11 Relative abundance distribution of MAGs in core node patterns of different vitamin synthesis bacteria in the gastrointestinal tract of 8 ruminant hosts.
