# Supplementary material for: A comprehensive ruminant microbial catalog (CRMC) reveals convergent selection for key vitamin-synthesizing pathways and genes across ruminants and human
Source: Gigascience. 2026 Feb 25;15:giag016. doi: 10.1093/gigascience/giag016 (PMC13108260; doi:10.1093/gigascience/giag016)
Supplement: giag016_GIGA-D-25-00528_Revision_1 [file giag016_giga-d-25-00528_revision_1.pdf]

GigaScience

A comprehensive ruminant microbial catalog (CRMC) reveals convergent selection for key vitamin-synthesizing pathways and genes across ruminants and human

--Manuscript Draft--

**Manuscript Number:**	GIGA-D-25-00528R1
**Full Title:**	A comprehensive ruminant microbial catalog (CRMC) reveals convergent selection for key vitamin-synthesizing pathways and genes across ruminants and human
**Article Type:**	Research
**Funding Information:**	Double First-Class Construction Funds for Provincial Departments Affiliated Universities in Hubei Province (5001170159) — Prof Wei-Hua Chen
**Abstract:**	Background: The ruminant gastrointestinal tract (GIT) serves as a natural microbial reservoir in which vitamin-synthesizing microbes play key integrated roles in digestion, nutrient absorption, and metabolic balance; however, studies systematically elucidating their functional characteristics and ecological roles remain limited due to the lack of a large-scale reference genome catalog for ruminant gastrointestinal vitamin-synthesizing microbes. Here, based on 2,325 metagenomic samples from 8 ruminant hosts, we comprehensively reconstructed and analyzed the ruminant GIT microbiome and the distribution patterns of vitamin-synthesizing microbes.<br>Results: We reconstructed a unified ruminant gastrointestinal microbiome catalog (CRMC) with 39,696 MAGs, achieving the highest mapping rate (~83.45%) among 2,325 metagenomic datasets, surpassing GTDB, RGMGC, and other catalogs. Across the 8 ruminant hosts, we identified a total of 17,349 vitamin-synthesizing microbes spanning 9 biosynthetic pathways (thiamine, riboflavin, niacin, pantothenate, pyridoxine, biotin, folate, cobalamin, and menaquinone). These microbes exhibited unified pathway selection patterns consistent with those in the human gut microbiome. Furthermore, within the major vitamin-synthesizing pathways commonly selected across ruminants, vitamin-synthesizing microbes displayed concentrated co-selection of specific functional gene nodes, revealing that despite taxonomic differences among gastrointestinal vitamin-synthesizing communities, they share highly convergent pathway preferences and common node-level selection patterns.<br>Conclusions: Together, by reconstructing the ruminant GIT microbiome reference genome catalog (CRMC), we elucidated the core microbial taxa and their functional features across ruminants, as well as the pathway preferences and distribution patterns of vitamin-synthesizing microbes. These findings provide an effective reference for advancing ruminant GIT microbiome research, offering gene co-selection insights for microbial synthetic biology design, and guiding microbiome-based interventions in ruminant systems.
**Corresponding Author:**	Wei-Hua Chen<br>Huazhong University of Science and Technology<br>Wuhan, CHINA
**Corresponding Author Secondary Information:**	
**Corresponding Author's Institution:**	Huazhong University of Science and Technology
**Corresponding Author's Secondary Institution:**	
**First Author:**	Tong Feng
**First Author Secondary Information:**	
**Order of Authors:**	Tong Feng
	Yingjian Wu
	Yixue Xu
	Wei-Hua Chen

Powered by Editorial Manager® and ProduXion Manager® from Aries Systems Corporation

Order of Authors Secondary Information:	
Response to Reviewers:	Dear Dr. Dongni Ma,<br>We would like to extend our sincere appreciation for your dedicated efforts throughout our manuscript entitled “A comprehensive ruminant microbial catalog (CRMC) reveals convergent selection for key vitamin-synthesizing pathways and genes across ruminants and human”. Your guidance and support have been instrumental in helping us refines our work. We have carefully addressed the reviewer's suggestions and believe our revisions now offer a clear and comprehensive presentation of our research.<br>Thank you for your valuable support in enhancing the quality of our work. To facilitate your review of our revisions, the following is a point-by-point response to the questions and comments.<br><br>Sincerely yours,<br>Wei-Hua Chen (on behalf of all authors)<br><br><br>Response to referee 1<br>NOTE: for convenience purposes, we broke the reviewers’ comments into relevant sections, numbered them, and provided point-to-point responses. We did not truncate nor rephrase the comments by any means.<br>General remark<br>In this manuscript, the authors constructed a comprehensive ruminant microbial reference catalog (CRMC) based on 2,325 metagenomic samples across diverse ruminant hosts. The study systematically compares shared and unique microbial taxa and functions among different ruminants and highlights an interesting convergence in vitamin-synthesizing functional characteristics between the ruminant and human gut microbiomes. Overall, the study holds certain significance and provides a valuable resource for the field. The figures and the main text are well-organized.<br>Response:<br>Thank you for your positive feedback for our manuscript. We greatly appreciate your thorough review and the specific comments you provided. Your thoughtful feedback and suggestions have significantly contributed to the refinement and quality enhancement of this manuscript. We have carefully reviewed the attached document and will now address each comment in detail.<br><br>Comments for the Author<br>Comment #1<br>This study is based on second-generation metagenomics, the discussion should discuss the potential enhancements in MAG completeness and functional gene retrieval offered by third-generation sequencing in future studies.<br>Response:<br>Thank you for your suggestion. We have revised the manuscript to acknowledge the limitations of using only second-generation sequencing and to highlight the potential of third-generation sequencing for improving data quality in future work. The revised text now reads: "Although this study did not involve third-generation sequencing data, resulting in fewer MAGs reaching complete status, the CRMC not only nearly … for future studies. In future studies, the incorporation of third-generation sequencing data will further enhance the CRMC, yielding more complete-level MAGs." (Lines 588 - 596).<br>Action:<br>Corrected. You can find the revised text at line 588 - 596.<br><br>Comment #2<br>Although Archaea constitute only 2.47% of the MAGs, they are critical for rumen function (e.g., methanogenesis). Need to list the major archaeal families and genera annotated in the CRMC.<br>Response:<br>Thank you for your suggestion. We have updated the Results section to include the taxonomic composition of the archaeal MAGs. The revised text now reads: "The archaeal MAGs mainly belong to three phyla: Halobacteriota (predominantly family Methanocorpusculaceae and genus Methanocorpusculum), Methanobacteriota (predominantly family Methanobacteriaceae and genus Methanobrevibacter), and Thermoplasmatota (predominantly family Methanomethylophilaceae and genus

Powered by Editorial Manager® and ProduXion Manager® from Aries Systems Corporation

UBA71)." (Lines 272 - 276).
Action:
Corrected. You can find the revised text at line 272 - 276.

Comment #3
Regarding the identification of vitamin-synthesizing microbes, the criteria need clarification. Did it require complete pathways on a single contig or within a MAG?
Response:
Thank you for your suggestion. We have clarified the identification criteria in the Methods section to specify that the presence of all pathway genes within a single MAG is enough. The revised text now reads: "1.Only MAGs encoding the full complement of genes for an entire vitamin biosynthesis pathway were considered as vitamin-synthesizing candidates. Specifically, the identification criteria required the presence of all pathway genes within a single MAG, but not necessarily on a single contig." (Lines 196 - 198).
Action:
Corrected. You can find the revised text at line 196 - 198.

Comment #4
The discussion needs to speculate on the underlying reasons for the observed functional convergence between ruminant and human gut microbiomes.
Response:
Thank you for your suggestion. We have added a detailed explanation in the Discussion section regarding the consistent vitamin synthesis pathways observed in both ruminant and human guts. The revised text now reads: "We speculate that this functional convergence is driven by shared environmental selective pressures within the gut, such as stable temperature, anaerobic conditions, and consistent physicochemical properties. Consequently, the vitamin-synthesizing bacteria that stably colonize these gut environments hold significant potential for identification and application as next-generation probiotics." (Lines 632 - 636).
Action:
Corrected. You can find the revised text at line 632 - 636.

Comment #5
To ensure project reproducibility, the open-source link to the workflow needs to be added in the methods section.
Response:
Thank you for your suggestion. We have added the open-source link to our workflow in the methods section. The revised text now reads: "The software commands and scripts used in this study are available on GitHub (https://github.com/fengtong-bio/CRMC)." (Lines 244 - 245).
Action:
Corrected. You can find the revised text at line 244 - 245.

Response to referee 2
NOTE: for convenience purposes, we broke the reviewers' comments into relevant sections, numbered them, and provided point-to-point responses. We did not truncate nor rephrase the comments by any means.
General remark
The unique gastrointestinal tract structure of ruminants serves as a natural microbial reservoir, playing a vital role in digestion, absorption, and nutritional balance. In this study, the authors constructed a comprehensive ruminant microbial reference catalog (CRMC) using 2,325 metagenomic samples and summarized the functional characteristics of vitamin-synthesizing microbes in ruminants. And a meaningful ruminant gastrointestinal tract microbiome reference genome catalog (CRMC) was constructed, which included 39,696 l MAGs. A total of 17,349 vitamin-synthesizing microbes spanning 9 biosynthetic pathways (thiamine, riboflavin, niacin, pantothenate, pyridoxine, biotin, folate, cobalamin, and menaquinone) were identified. This paper will be of interest to scientists who study the rumen function of herbivorous animals, including cattle and sheep, and is particularly meaningful for research on precision nutrition in the rumen. Overall, this study holds certain significance as a valuable resource for the ruminant microbial field. I agree to be published after minor revision.
Response:

	Thank you for your positive assessment and for the opportunity to revise our manuscript. We greatly appreciate your detailed review and the constructive spirit of your comments. Your suggestions have been exceptionally helpful in guiding us to strengthen the arguments presented in our paper and to improve the presentation of our data. We have treated your feedback with the utmost seriousness and have conducted a comprehensive revision of the manuscript. In the following section, we provide a detailed account of how we have addressed each of your specific comments and incorporated your suggestions into the final version.<br><br>Comments for the Author<br>Comment #1<br>The terms "gastrointestinal tract" and "GIT" are used interchangeably; "GIT" needs to be used consistently throughout the text after the first definition.<br>Response:<br>Thank you for your suggestion. We have checked the full text and ensured that "GIT" is used consistently throughout the manuscript after its first mention.<br><br>Comment #2<br>In Figure 1f, the red portion needs to be explicitly labeled as "CRMC Unique" in the legend to prevent ambiguity.<br>Response:<br>Thank you for your suggestion. We have updated the legend in Figure 1f from "Unique" to "CRMC Unique" to ensure clarity and accuracy.<br><br>Comment #3<br>In Figure 3a, the figure legend needs to confirm that the color coding for phyla is consistent between the human and ruminant datasets.<br>Response:<br>Thank you for your suggestion. We have updated the legend for Figure 3a to explicitly confirm that the color scheme is consistent across datasets. The revised legend now reads: " Each color represents a different phylum, and bar length indicates the number of MAGs. The color coding for phyla is consistent between the ruminant and human datasets." (Lines 460 - 461).<br>Action:<br>Corrected. You can find the revised text at line 460 - 461.<br><br>Comment #4<br>The Methods section needs to clarify whether the screening criteria for identifying human gut vitamin-synthesizing microbes were consistent with the standards used for the CRMC.<br>Response:<br>Thank you for your suggestion. We have clarified in the Methods section that the human dataset was processed using the exact same criteria to ensure valid comparisons. The revised legend now reads: " Candidate vitamin-synthesizing MAGs were defined according to the following criteria (the same screening workflow was applied to the human IMGG dataset):" (Lines 193 - 194).<br>Action:<br>Corrected. You can find the revised text at line 193 - 194.<br><br>Comment #5<br>Open-source links for the software and code used in the Methods section need to be provided.<br>Response:<br>Thank you for your suggestion. We have added the open-source link to our workflow in the methods section. The revised text now reads: "The software commands and scripts used in this study are available on GitHub (https://github.com/fengtong-bio/CRMC)." (Lines 244 - 245).<br>Action:<br>Corrected. You can find the revised text at line 244 - 245.
**Additional Information:**	
**Question**	**Response**
Are you submitting this manuscript to a	No

Powered by Editorial Manager® and ProduXion Manager® from Aries Systems Corporation

special series or article collection?	
**Experimental design and statistics**<br><br>Full details of the experimental design and statistical methods used should be given in the Methods section, as detailed in our Minimum Standards Reporting Checklist. Information essential to interpreting the data presented should be made available in the figure legends.<br><br>Have you included all the information requested in your manuscript?	Yes
**Resources**<br><br>A description of all resources used, including antibodies, cell lines, animals and software tools, with enough information to allow them to be uniquely identified, should be included in the Methods section. Authors are strongly encouraged to cite Research Resource Identifiers (RRIDs) for antibodies, model organisms and tools, where possible.<br><br>Have you included the information requested as detailed in our Minimum Standards Reporting Checklist?	Yes
**Availability of data and materials**<br><br>All datasets and code on which the conclusions of the paper rely must be either included in your submission or deposited in publicly available repositories (where available and ethically appropriate), referencing such data using a unique identifier in the references and in the "Availability of Data and Materials" section of your manuscript.<br><br>Have you have met the above requirement as detailed in our Minimum Standards Reporting Checklist?	Yes

Powered by Editorial Manager® and ProduXion Manager® from Aries Systems Corporation

GigaScience has policies and guidelines in place for the use of generative AI-writing tools such as ChatGPT. If you have used such writing tools to assist with writing the manuscript this must be declared and cited in the text. Authors should not list AI-writing tools and other AI-assisted technologies as an author or co-author and should acknowledge that they are fully responsible for text generated or refined by AI-writing tools.<p><br><br>A summary of use (particularly in the introduction or among methods) needs to be included at the end of the paper, and the outputs should also be included as a supplementary file hosted in GigaDB or other open repositories. Please read our guidelines for more information. <p><br><br>By submitting to GigaScience, you are aware of the journal's AI-writing tools policy, and if you have declared use of such tools below, you have acknowledged this where appropriate in your manuscript and have made a summary of use and outputs available. <p><br><b>AI-assisted writing tools have been used in the preparation of this manuscript?	No

Manuscript

Click here to access/download;Manuscript;Feng_etal_Ruminant_CRMC_vita

A comprehensive ruminant microbial catalog (CRMC) reveals convergent selection for key vitamin-synthesizing pathways and genes across ruminants and human

Tong Feng[1*], Yingjian Wu[1*], Yixue Xu[2*], Wei-Hua Chen[1,3†]

[1] Key Laboratory of Molecular Biophysics of the Ministry of Education, Hubei Key Laboratory of Bioinformatics and Molecular-imaging, Center for Artificial Biology, Department of Bioinformatics and Systems Biology, College of Life Science and Technology, Huazhong University of Science and Technology, Wuhan 430074, Hubei, China

[2] State Key Laboratory for Conservation and Utilization of Subtropical Agro-Bioresources, Guangxi University, Nanning 530005, China

[3] School of Biological Science, Jining Medical University, Rizhao, 272111, China

* Contributed equally to this work

† Correspondence should be addressed to Wei-Hua Chen (weihuachen@hust.edu.cn)

Tong Feng (0000-0002-6056-0590), Yingjian Wu (0000-0002-9720-6712), Yixue Xu (0009-0008-0447-3880), and Wei-Hua Chen (0000-0003-2168-6147)

Abstract

Background: The ruminant gastrointestinal tract (GIT) serves as a natural microbial reservoir in which vitamin-synthesizing microbes play key integrated roles in digestion, nutrient absorption, and metabolic balance; however, studies systematically elucidating their functional characteristics and ecological roles remain limited due to the lack of a large-scale reference genome catalog for ruminant gastrointestinal vitamin-synthesizing microbes. Here, based on 2,325 metagenomic samples from 8 ruminant hosts, we comprehensively reconstructed and analyzed the ruminant GIT microbiome and the distribution patterns of vitamin-synthesizing microbes.

Results: We reconstructed a unified ruminant gastrointestinal microbiome catalog (CRMC) with 39,696 MAGs, achieving the highest mapping rate (~83.45%) among 2,325 metagenomic datasets, surpassing GTDB, RGMGC, and other catalogs. Across the 8 ruminant hosts, we identified a total of 17,349 vitamin-synthesizing microbes spanning 9 biosynthetic pathways (thiamine, riboflavin, niacin, pantothenate, pyridoxine, biotin, folate, cobalamin, and menaquinone). These microbes exhibited unified pathway selection patterns consistent with those in the human gut microbiome. Furthermore, within the major vitamin-synthesizing pathways commonly selected across ruminants, vitamin-synthesizing microbes displayed concentrated co-selection of specific functional gene nodes, revealing that despite taxonomic differences among gastrointestinal vitamin-synthesizing communities, they share highly convergent pathway preferences and common node-level selection patterns.

Conclusions: Together, by reconstructing the ruminant GIT microbiome reference genome catalog (CRMC), we elucidated the core microbial taxa and their functional features across ruminants, as well as the pathway preferences and distribution patterns of vitamin-synthesizing microbes. These

findings provide an effective reference for advancing ruminant GIT microbiome research, offering gene co-selection insights for microbial synthetic biology design, and guiding microbiome-based interventions in ruminant systems.

Keywords: Ruminant, gastrointestinal tract, microbiome, metagenome-assembled genomes, vitamin-synthesizing, cross-species comparison

Introduction

Ruminants possess a highly specialized gastrointestinal tract (GIT) system that efficiently converts low-quality forage into high-value products such as milk and meat, conferring substantial economic importance and representing one of the most intricate symbiotic systems in nature[1-5]. The ruminant GIT microorganisms include bacteria, archaea, anaerobic fungi, protozoa and viruses[4, 5]. These microorganisms cooperatively deconstruct cellulose and lignin, depolymerize plant polysaccharides, and ferment soluble substrates [6-8]. The ruminant GIT microorganisms also synthesize microbial protein, a wide spectrum of volatile fatty acids, and essential micronutrients such as B-vitamins and vitamin K_2, thereby supporting host nutritional balance and reducing the need for exogenous supplementation[9-11]. Together, the unique anatomical structure and microbial features of the ruminant GIT confer remarkable roughage tolerance, digestive capacity, and nutritional value.

Ruminants, with their exceptionally rich GIT microbial reservoirs, have inspired diverse research perspectives[3, 5-8, 12]. In our previous work, we established reference catalogs of the intestinal microbiota in buffalo and goats, and elucidated that microbial community structures in

ruminants are markedly shaped by intestinal location and dietary composition[6, 7]. Similarly, Fei et al. (2021) constructed a comprehensive ruminant microbial genome catalog from 370 metagenomic samples, comprising nearly 10,000 metagenome-assembled genomes (MAGs), which provided a foundational framework for exploring ruminant GIT microbiota [4]. Nevertheless, given the current scale of ruminant microbiome research, there remains a pressing need for reference collections with larger sample sizes and broader species coverage, encompassing independent microbial genomes from multiple ruminant hosts to enable deeper exploration of microbiome diversity and function.

Vitamins are indispensable cofactors and regulators in numerous physiological processes, including energy metabolism, immune modulation, and cellular homeostasis, and their adequate supply is essential for growth, reproduction, and health maintenance in mammals[13-17]. Increasing evidence has shown that the GIT microbiota contributes substantially to vitamin provisioning: specific bacterial taxa are capable of synthesizing B-group vitamins and vitamin K, thereby complementing dietary intake and supporting host nutritional balance[18-21]. In ruminants, rumen microbes play a pivotal role in the production of water-soluble B vitamins and vitamin K_2. Jiang et al. (2022) revealing that most microbial genomes possess only limited biosynthetic capacity and that cobalamin synthesis is particularly sensitive to dietary composition, being inhibited under high-grain diets[9]. However, a systematic study of vitamin-synthesizing bacteria based on a more comprehensive reference genome catalog of the ruminant GIT microbiome is still lacking, limiting our understanding of their ecological distribution and functional contributions.

We reconstructed a comprehensive ruminant microbial genome catalog (CRMC) based on 2,333 metagenomic samples, comprising 39,696 MAGs, together with species-specific reference

catalogs for 8 ruminant hosts (buffalo, cattle, goat, sheep, yak, roe deer, water deer and moose) generated under a unified analytical framework. This effort fills a critical gap in genomic resources and enables robust cross-species comparisons of microbial composition and functional repertoires, revealing both conserved and host-specific features. Building on these standardized catalogs, we further characterized the distribution of vitamin-synthesizing microbes, identifying consistent biosynthetic pathways and core metabolic preferences across ruminant's vitamin-synthesizing microbes and found that humans exhibited the same vitamin biosynthetic pathway preferences as observed in ruminants. Overall, the CRMC provides an updated and robust genomic foundation for ruminant GIT microbiome research, while the systematic mapping of vitamin-synthesizing taxa yields novel insights into their ecological roles and establishes fundamental genomic principles of vitamin-synthesizing microbes derived from natural ruminant GIT, thereby informing functional microbiota mining, synthetic community design, and synthetic biology applications.

Methods

Data collection, quality control, and host/food genome removal

To comprehensively reconstruct a reference genome catalog for ruminant gastrointestinal tract (GIT) microbiota, we collected 2,325 publicly available metagenomic samples from 21 NCBI projects (Supplement Table S1) [4, 6, 7, 22-37]. These samples covered 10 GIT regions (rumen, reticulum, omasum, abomasum, duodenum, jejunum, ileum, cecum, colon and rectum) from 8 representative ruminant species (buffalo, cattle, goat, sheep, yak, roe deer, water deer and moose; Figure 1a; Supplement Table S1). Raw reads were processed using Trimmomatic (v0.39) [38] with the

parameters "ILLUMINACLIP:TruSeq3-PE.fa:2:30:10 SLIDINGWINDOW:4:15 MINLEN:50 LEADING:3 TRAILING:3" to remove low-quality bases and adapter sequences. To eliminate host- and diet-associated contamination, quality-filtered reads were mapped against a set of host and food reference genomes using Bowtie2 (v2.3.5.1) [39] with the "--very-sensitive" parameter. The reference genome panel included *Capra hircus* (GCF_001704415.1) [40], *Bubalus bubalis* (GCA_004794615.1) [41], *Camelus bactrianus* (GCF_000767855.1) [42], *Camelus dromedarius* (GCF_000803125.2) [43], *Bos taurus* (GCF_002263795.1) [44], *Alces alces* (GCA_007570765.1) [45], *Cervus elaphus* (GCF_910594005.1) [46], *Rangifer tarandus caribou* (GCA_019903745.1) [47], *Capreolus capreolus* (GCA_000751575.1) [48], *Ovis aries* (GCF_016772045.1) [49], *Hydropotes inermis* (GCA_020226075.1) [50], *Bos grunniens* (GCA_005887515.2) [51], as well as ruminant food genomes including *Glycine max* (GCF_000004515.6) [52], *Zea mays* (GCF_902167145.1) [53], and *Medicago truncatula* (GCF_003473485.1) [54]. The remaining reads after decontamination were defined as clean data and used for subsequent analyses.

Assembly, binning, quality assessment, dereplication, and construction of the CRMC and species-specific catalogs

Each clean metagenomic samples was processed using identical parameters, with unspecified settings kept at their defaults (Supplementary Fig. 1):

First, assemblies were generated with MEGAHIT (v1.2.8) [55] using the parameter "--min-contig-len 1000" to retain contigs of at least 1 kb.

Second, contig binning was performed by mapping reads back to assemblies with BWA-MEM (v0.7.17) and calculating depth profiles with Samtools (v1.9) [56] and the

"jgi_summarize_bam_contig_depths" function of MetaBAT2 (v2.12.1) [57], followed by binning. Third, genome bins were subjected to quality filtering and dereplication using CheckM (v1.1.1) [58] (completeness ⩾ 50%, contamination ⩽ 10%) and dRep (v2.3.2) [59] (strain-level dereplication at ANI 99%) to construct the comprehensive ruminant microbial genome catalog (CRMC).

Due to the large number of genomes generated by the initial binning, we first classified all bins taxonomically with GTDB-Tk (v1.2.0) [60] and then applied dRep (v2.3.2) [59] dereplication at the genus level to reduce redundancy at the strain level. In parallel, we independently reconstructed species-specific microbial genome catalogs for the 8 ruminant hosts by applying the same quality filtering, and dereplication strategy as used for the CRMC.

After dereplication, the CRMC contained a total of 16,431 MAGs at the species-level and 39,696 MAGs at the strain-level. The species-specific catalogs contained 11,326 MAGs in buffalo, 10,488 in cattle, 9,745 in goat, 4,703 in sheep, 1,078 in yak, 1,433 in roe deer, 820 in water deer, and 296 in moose, respectively. These catalogs were subsequently used for cross-species comparisons of microbial composition and functional repertoires.

Taxonomic annotation, gene prediction, and functional annotation

To determine the taxonomic composition and functional potential of the CRMC and the 8 species-specific ruminant microbial genome catalogs, we applied a uniform annotation pipeline. Each MAG was taxonomically classified using the “classify_wf” workflow of GTDB-Tk (v1.2.0) [60], and phylogenetic relationships were inferred with the “infer” function. The resulting phylogenetic trees, together with annotation metadata, were visualized and edited in iTOL (v6) [61].

For functional annotation, protein-coding genes were predicted from each MAG using Prokka (v1.14.5) [62]. Predicted proteins were subsequently annotated with eggnog-mapper (v2.1.12) [63] for KEGG pathways and COG functions, run_dbcan (v4.1.0) [64] for CAZy family carbohydrate-active enzymes, barrnap (v0.9) for rRNA genes, and tRNAscan-SE (v2.0.12) [65] for tRNA structures. This combined pipeline provided comprehensive functional and structural annotations for downstream analyses of ruminant microbial genomes.

Comparative evaluation with public genome catalogs and reads mapping rate analysis

To evaluate the novelty of the CRMC, we calculated pairwise average nucleotide identity (ANI) between MAGs in the CRMC and those in existing public datasets, including GTDB-db [60], RGMGC [4], pig [66], human [67], and mouse [68] catalogs, using fastANI (v1.1) [69]. At ANI thresholds of 95% and 99%, we counted the number of MAGs shared between the CRMC and each public dataset, and further calculated the proportion of MAGs unique to the CRMC.

Moreover, we compared the read mapping rates between the CRMC and public datasets. Clean reads from 2,325 ruminant GIT metagenomic samples were aligned against the CRMC and each public catalog using BWA-MEM (v0.7.17), and mapping rates were calculated with Samtools (v1.9) [56] to provide a direct measure of the enhancement offered by the CRMC over public datasets.

Cross-species comparison of microbial composition and functions

To compare the commonalities and differences of GIT microbiomes across ruminant hosts, we analyzed the microbial community structure and functional features based on the 8 species-specific

genome catalogs. Pairwise average nucleotide identity (ANI) between MAGs in each host-specific catalog and those in the other seven hosts was calculated using fastANI (v1.1) [69], with MAGs defined as shared or host-specific at an ANI threshold of 99%.

To identify taxa that tend to be shared or specific to ruminant hosts, Fisher tests were performed based on the phylum-level taxonomic annotations of MAGs. A significance threshold of P value $\leqslant$ 0.05 was applied to determine whether specific phyla were significantly enriched in either shared or host-specific groups.

Furthermore, Fisher tests (P value $\leqslant$ 0.05) were performed based on substrate annotations of protein-coding genes from shared and host-specific MAGs to evaluate whether carbohydrate utilization patterns displayed commonalities or host-specific characteristics across ruminants.

Relative abundance estimation of MAGs

To obtain more accurate estimates of relative abundance, we calculated species-specific MAG abundances for each ruminant host using a unified pipeline. First, reference indexes were built for each catalog with the “sketch” function of Sylph (v0.6.1) [70]. Next, the “profile” function of Sylph (v0.6.1) [70] was applied to estimate the relative abundance of MAGs in each catalog. Unlike coverage-based approaches, the k-mer–based strategy implemented in Sylph enables both efficient and precise resolution of MAG relative abundances, thereby improving the accuracy of cross-sample and cross-species comparisons.

Identification and abundance estimation of vitamin-synthesizing microbes

To identify vitamin-synthesizing microbes and estimate their relative abundances in each ruminant GIT, we combined functional annotations of host-specific microbial genome catalogs with KEGG vitamin biosynthesis pathways (Table S2). Candidate vitamin-synthesizing MAGs were defined according to the following criteria (the same screening workflow was applied to the human IMGG dataset):

1. Only MAGs encoding the full complement of genes for an entire vitamin biosynthesis pathway were considered as vitamin-synthesizing candidates. Specifically, the identification criteria required the presence of all pathway genes within a single MAG, but not necessarily on a single contig.
2. For intermediate pathway nodes involving multiple genes, only MAGs containing at least one of the relevant genes were considered to encode the corresponding function.
3. Based on the relative abundance matrices of each host-specific microbial genome catalog, MAG abundances associated with the same vitamin biosynthesis pathway were summed to obtain the total relative abundance of vitamin-synthesizing microbes for that pathway.

This approach allowed us to systematically extract vitamin-synthesizing MAGs and quantify their contributions to vitamin biosynthesis across different ruminant hosts.

Regional distribution of vitamin-synthesizing microbes within ruminant gastrointestinal tracts

To further investigate the distribution of vitamin-synthesizing microbes across GIT regions in

different ruminant hosts, we partitioned the identified vitamin-synthesizing MAGs according to host species, GIT location, and vitamin biosynthesis type. The relative abundance distributions of vitamin-synthesizing microbes were then characterized across different hosts, GIT regions, and biosynthetic pathways. Relative abundance values were obtained from the standardized MAG abundance matrices described above. Visualization of distribution patterns was performed using the ggridges (v0.5.7) package in R.

Frequency analysis of biosynthetic pathway nodes in vitamin-synthesizing microbes

To further characterize the usage of multi-gene nodes within vitamin biosynthesis pathways across different ruminant hosts, we quantified the proportions of protein-coding genes in vitamin-synthesizing MAGs mapped to each pathway node. For each ruminant host and vitamin biosynthesis pathway, we calculated both (i) the number of MAGs assigned to the pathway and (ii) the proportion of MAGs containing genes corresponding to each multi-gene node.

Pathway structures were obtained from KEGG vitamin biosynthesis pathways (Table S2). Visualization of pathway node usage patterns was conducted using the pheatmap (v1.0.12) package in R.

Pattern analysis of vitamin-synthesizing microbes biosynthetic pathway node preferences across ruminants

Based on the node usage frequencies of vitamin biosynthesis pathways, we further analyzed the co-selection patterns of multi-gene pathway nodes within individual MAGs and characterized how

these patterns varied across different ruminant hosts. The analysis consisted of two steps:

1. For each vitamin-synthesizing MAG, gene information corresponding to selected pathway nodes was extracted, and MAGs carrying the same set of node genes were assigned to the same pattern category.
2. For each ruminant host and vitamin biosynthesis pathway, we calculated both the number and relative abundance of MAGs belonging to each pattern category.

Visualization of the patterns of multi-gene node usage by vitamin-synthesizing microbes across different ruminant hosts was carried out using the ggplot2 (v3.5.1) package in R.

Statistics

In addition to the software, data filtering and processing were performed using custom Perl and R scripts. Unless otherwise specified, statistical significance between groups was assessed using the Wilcoxon rank-sum test. All statistical analyses were conducted on the dataset comprising 2,325 ruminant GIT metagenomic samples. The software commands and scripts used in this study are available on GitHub (see Availability of Source Code and Requirements).

Results

Reconstruction of the Comprehensive Ruminant Microbial Catalog (CRMC)

To enable consistent functional annotation of microbial genomes of the ruminant gastrointestinal tract (GIT), we reconstructed the microbial genomes using 2,325 metagenome data from 8 ruminant

hosts (buffalo, cattle, goat, sheep, yak, roe deer, water deer, and moose; Fig 1a; Supplement Table S1) covering 10 GIT regions (rumen, reticulum, omasum, abomasum, duodenum, jejunum, ileum, cecum, colon, and rectum; Fig 1a; Supplement Table S1). All samples were processed through a unified pipeline, which involved the removal of vector sequences, low-quality bases, short reads, and host/food genomes.

After assembly, binning, quality control (with completeness ⩾ 50%, contamination ⩽ 10%, and contig length ⩾ 200 kb), and strain-level dereplication (99% ANI), we obtained a Comprehensive Ruminant gastrointestinal tract Microbiome reference genome Catalog (CRMC) consisting of 16,431 species-level MAGs and 39,696 strain-level MAGs (Fig 1b; Supplementary Fig. 1). In parallel, we also constructed host-specific microbial genome catalogs for each of the 8 ruminants (Fig 1b). The median size of the CRMC MAGs was 1.84 Mb (235.18 kb ~ 8.36 Mb; Fig 1c), with a median N50 of 16.56 kb (3.30 kb ~ 1.17 Mb; Fig 1c). The median completeness was 77.75%, and the median contamination was 1.68% (Fig 1c). The median number of protein-coding genes per MAG was 1,614 (231 ~ 8,083), of which 1,386 (186 ~ 7,324) were functionally annotated, accounting for approximately 85.87% of the protein-coding genes (Fig 1c). Approximately 93.74% of the MAGs contained multiple tRNA genes (⩾10 types), with an average of 15.37 tRNA types per MAG. According to the quality standards defined by Bowers et al. [71], approximately 46.06% of the CRMC MAGs were classified as medium-to-high quality (Fig 1b), 24.64% (9,781 MAGs) classified as medium-quality (completeness > 80%, contamination < 10%; Fig 1b), and with 21.42% (8,502 MAGs) classified as high-quality (completeness > 90%, contamination < 5%; Fig 1b).

Taxonomic annotation of the CRMC was performed using the GTDB database, revealing that the catalog consists of 981 archaea MAGs (2.47%) and 38,715 bacteria MAGs (97.53%). The

archaeal MAGs mainly belong to three phyla: Halobacteriota (predominantly family Methanocorpusculaceae and genus *Methanocorpusculum*), Methanobacteriota (predominantly family Methanobacteriaceae and genus *Methanobrevibacter*), and Thermoplasmatota (predominantly family Methanomethylophilaceae and genus *UBA71*). Among the bacterial phyla, Firmicutes_A and Bacteroidota were overwhelmingly dominant, with 15,252 MAGs (38.42%) and 14,305 MAGs (36.03%), respectively, comprising most of the ruminant GIT microbiome (Fig 1d). These two phyla were followed at much lower numbers by Firmicutes (1,826 MAGs, 4.60%), Verrucomicrobiota (1,429 MAGs, 3.60%), and Proteobacteria (1,371 MAGs, 3.45%). We found that the Firmicutes_A phylum was entirely composed of the Clostridia class, with most of its MAGs belonging to the orders Oscillospirales (7,865 MAGs, 51.57%), Lachnospirales (3,461 MAGs, 22.69%), and Christensenellales (2,427 MAGs, 15.91%), collectively accounting for 90.17% of the Firmicutes_A phylum. Similarly, the Bacteroidota phylum was entirely composed of the Bacteroidia class, with the order Bacteroidales (14,258 MAGs, 99.67%) forming the core of the phylum (Supplement Table S2). These findings further underscore the central role of the Oscillospirales, Lachnospirales, Christensenellales, and Bacteroidales orders in the ruminant GIT microbiome. Interestingly, although Bacteroidota and Firmicutes_A were the dominant phyla in all 8 ruminant hosts GIT microbiomes, their diversity varied across species (Supplementary Fig. 2, 3; Supplement Table S3-S10). Notably, the dominant phyla Bacteroidota and Firmicutes_A exhibited relatively stable proportions in confinement-fed ruminants but showed pronounced fluctuations in free-ranging species. The ratio of Firmicutes_A to Bacteroidota MAGs remained close to 1.1 in confinement-fed ruminants (buffalo, 0.93; cattle, 0.98; sheep, 1.23; goat, 1.28; Supplement Table S11). In contrast, this ratio varied markedly among free-ranging ruminants: large-bodied species

such as yak (0.36) and moose (0.32) displayed higher proportions of Bacteroidota, whereas small-bodied species such as roe deer (2.18) and water deer (2.08) exhibited greater abundances of Firmicutes_A (Fig 1d; Supplement Table S11). These results suggest that confinement-fed ruminants maintain a more balanced ratio of dominant phyla due to the stability of their feed composition, while in free-ranging ruminants, body size–associated differences in intestinal surface area may contribute to the observed divergence.

The CRMC significantly expanded the ruminant MAGs by demonstrating high coverage over existing MAG catalogs and containing additional novel ones. Specifically, at the species level (ANI 95%) the CRMC nearly encompasses the entire RGMGC catalog (8,881 MAGs constructed from 370 ruminant GIT microbiome samples [4], 85.62%; Fig 1f). Additionally, CRMC contains 12,756 (32.13%) novel ones that shared <= 95% ANIs with the microbial genomes in major public database (GTDB and RGMGC) and of model species (human, mouse, pig) (Figure 1f). Consistently, the novelty of the CRMC was further supported by the presence of 15,059 unannotated MAGs (37.94%) according to the GTDB database, which showed low similarity to existing reference genomes (ANI ≤ 95%; Fig. 1e).

As expected, the CRMC catalog showed the highest representation of the 2,325 metagenomic samples by recruiting ~83.45% of clean sequencing reads, significantly higher than any other datasets such as GTDB (74.75%), RGMGC (66.99%) and others (Fig. 1g; Supplement Table S12).

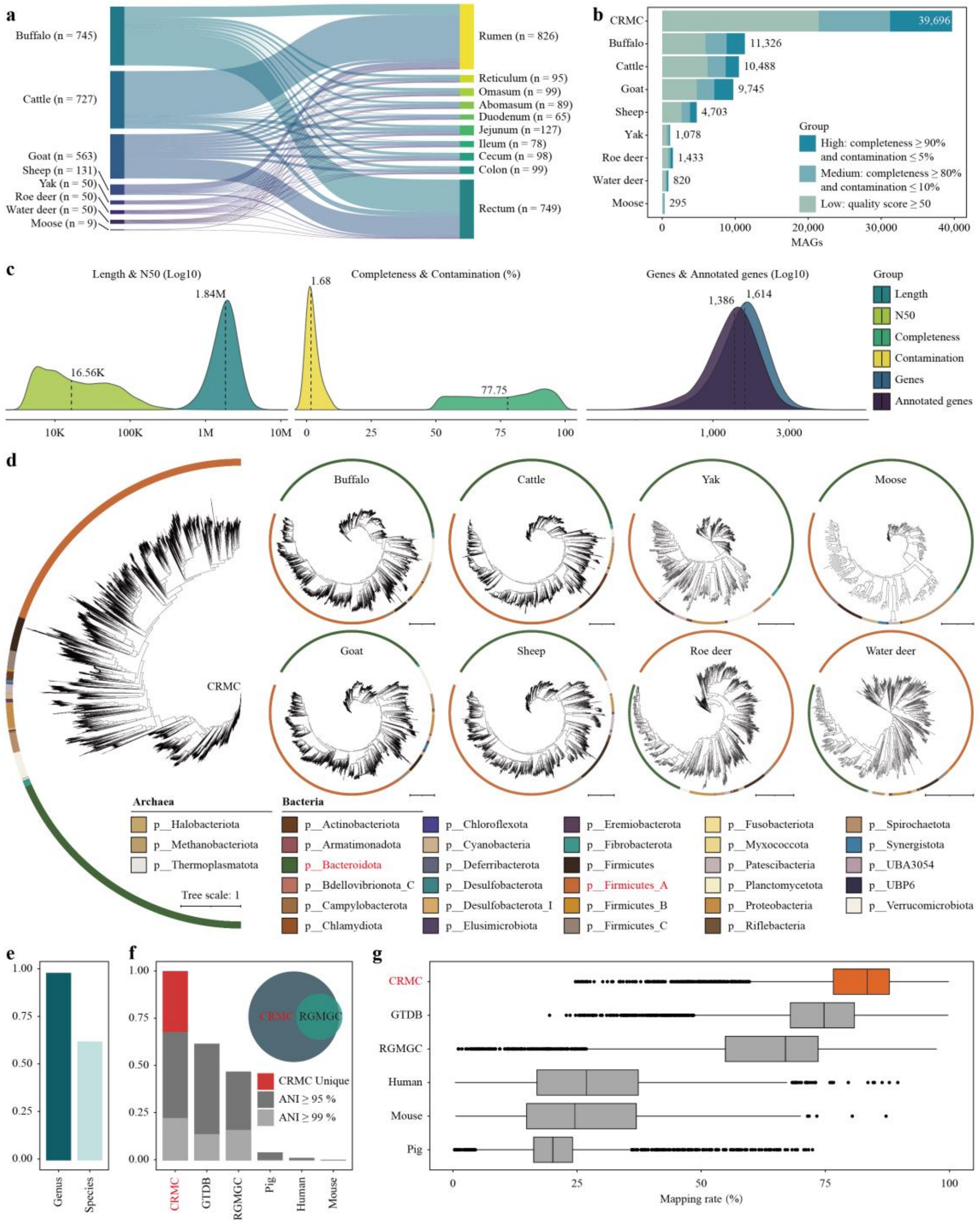

Fig. 1 Construction and evaluation of the ruminant GIT **microbiome reference genome catalog (CRMC). a** Distribution of 2,325 metagenomic samples from 8 ruminant hosts across 10 GIT regions. Connections indicate host–GIT site relationships, with numbers in parentheses representing sample counts. The GIT sites were divided into three sections: stomach (rumen, reticulum, omasum, and abomasum), small intestine (duodenum, jejunum, and ileum), and large intestine (cecum, colon,

and rectum). **b** Quality distribution of the CRMC and host-specific reference genome catalogs reconstructed in parallel. Numbers indicate the total MAGs in each catalog. Quality thresholds were defined following Bowers et al [71]. Different colors represent MAG quality: high-quality ($\geqslant$90% completeness and $\leqslant$5% contamination), medium-quality ($\geqslant$80% completeness and $\leqslant$10% contamination), and low-quality ($\geqslant$50% completeness). **c** Distribution of CRMC MAGs length, N50, completeness, contamination, number of protein-coding genes, and number of annotated genes. Different colors represent distinct categories, and dashed lines indicate medians. **d** Taxonomic classification of the CRMC and host-specific catalogs based on GTDB-Tk [60]. Different colors denote phyla. **e** Taxonomic classification rates of the CRMC MAGs at genus and species levels according to GTDB-Tk [60]. **f** Pairwise ANI [69] comparisons (95% and 99%) between the CRMC and selected public datasets (GTDB-db [60], RGMGC [4], pig [66], human [67], and mouse [68]). Red indicates the proportion of MAGs unique to the CRMC at the strain level (ANI 99%). The Venn diagram shows the coverage of the RGMGC (based on 370 samples) [4] by the CRMC at the species level (ANI 95%). **g** Reads mapping rates of 2,325 ruminant metagenomic clean data against the CRMC and selected public datasets (GTDB-db [60], RGMGC [4], pig [66], human [67], and mouse [68]).

Shared and distinctive microbial taxonomical and functional capacities across ruminant hosts

To further explore the compositional and functional similarities and differences among ruminant GIT microbiomes, we performed a cross-species comparison based on the independently reconstructed reference genome catalogs of 8 ruminant hosts. Using 95% ANI as the species-level

threshold, each host-specific catalog was compared with those of the other seven hosts (Supplementary Fig. 4; Supplement Table S13). The results showed that cattle had the highest proportion of shared species with other ruminants (6,850 MAGs, 65.31%; Fig 2a), followed by sheep (2,711 MAGs, 57.64%; Fig 2a) and goat (4,691 MAGs, 48.14%; Fig 2a), both of which are typically fed under more confinement feeding. In contrast, buffalo, which are more commonly raised under dispersed or semi-free-range conditions, exhibited the lowest proportion of shared species (2,822 MAGs, 24.92%; Fig 2a). These findings suggest that indoor feeding may drive a certain degree of convergence in the GIT microbiomes of ruminants, whereas grazing or free-range management tends to preserve host-specific microbial lineages.

We further examined the distributional features of microbial taxa shared between each ruminant GIT microbiome and those of the other hosts. Among the 8 ruminant hosts, cattle exhibited the highest proportion of shared MAGs, with the majority overlapping with other ruminants raised under indoor feeding, including sheep (45.05%; Fig 2b), goat (32.62%; Fig 2b), and buffalo (36.08%; Fig 2b). In contrast, similarity between cattle and the other hosts was much lower, including roe deer (7.03%; Fig 2b), water deer (6.11%; Fig 2b), yak (2.73%; Fig 2b), and moose (1.22%; Fig 2b). Smaller-bodied ruminants (goat, sheep, roe deer, and water deer) also shared a moderate proportion of microbial taxa, with goat and sheep exhibiting nearly 50% overlap (Fig 2b). Roe deer and water deer further shared 23.03% and 38.05% of taxa with goat (Fig 2b), respectively. Consistent with the slightly higher representation of Firmicutes_A in smaller-bodied ruminants, these results suggest that host body size may partially shape the similarity of GIT microbiomes. By contrast, free-ranging or wild ruminants, such as buffalo, yak, and moose, shared only a limited set of core symbionts with the other hosts (<25%; Fig 2b), highlighting the role of feeding practices in maintaining host-

specific microbial lineages.

Based on the shared and host-specific microbial taxa among the 8 ruminant hosts, we further investigated the distribution of bacterial and archaeal phyla as well as functional pathways associated with substrate utilization (Supplementary Fig. 5). Notably, the dominant phylum Bacteroidota exhibited a significant trend of being shared across all hosts (Fig 2c), likely reflecting its critical role in the degradation of fibrous plant materials in the ruminant foregut. In addition, most hosts showed significant sharing of phyla including Firmicutes_C, Firmicutes_B, Fibrobacterota, Spirochaetota, Actinobacteriota, Proteobacteria, and the archaeal groups Thermoplasmatota and Halobacteriota (Fig 2c), which collectively encompass the core taxa responsible for carbohydrate digestion, nutrient absorption, and methane production. By contrast, the dominant phylum Firmicutes_A was significantly shared only among cattle, sheep, and water deer (Fig 2c), a pattern that may be associated with its variable representation between large- and small-bodied ruminants. Furthermore, Firmicutes and the methanogenic archaeal phylum Methanobacteriota were significantly shared only between cattle and sheep (Fig 2c), suggesting that, beyond the core phyla, the GIT microbiomes of these indoor feeding ruminants have developed additional convergence in functional groups linked to digestion and methanogenesis.

Further analysis of protein-coding genes from shared and host-specific MAGs elucidated clear substrate utilization patterns across the 8 ruminant hosts. Substrates that exhibited a clear trend of being commonly utilized across most ruminant GIT microbiomes included plant-derived polysaccharides such as alpha-mannan, fructan, arabinogalactan, galactomannan, ulvan, cellulose, and galactoxyloglucan, together with glycosidases involved in polysaccharide breakdown (Fig 2d). These findings indicate that the core functional traits of shared microbial taxa in ruminant GIT

microbiomes are primarily centered on the utilization and absorption of dietary polysaccharides. In contrast, ruminants raised under indoor feeding, particularly cattle and sheep, displayed unique patterns of convergence involving substrates enriched in plant-derived polysaccharides (arabinan, beta-mannan, xyloglucan, arabinogalactan protein, alpha-glucan, pectin, beta-galactan, and glycogen) as well as small-molecule derivatives such as alpha-rhamnoside, which in most other ruminant hosts tended to occur as host-specific features (Fig 2d). Notably, some substrates commonly present in formulated feeds[72-74], including beta-mannan, pectin, beta-galactan, alpha-glucan, and glycogen, were exclusively shared between cattle and sheep (Fig 2d). These results suggest that, in addition to convergent features in digestion and methanogenesis, indoor feeding cattle and sheep have also developed distinctive convergence in the utilization of plant-derived polysaccharides, differentiating them from other ruminant hosts.

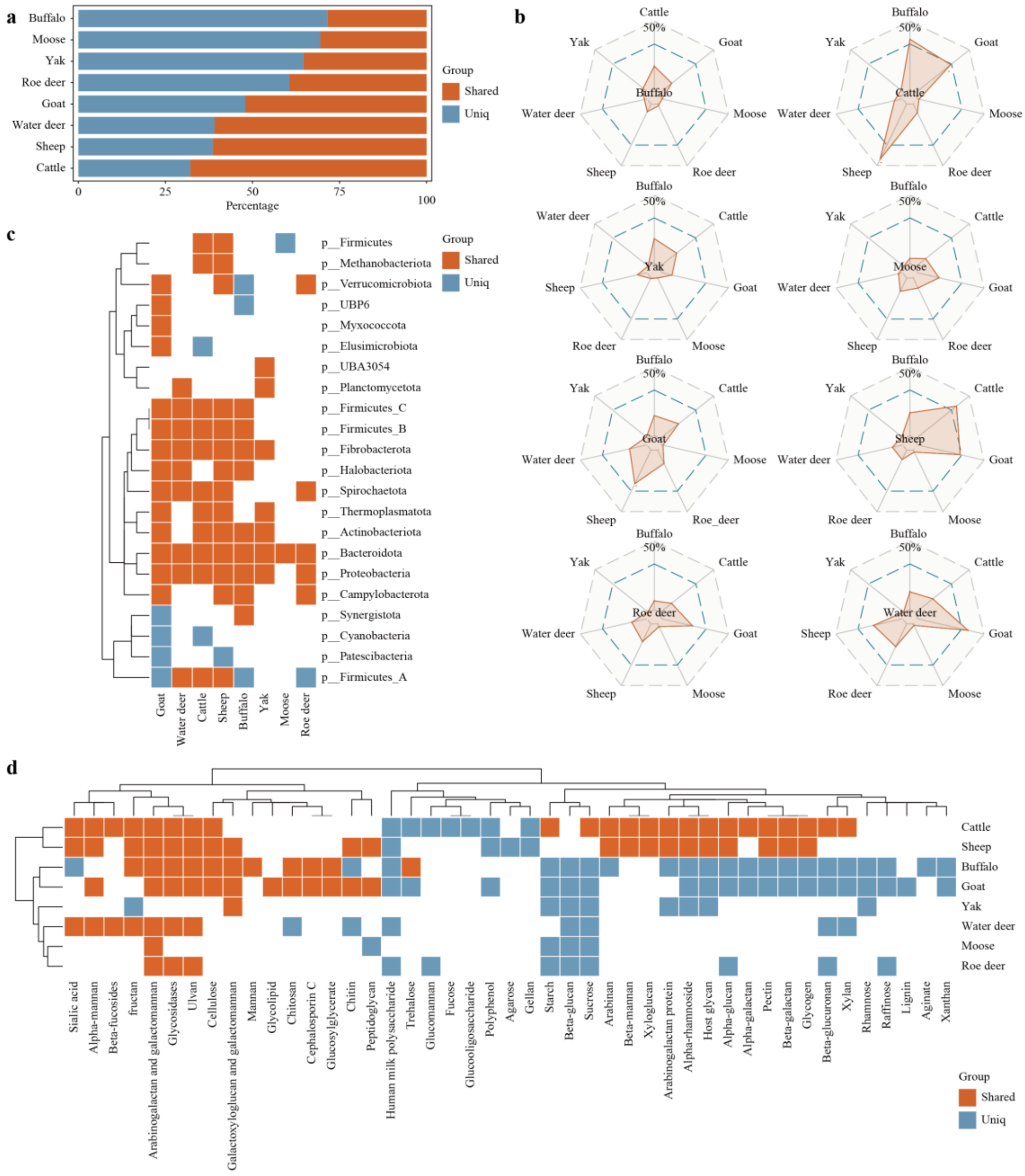

Fig. 2 Cross-host comparisons of the ruminant GIT **microbiome. a** Proportions of shared and host-specific MAGs at the species level across different ruminants. Species-level comparisons were defined using an average nucleotide identity (ANI) threshold of 95%. MAGs with ANI ⩾95% to at least one other ruminant were considered shared (orange), whereas MAGs below this threshold were classified as unique (blue). **b** Radar plot showing the proportion of shared taxa within each ruminant host relative to its own MAG collection. Shared MAGs were defined same as a. Shaded areas indicate the proportion of shared MAGs. The blue dashed line represents 25%, and the gray

dashed line represents 50%. **c** Distribution of significantly shared and host-specific phyla among ruminants. Statistical criteria for defining significantly shared or specific phyla are described in the Methods. Orange indicates phyla significantly shared across ruminants, while blue indicates phyla significantly specific to individual hosts. **d** Distribution of significantly shared and host-specific protein-coding genes related to substrate utilization among ruminants. Statistical criteria for defining significantly shared or specific substrate-utilizing genes are described in the Methods. Orange indicates shared protein-coding genes, and blue indicates host-specific protein-coding genes.

Consistent strategies for vitamin biosynthesis among ruminant GIT microbes

To characterize vitamin-synthesizing microbes in the ruminant GIT microbiome, we identified taxa carrying at least one complete biosynthetic pathway based on KEGG vitamin-synthesizing modules (thiamine, riboflavin, niacin, pantothenate, pyridoxine, biotin, folate, cobalamin, and menaquinone; Supplement Table S14). The vitamin-synthesizing microbes in the ruminant GIT were mainly derived from the dominant phyla Bacteroidota and Firmicutes_A, with additional contributions from Verrucomicrobiota, indicating that these major lineages not only function in digestion and nutrient absorption but also play important roles in host vitamin provisioning (Fig 3a; Supplement Table S15).

Across the 8 ruminants, we observed a consistent selection of the same major biosynthetic pathways by vitamin-synthesizing microbes. For thiamine, riboflavin, niacin, pantothenate, pyridoxine, and folate, nearly all vitamin-synthesizing microbes relied exclusively on a single biosynthetic pathway (M00127, M00125, M00115, M00119, M00916, and M00126, respectively;

Fig 3a; Supplement Table S15). Similarly, although multiple KEGG modules exist for biotin, cobalamin, and menaquinone biosynthesis, the microbes consistently converged on one major pathway (M00123, M00122, and M00930, respectively; Fig 3a; Supplement Table S15). Similarly, human gut vitamin-synthesizing microbes also exhibited a unified pathway preference for each vitamin, suggesting selection on convergent vitamin biosynthesis functions across ruminants and human.

Despite this functional convergence, the taxonomic composition of vitamin-synthesizing microbes varied among hosts. In line with overall differences in microbial community structure, smaller-bodied ruminants harbored a relatively higher proportion of Firmicutes_A vitamin-synthesizers compared to larger-bodied hosts, whereas Bacteroidota were more dominant in larger-bodied hosts (Fig 3a). Notably, the human gut microbiome also exhibited a higher representation of Firmicutes_A vitamin-synthesizers (Fig 3a), paralleling patterns observed in smaller-bodied ruminants.

To further investigate the pathway selection preferences of different vitamin biosynthetic routes across the 8 ruminant hosts, we aggregated the relative abundances of these microbes for each pathway across the different ruminant hosts. The results elucidated that, at the relative abundance level, all ruminant hosts exhibited consistent core vitamin biosynthesis pathway preferences (Fig 3a, b). Microbial taxa responsible for the synthesis of niacin, pyridoxine, and folate, which are essential for host nutrition, showed relatively high abundances (Fig 3b). Notably, niacin and pyridoxine are vitamins that the host cannot synthesize on its own and rely on GIT microbes for supply. Although the number of vitamin-synthesizing MAGs varied across hosts, the overall abundance of microbes corresponding to each biosynthetic pathway remained similar among the

ruminants (Fig 3b; Supplementary Fig. 10). This indicates an interaction potential between the ruminant host and its GIT vitamin-synthesizing microbiota, maintaining a stable level of microbial abundance to meet the host's nutritional needs.

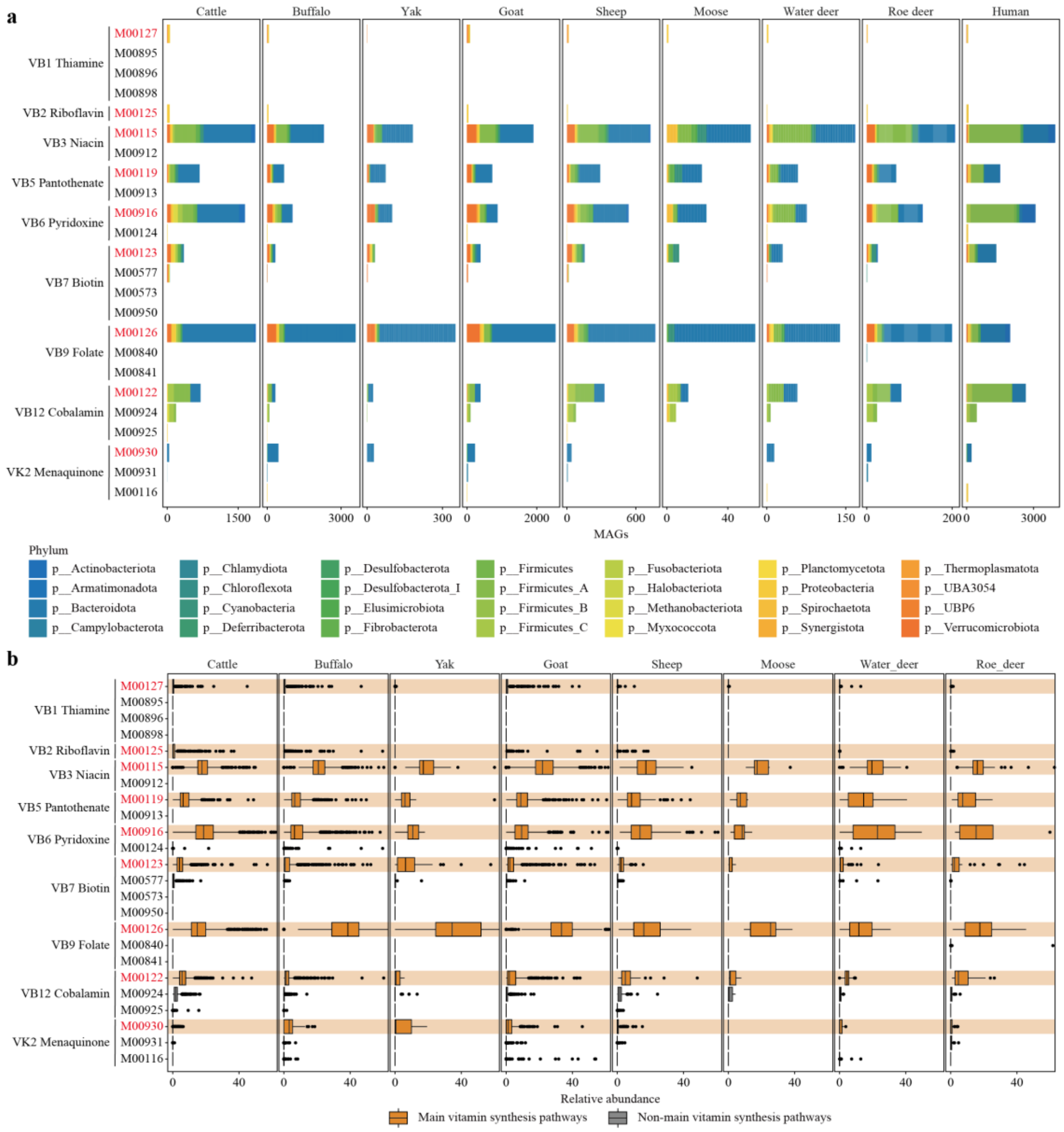

Fig. 3 Identification and functional pathway assessment of vitamin-synthesizing microbes in the ruminant GIT **microbiome. a** Distribution of vitamin-synthesizing MAGs across the 8 ruminant hosts. Pathway information was obtained from the KEGG database (Table S2). Vitamin-synthesizing MAGs were defined as those containing the complete set of pathway nodes, with multi-

gene nodes required to include at least one functional enzyme. Each color represents a different phylum, and bar length indicates the number of MAGs. The color coding for phyla is consistent between the ruminant and human datasets. Pathways highlighted in red denote the biosynthetic routes consistently selected across ruminants. **b** Relative abundance of vitamin-synthesizing MAGs assigned to each pathway across different ruminant hosts. Relative abundances were calculated by aggregating the total abundance of MAGs assigned to the corresponding pathway shown in panel a. Each point represents clean data from an individual ruminant GIT sample. Pathways highlighted in red and boxplots shaded in orange represent the biosynthetic routes consistently selected across ruminants.

Abundance of vitamin-synthesizing microbes varies across intestinal compartments and ruminant hosts

To further investigate the distribution patterns of vitamin-synthesizing microbes across different ruminant hosts and GIT regions, we analyzed the relative abundance distribution of these microbes in 8 ruminant hosts across various GIT locations. The results showed that vitamin-synthesizing microbes with relatively low overall abundance, such as those for thiamine, riboflavin, and menaquinone, exhibited low relative abundance across all three GIT regions (stomach, small intestine, and large intestine; Supplementary Fig. 6). In contrast, vitamin-synthesizing microbes with higher overall abundance, such as those for niacin, pyridoxine, and folate, were more significantly differentiated across the GIT regions (Fig 4a). Therefore, both the stomach and large intestine are key sites for vitamin-synthesizing microbe distribution in ruminants (Supplementary Fig. 7, 8, 9). Notably, folate-synthesizing microbes, which had the highest relative abundance, were

more abundant in the large intestine than the stomach in most ruminant hosts (Fig 4a; Supplementary Fig. 7, 8, 9). While the relative abundance of vitamin-synthesizing microbes across ruminant hosts was largely consistent, folate-synthesizing microbes showed a significantly higher abundance in the large intestine of larger-bodied ruminants, such as cattle, buffalo, and yak, compared to smaller-bodied species (Fig 4a; Fig 4a; Supplementary Fig. 7, 8, 9). This suggests that the larger GIT volume in these larger ruminants may provide more ecological space for these microbes to thrive.

Further analysis of the 10 GIT locations in ruminants elucidated that vitamin-synthesizing microbes with low relative abundance, such as those for thiamine and riboflavin, were consistently distributed in the duodenum and jejunum of the small intestine across different ruminant hosts (Fig 4b). Menaquinone-synthesizing microbes were predominantly found in the rectum of the large intestine, demonstrating their location-specific distribution (Fig 4b). Vitamin-synthesizing microbes for niacin, pantothenate, pyridoxine, biotin, folate, and cobalamin, which were distributed across all 10 GIT locations, showed relatively stable abundance in the rumen, reticulum, omasum, and abomasum of the stomach (Fig 4b). Biotin-synthesizing microbes were predominantly located in the small intestine (Fig 4b). While folate-synthesizing microbes were more abundant in the large intestine than in the stomach and small intestine, cobalamin-synthesizing microbes were found at significantly lower levels in the large intestine compared to both the stomach and small intestine (Fig 4b).

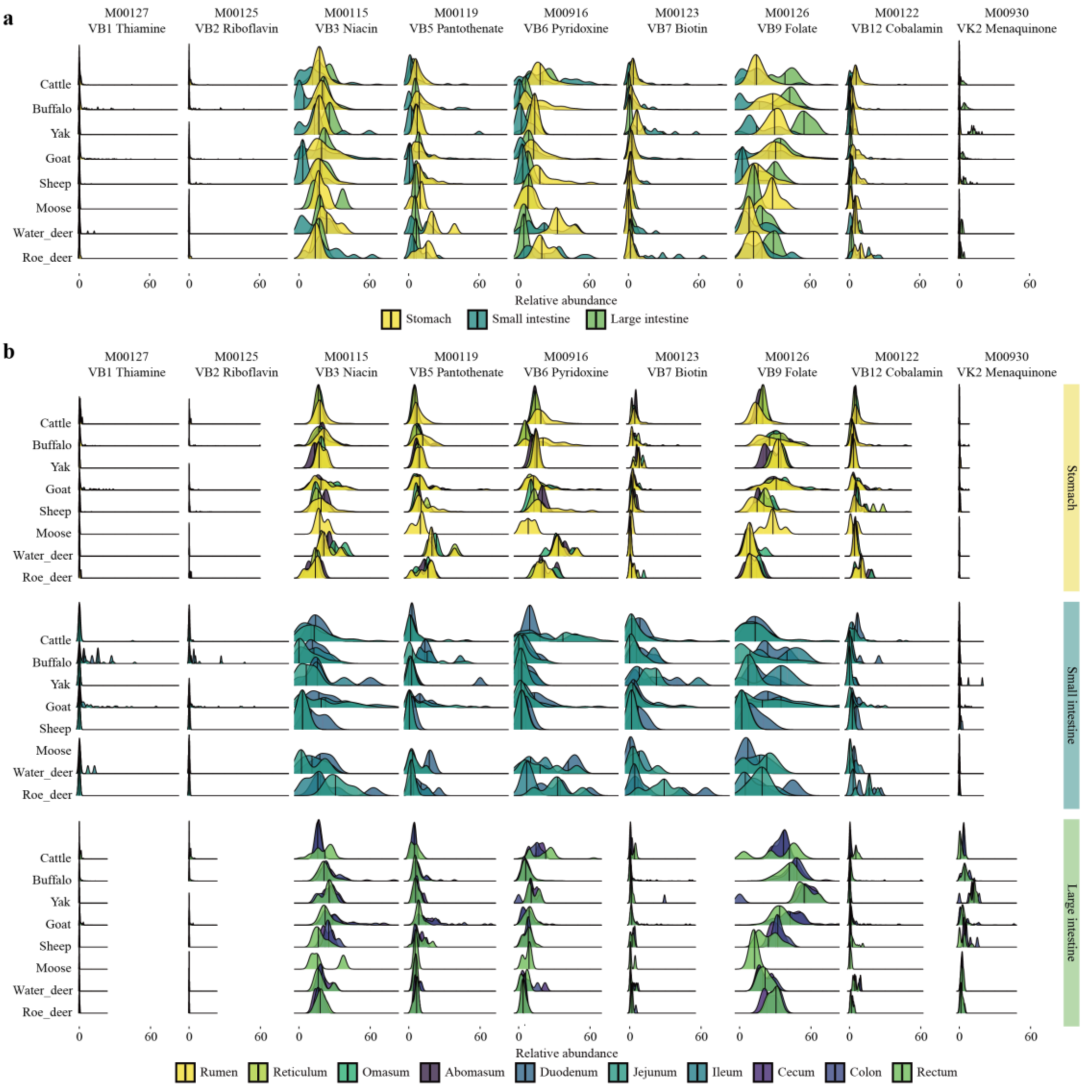

Fig. 4 Distribution of vitamin-synthesizing microbes across ruminant GIT **regions. a** Distribution of vitamin-synthesizing microbes in the stomach, small intestine, and large intestine across different ruminant hosts. Samples were grouped into three GIT sections: stomach (rumen, reticulum, omasum, and abomasum), small intestine (duodenum, jejunum, and ileum), and large intestine (cecum, colon, rectum). Different colors represent the major GIT sections. Vertical lines indicate median values. **b** Distribution of vitamin-synthesizing microbes across 10 GIT locations in ruminants. Different colors represent individual GIT sites. Vertical lines indicate median

Consistent node utilization within core vitamin biosynthetic pathways across ruminant hosts

To investigate the characteristics of gene usage within vitamin biosynthesis pathways, we conducted a comprehensive analysis of protein-coding genes in vitamin-synthesizing microbes across the 8 ruminant hosts (Supplement Table S15-S24). The results elucidated that, at multi-gene nodes where alternative enzymatic options exist, different ruminants exhibited distinct preferences. In the thiamine biosynthesis pathway, buffalo and cattle showed lower usage frequencies of K0218, K00941, and K00788 compared with goat and sheep, but a higher frequency of K12153 (Fig 5a). Since K00941, K00788, and K12153 belong to the same multi-gene node, these vitamin-synthesizing microbes maintained functional capacity through compensatory preferences, which differed from the human gut microbiome, where K12153 was not utilized but K00941 and K00788 were frequently used (Fig 5a). In the riboflavin biosynthesis pathway, buffalo, cattle, goat, and sheep, like humans, simultaneously utilized both K14652 and K01497 to enhance the efficiency of the multi-gene node, whereas the other ruminants relied exclusively on K01497 (Fig 5a). In contrast, for the niacin, pantothenate, pyridoxine, biotin, folate, cobalamin, and menaquinone pathways, both ruminant and human vitamin-synthesizing microbes displayed consistent usage frequencies, showing common preferences for specific genes. In the biotin biosynthesis pathway, microbes consistently tended to utilize K00833 and K01935 while avoiding K19563 and K19562 (Fig 5a).

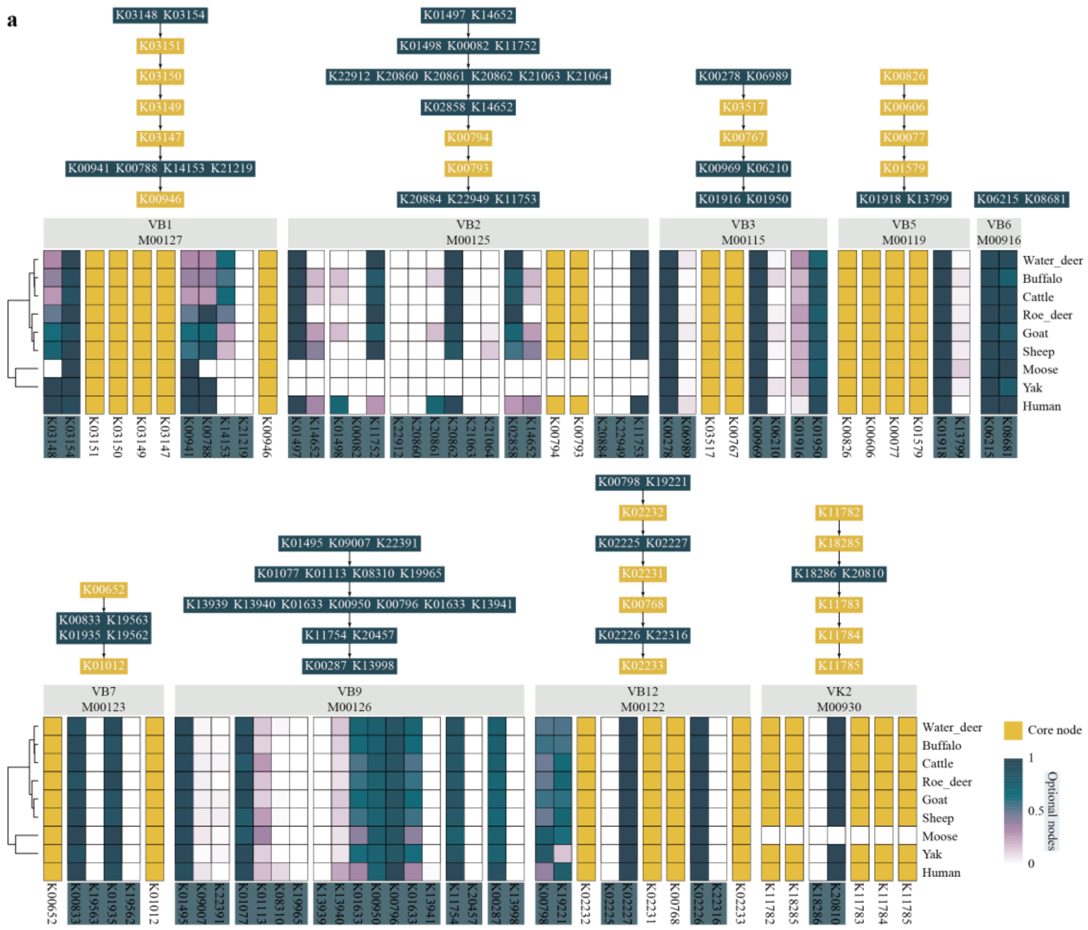

Fig. 5 Usage frequency of protein-coding genes at pathway nodes in vitamin-synthesizing microbes across ruminant GIT **microbiomes.** Pathway information was obtained from the KEGG database (Table S2). The structures of the vitamin-synthesizing pathways follow the KEGG database. Blue nodes represent multi-gene nodes, while yellow nodes represent single-gene nodes. The heatmap color scale indicates the percentage of vitamin-synthesizing MAGs in the corresponding pathway that contained the respective node genes.

Convergent selection of key steps in microbial vitamin biosynthesis pathways in the ruminant GIT

To further confirm the characteristics of multi-gene node co-selection within vitamin-synthesizing

MAGs of the ruminant GIT microbiome, we categorized all vitamin biosynthesis pathways across the 8 ruminant hosts and aggregated the relative abundances of microbes that consistently selected the same multi-gene nodes. The results showed that nearly all vitamin-synthesizing microbes exhibited consistent co-selection of multi-gene nodes across ruminants (Fig 6a; Supplementary Fig. 11). However, these co-selections did not necessarily involve choosing the maximum number of genes to ensure pathway efficiency. In the thiamine pathway, although genes such as K03148, K00941, and K00788 were additionally selected in vitamin-synthesizing MAGs, a higher relative abundance of microbes relied on K03154 and K14153 (Fig 6a). Similarly, in the riboflavin pathway, while some microbes employed K14652, the majority primarily used K01497 (Fig 6a). Comparable patterns were also observed in other pathways, including niacin (higher relative abundance of K01950 compared to K01916; Fig 6a), pantothenate (K01918 compared to K13799; Fig 6a), and folate (K01495, K01077, K01633, K00950, and K00796 compared to K09007, K22391, K01113, and K13940; Fig 6a). In contrast, the pyridoxine and cobalamin pathways consistently exhibited multi-gene co-selection across ruminants, with pyridoxine-synthesizing microbes simultaneously selecting K06215 and K08681 (Fig 6a), and cobalamin-synthesizing microbes selecting K00798 and K19221 (Fig 6a).

Overall, we identified two major patterns: (i) thiamine, riboflavin, niacin, pantothenate, and folate pathways were characterized by the preferential selection of core multi-gene nodes without further expansion to additional redundant genes, and (ii) pyridoxine and cobalamin pathways involved multi-gene co-selection to enhance node functionality. Importantly, we observed that these patterns were consistent across all 8 ruminant hosts, and the relative abundances of microbes following each selection mode remained at similar levels, which highlights the functional stability

of vitamin-synthesizing microbes across ruminant hosts, despite variations in GIT microbial diversity.

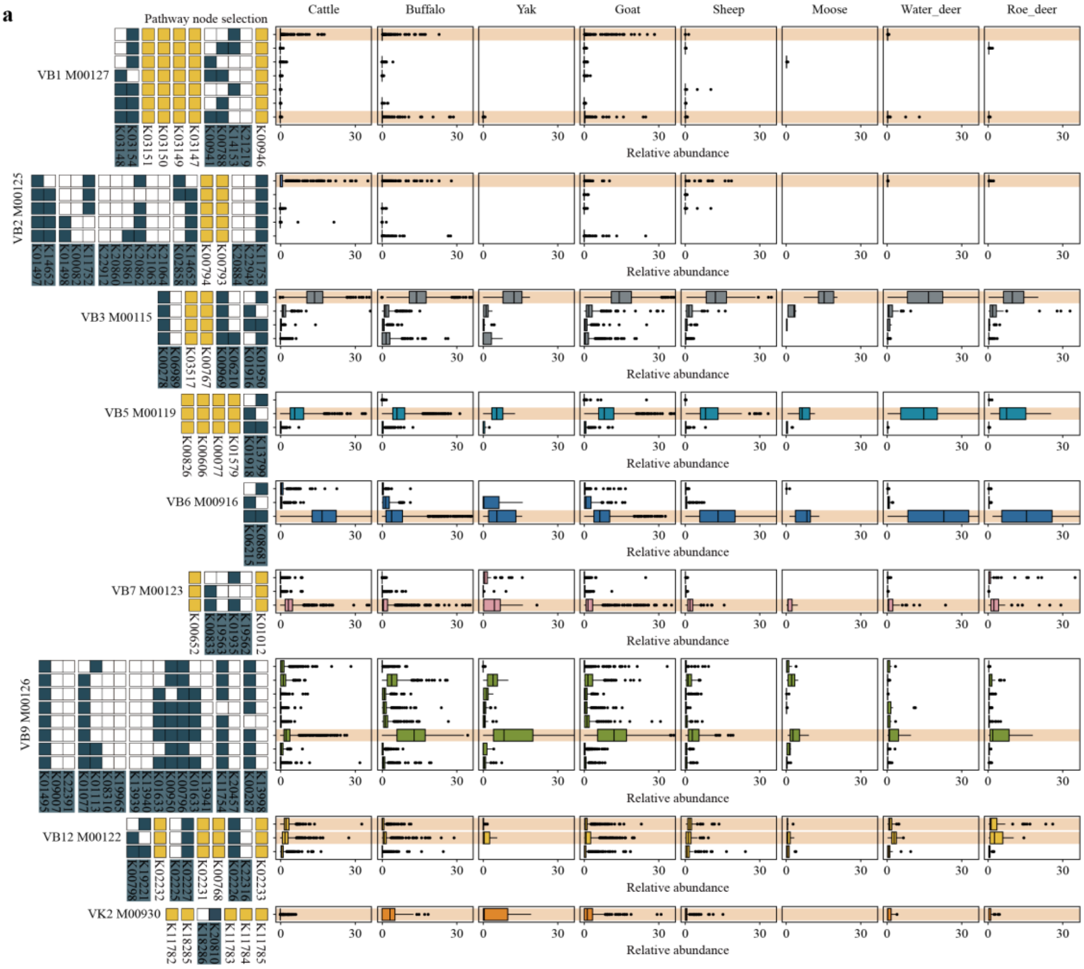

Fig. 6 Relative abundance of co-selection patterns at multi-gene nodes in vitamin-synthesizing microbes across ruminant GIT **microbiomes.** Pathway information was obtained from the KEGG database (Table S2). The structures of the vitamin-synthesizing pathways follow the KEGG database. Blue nodes represent multi-gene nodes, while yellow nodes represent single-gene nodes. Relative abundance was calculated as the sum of MAGs harboring the same co-selected multi-gene nodes. Each point represents clean data from an individual ruminant GIT sample. Orange bars denote the co-selection patterns consistently observed across ruminants, highlighting the stability of

vitamin-synthesizing function across diverse ruminant hosts.

Discussion

The ruminant-specific stomach provides a rich natural reservoir of microbial resources, which plays a critical role in digestion, nutrient absorption, and maintaining host homeostasis [1-5]. Although Fei et al. (2021) constructed a ruminant gastrointestinal tract (GIT) microbiome genome catalog (RGMGC) based on 370 samples [4], given the current scale of ruminant microbiome research, there remains a pressing need for reference collections with larger sample sizes and broader species coverage.

To fill this gap, we comprehensively reconstructed the ruminant GIT microbiome reference genome catalog (CRMC) using 2,325 metagenomic clean data (from 21 NCBI projects; Supplement Table S1), which encompassed 8 ruminant hosts (buffalo, cattle, goat, sheep, yak, roe deer, water deer and moose; Figure 1a; Supplement Table S1) and across 10 GIT regions (rumen, reticulum, omasum, abomasum, duodenum, jejunum, ileum, cecum, colon and rectum; Figure 1a; Supplement Table S1). In parallel, we applied the same standardized analytical pipeline to independently generate species-specific reference genome catalogs for each host, enabling cross-host comparative analyses. The CRMC comprises 39,696 non-redundant MAGs (Fig 1b), including 981 archaeal MAGs (2.47%) and 38,715 bacterial MAGs (97.53%; Fig 1c). Although this study did not involve third-generation sequencing data, resulting in fewer MAGs reaching complete status, the CRMC not only nearly covered the existing RGMGC (8,881 MAGs, 85.62%; Fig 1f) but also provided 15,059 MAGs (37.94%; Fig 1e) lacking species-level annotations, and contributed 12,756 novel

MAGs (32.13%; Fig 1f) beyond current datasets. Furthermore, reads mapping rates improved markedly, from 66.99% with RGMGC to 83.35% with the CRMC (Fig 1g), significantly expanding the scope of ruminant GIT microbiome reference systems and providing a direct and reliable dataset for future studies. In future studies, the incorporation of third-generation sequencing data will further enhance the CRMC, yielding more complete-level MAGs. Beyond serving as a comprehensive reference, the CRMC also provides a robust platform for functional microbiota mining, comparative host-microbiome analyses, and the rational design of synthetic microbial communities, thereby advancing both fundamental understanding of ruminant digestion and nutrient utilization and practical applications in animal husbandry.

Based on the reconstructed ruminant GIT microbiome reference genome catalog, we conducted cross-host comparative analyses among the 8 ruminant hosts to explore shared and host-specific microbial features.

First, we elucidated host body size- and diet-driven patterns of shared and host-specific microbial structures and functional features across the 8 ruminant hosts. Core phyla including Bacteroidota, Firmicutes_C, Firmicutes_B, Fibrobacterota, Spirochaetota, Actinobacteriota, and Proteobacteria were prevalently shared across ruminant GIT microbiomes (Fig 2c). These phyla collectively underpin the essential functions of digestion and methanogenesis, whereas the dominant phylum Firmicutes_A was significantly shared only between cattle and sheep under indoor feeding conditions (Fig 2c). At the functional level, the utilization of dietary polysaccharides such as alpha-mannan, fructan, arabinogalactan, galactomannan, ulvan, cellulose, and galactoxyloglucan, together with glycosidases involved in polysaccharide breakdown, constituted core features consistently shared across ruminants (Fig 2d). By contrast, the utilization of arabinan, beta-mannan, xyloglucan,

arabinogalactan protein, alpha-glucan, pectin, beta-galactan, glycogen, and small-molecule derivatives such as alpha-rhamnoside tended to show host-specific patterns (Fig 2d). Notably, some substrates commonly present in formulated feeds, including beta-mannan, pectin, beta-galactan, alpha-glucan, and glycogen, were exclusively shared between cattle and sheep under indoor feeding conditions (Fig 2d). These findings indicate that ruminants harbor a common set of core microbial taxa and functional genes. However, host body size and feeding practices can drive divergence in microbial composition and function. Under indoor feeding conditions, the frequent presence of feed-associated polysaccharides appears to have promoted functional convergence between cattle and sheep.

Second, we focused on the biosynthetic characteristics and GIT distribution of vitamin-synthesizing microbes across the 8 ruminant hosts. Vitamin-synthesizing microbes were primarily derived from Bacteroidota, Firmicutes_A, and Verrucomicrobiota, indicating that the dominant phyla Bacteroidota and Firmicutes_A not only participate in digestion and nutrient absorption but also play critical roles in microbial regulation of vitamin levels (Fig 3a). Moreover, members of Verrucomicrobiota (Fig 3a), including putative probiotics, may exert beneficial effects through their involvement in vitamin biosynthesis. Despite taxonomic differences in vitamin-synthesizing communities among ruminants (including comparisons with humans), microbes consistently selected the same pathway for each vitamin, and the total relative abundance of microbes within each biosynthetic route remained at similar levels (Fig 3b). We speculate that this functional convergence is driven by shared environmental selective pressures within the gut, such as stable temperature, anaerobic conditions, and consistent physicochemical properties. Consequently, the vitamin-synthesizing bacteria that stably colonize these gut environments hold significant potential

for identification and application as next-generation probiotics. This functional convergence highlights the stability of vitamin biosynthesis across diverse host-associated microbiomes. At the spatial level, analysis of vitamin-synthesizing microbes across different GIT locations elucidated distinct distributional patterns. Thiamine-, riboflavin-, and menaquinone-synthesizing microbes were predominantly distributed in the small intestine or large intestine (Fig 4b). Biotin-synthesizing microbes were mainly concentrated in the small intestine, whereas cobalamin-synthesizing microbes were markedly less abundant in the large intestine compared with the stomach and small intestine (Fig 4b). In contrast, vitamin-synthesizing microbes in the stomach were evenly distributed across all four compartments (Fig 4b). Together, these findings demonstrate that while ruminant GIT microbiomes maintain stable overall vitamin biosynthetic potential, the distribution of vitamin-synthesizing microbes is location-specific. This underscores the importance of conducting targeted studies that account for the spatial distribution of vitamin-synthesizing microbes within the ruminant GIT.

Last, we further uncovered the node-level characteristics of vitamin-synthesizing microbes in the ruminant GIT, focusing on their selection preferences and co-selection patterns at multi-gene nodes. The results elucidated that, at multi-gene nodes where alternative enzymatic options exist, different ruminants exhibited distinct preferences. In the thiamine biosynthesis pathway, buffalo and cattle showed lower usage frequencies of K0218, K00941, and K00788 compared with goat and sheep, but a higher frequency of K12153 (Fig 5a). Since K00941, K00788, and K12153 belong to the same multi-gene node, these vitamin-synthesizing microbes maintained functional capacity through compensatory selection preferences (Fig 5a). This pattern contrasted with the human gut microbiome, where K12153 was not utilized but K00941 and K00788 were frequently employed

(Fig 5a). These findings indicate that ruminant GIT vitamin-synthesizing microbes exhibit host-specific selection preferences in thiamine and riboflavin pathways. Nevertheless, they overall maintain consistent patterns of functional gene usage rather than indiscriminately expanding all alternative genes at multi-gene nodes. Within the multi-gene co-selection patterns of vitamin biosynthetic pathways, vitamin-synthesizing microbes among the 8 ruminant hosts showed remarkable consistency (Fig 6a). Despite differences in the frequency of individual gene usage within multi-gene nodes, the thiamine, riboflavin, niacin, pantothenate, and folate pathways were characterized by the preferential selection of core multi-gene nodes without further expansion to redundant genes (Fig 6a). By contrast, the pyridoxine and cobalamin pathways consistently involved multi-gene co-selection to enhance node functionality (Fig 6a). Importantly, the relative abundance of microbes following these core co-selection patterns remained at similar levels across ruminant hosts, which highlights the functional stability of vitamin-synthesizing microbes despite variations in GIT microbial diversity.

In summary, by comprehensively reconstructing the ruminant GIT microbiome reference genome catalog CRMC together with species-specific catalogs from 8 ruminant hosts, we provided an integrated framework that reveals both the compositional structure and functional distribution patterns of ruminant GIT microbiomes. This resource not only lays the foundation for strain-level investigations of ruminant-associated microbes but also offers critical insights for elucidating microbial contributions to digestion and vitamin-synthesizing.

Conclusions

In this study, we reconstructed the comprehensive ruminant gastrointestinal tract (GIT) microbiome reference genome catalog CRMC, together with 8 host-specific vitamin genome catalogs, thereby effectively expanding the reference range of ruminant GIT microbiomes. Furthermore, we elucidated the core microbial taxa and functional features across ruminants, as well as the compositional diversity, distribution patterns, biosynthetic pathway characteristics, and functional stability of vitamin-synthesizing microbes.

Availability of Source Code and Requirements

Project name: CRMC

Project homepage: https://github.com/fengtong-bio/CRMC

License: MIT License

Operating system: Linux

Programming language: Shell, Perl, R

Package management: Conda

Hardware requirements: High-performance computing nodes with $\geq$128 cores and $\geq$1TB RAM

Data availability

The ruminant GIT microbiome reference genome catalog CRMC used in this study are available in the Figshare database under accession code 30580403 (CRMC strain level catalog part 1)[75] , 30580667 (CRMC strain level catalog part 2, CRMC species level catalog and CRMC-sheep strain

level catalog)[76] and 30580790 (CRMC-buffalo, CRMC-cattle, CRMC-goat, CRMC-yak, CRMC-moose, CRMC-water deer and CRMC-roe deer strain level catalog)[77] .

Ethical Approval and Consent to participate

Not applicable.

Consent for publication

Not applicable.

Competing interests

The authors declare that they have no competing interests.

Funding

The present study was granted and supported by the Double First-Class Construction Funds for Provincial Departments Affiliated Universities in Hubei Province (5001170159 to WHC).

Authors' contributions

WHC designed and directed the research. Conceptualization, data curation: TF and WYJ; Formal analysis, investigation, visualization: TF; Methodology: TF, WYJ, YXX, and WHC; Writing-

original draft: TF; Writing-review and editing: all authors; Funding acquisition: WHC; Supervision: WHC. The author(s) read and approved the final manuscript.

Acknowledgments

Not applicable.

Additional file 1

Fig. 1-6. Fig. 1 Construction and evaluation of the ruminant gastrointestinal tract microbiome reference genome catalog (CRMC). Fig. 2 Cross-host comparisons of the ruminant gastrointestinal tract microbiome. Fig. 3 Identification and functional pathway assessment of vitamin-synthesizing microbes in the ruminant gastrointestinal tract microbiome. Fig. 4 Distribution of vitamin-synthesizing microbes across ruminant gastrointestinal tract regions. Fig. 5 Usage frequency of protein-coding genes at pathway nodes in vitamin-synthesizing microbes across ruminant gastrointestinal tract microbiomes. Fig. 6 Relative abundance of co-selection patterns at multi-gene nodes in vitamin-synthesizing microbes across ruminant gastrointestinal tract microbiomes.

Additional file 2

Supplementary Fig. 1-11. Supplementary Fig. 1 Methodological overview of our metagenomic analysis workflow, Supplementary Fig. 2 Proportion of MAGs phylum distribution in the reference genome of gastrointestinal microbiota in 8 ruminant hosts, Supplementary Fig. 3 Annotation ratio of MAGs at different taxonomic annotation levels for reference genomes of gastrointestinal microbiota in 8 ruminant hosts, Supplementary Fig. 4 Distribution of MAGs Shared/Uniq between 8 ruminant hosts and other ruminants in different phyla proportions, Supplementary Fig. 5 Distribution of MAGs Shared/Uniq between 8 ruminant hosts and other ruminants in different functional substrate proportions, Supplementary Fig. 6 Relative abundance distribution of core vitamin-synthesis pathway bacteria in the gastrointestinal tract of 8 ruminant hosts, Supplementary Fig. 7 Relative abundance distribution of core vitamin-synthesis pathway bacteria in the gastrointestinal tract of 8 ruminant hosts in stomach, Supplementary Fig. 8 Relative abundance

distribution of core vitamin-synthesis pathway bacteria in the gastrointestinal tract of 8 ruminant hosts in small intestine, Supplementary Fig. 9 Relative abundance distribution of core vitamin-synthesis pathway bacteria in the gastrointestinal tract of 8 ruminant hosts in large intestine, Supplementary Fig. 10 Distribution of relative abundance of MAGs synthesized from different vitamins in the gastrointestinal tract of 8 ruminant hosts, Supplementary Fig. 11 Relative abundance distribution of MAGs in core node patterns of different vitamin synthesis bacteria in the gastrointestinal tract of 8 ruminant hosts.

Additional file 3

Supplement Table S1-S24. Table S1 Sampling information for 2325 samples, Table S2 Number of MAGs at different genera in CRMC, Table S3 Number of MAGs at different genera in buffalo, Table S4 Number of MAGs at different genera in cattle, Table S5 Number of MAGs at different genera in goat, Table S6 Number of MAGs at different genera in moose, Table S7 Number of MAGs at different genera in roe deer, Table S8 Number of MAGs at different genera in sheep, Table S9 Number of MAGs at different genera in water deer, Table S10 Number of MAGs at different genera in yak, Table S11 Proportion of Firmicutes-A MAGs/Bacteroidota MAGs in different ruminant animals, Table S12 2325 clean data reads mapping rate (%), Table S13 Proportion of MAGs shared among ruminants, Table S14 KEGG vitamin biosynthesis pathways, Table S15 Number of MAGs in each vitamin-synthesis map, Table S16 Frequency of node usage in vitamin-synthesis map VB1 (M00127), Table S17 Frequency of node usage in vitamin-synthesis map VB2 (M00125), Table S18 Frequency of node usage in vitamin-synthesis map VB3 (M00115), Table S19 Frequency of node usage in vitamin-synthesis map VB5 (M00119), Table S20 Frequency of node usage in vitamin-

synthesis map VB6 (M00916), Table S21 Frequency of node usage in vitamin-synthesis map VB7 (M00123), Table S22 Frequency of node usage in vitamin-synthesis map VB9 (M00126), Table S23 Frequency of node usage in vitamin-synthesis map VB12 (M00122), Table S24 Frequency of node usage in vitamin-synthesis map VK2 (M00930).

References

1. Chen L, Qiu Q, Jiang Y, Wang K, Lin Z, Li Z, Bibi F, Yang Y, Wang J, Nie W: **Large-scale ruminant genome sequencing provides insights into their evolution and distinct traits**. *Science* 2019, **364**(6446):eaav6202.https://doi.org/10.1126/science.aav6202
2. Shabat SKB, Sasson G, Doron-Faigenboim A, Durman T, Yaacoby S, Berg Miller ME, White BA, Shterzer N, Mizrahi I: **Specific microbiome-dependent mechanisms underlie the energy harvest efficiency of ruminants**. *The ISME journal* 2016, **10**(12):2958-2972.https://doi.org/10.1038/ismej.2016.62
3. Tang Y, Liu X, Zhu S, Jia M, Liu J-X, Sun H-Z: **New insights into the enteric methane production based on the archaeal genome atlas of ruminant gastrointestinal tract**. *Journal of Advanced Research* 2024, **74**:13.https://doi.org/10.1016/j.jare.2024.09.016
4. Xie F, Jin W, Si H, Yuan Y, Tao Y, Liu J, Wang X, Yang C, Li Q, Yan X: **An integrated gene catalog and over 10,000 metagenome-assembled genomes from the gastrointestinal microbiome of ruminants**. *Microbiome* 2021, **9**(1):137.https://doi.org/10.1186/s40168-021-01078-x
5. Wu Y, Gao N, Sun C, Feng T, Liu Q, Chen W-H: **A compendium of ruminant gastrointestinal phage genomes revealed a higher proportion of lytic phages than in any other environments**. *Microbiome* 2024, **12**(1):69.https://doi.org/10.1186/s40168-024-01784-2
6. Tong F, Wang T, Gao NL, Liu Z, Cui K, Duan Y, Wu S, Luo Y, Li Z, Yang C: **The microbiome of the buffalo digestive tract**. *Nature Communications* 2022, **13**(1):823.https://doi.org/10.1038/s41467-022-28402-9
7. Cao Y, Feng T, Wu Y, Xu Y, Du L, Wang T, Luo Y, Wang Y, Li Z, Xuan Z: **The multi-kingdom microbiome of the goat gastrointestinal tract**. *Microbiome* 2023, **11**(1):219.https://doi.org/10.1186/s40168-023-01651-6
8. Liu X, Tang Y, Chen H, Liu J-X, Sun H-Z: **Rumen DNA virome and its relationship with feed efficiency in dairy cows**. *Microbiome* 2025, **13**(1):14.https://doi.org/10.1186/s40168-024-02019-0
9. Jiang Q, Lin L, Xie F, Jin W, Zhu W, Wang M, Qiu Q, Li Z, Liu J, Mao S: **Metagenomic insights into the microbe-mediated B and K2 vitamin biosynthesis in the gastrointestinal microbiome of ruminants**. *Microbiome* 2022, **10**(1):109.https://doi.org/10.1186/s40168-022-01298-9
10. Kon S, Porter J: **The intestinal synthesis of vitamins in the ruminant**. *Vitamins & Hormones* 1954, **12**:53-68.https://doi.org/10.1016/s0083-6729(08)61008-0
11. González-Montaña J-R, Escalera-Valente F, Alonso AJ, Lomillos JM, Robles R, Alonso ME: **Relationship between vitamin B12 and cobalt metabolism in domestic ruminant: an update**. *Animals* 2020, **10**(10):1855.https://doi.org/10.3390/ani10101855
12. Tang Y, Zhan P, Wu Y, Zhang T, Yin D, Gao Y, Yu Y, Qiu S, Zhao J, Zhang X: **Landscape of mobile genetic elements and their functional cargo across the gastrointestinal tract microbiomes in ruminants**. *Microbiome* 2025, **13**(1):162.https://doi.org/10.1186/s40168-025-02139-1
13. Hanna M, Jaqua E, Nguyen V, Clay J: **Vitamins: functions and uses in medicine**. *Perm J* 2022, **26**(2):89-97.https://doi.org/10.7812/TPP/21.204
14. Liu Z, Farkas P, Wang K, Kohli MO, Fitzpatrick TB: **B vitamin supply in plants and humans:**

the importance of vitamer homeostasis. *The Plant Journal* 2022, **111**(3):662-682.https://doi.org/10.1111/tpj.15859

15. Manson JE, Cook NR, Lee I-M, Christen W, Bassuk SS, Mora S, Gibson H, Gordon D, Copeland T, D'Agostino D: **Vitamin D supplements and prevention of cancer and cardiovascular disease**. *New England Journal of Medicine* 2019, **380**(1):33-44.https://doi.org/10.1056/NEJMoa1809944

16. Mumford SL, Garbose RA, Kim K, Kissell K, Kuhr DL, Omosigho UR, Perkins NJ, Galai N, Silver RM, Sjaarda LA: **Association of preconception serum 25-hydroxyvitamin D concentrations with livebirth and pregnancy loss: a prospective cohort study**. *The lancet Diabetes & endocrinology* 2018, **6**(9):725-732.https://doi.org/10.1016/S2213-8587(18)30153-0

17. Tarracchini C, Bottacini F, Mancabelli L, Lugli GA, Turroni F, van Sinderen D, Ventura M, Milani C: **Approaches to dissect the vitamin biosynthetic network of the gut microbiota**. *Microbiome Research Reports* 2025, **4**(4):N/A-N/A.https://doi.org/10.20517/mrr.2025.66

18. Hessler T, Huddy RJ, Sachdeva R, Lei S, Harrison ST, Diamond S, Banfield JF: **Vitamin interdependencies predicted by metagenomics-informed network analyses and validated in microbial community microcosms**. *Nature Communications* 2023, **14**(1):4768.https://doi.org/10.1038/s41467-023-40360-4

19. Yang Y, Ke Y, Liu X, Zhang Z, Zhang R, Tian F, Zhi L, Zhao G, Lv B, Hua S: **Navigating the B vitamins: Dietary diversity, microbial synthesis, and human health**. *Cell Host & Microbe* 2024, **32**(1):12-18.https://doi.org/10.1016/j.chom.2023.12.004

20. Molina Ortiz JP, Read MN, McClure DD, Holmes A, Dehghani F, Shanahan ER: **High throughput genome scale modeling predicts microbial vitamin requirements contribute to gut microbiome community structure**. *Gut Microbes* 2022, **14**(1):2118831.https://doi.org/10.1080/19490976.2022.2118831

21. Chen X, Gao M, Wang L, Qiang G, Wu Y, Huang H, Kang G: **A synthetic microbial consortium protects against obesity by regulating vitamin B6 metabolism**. *Gut Microbes* 2024, **16**(1):2304901.https://doi.org/10.1080/19490976.2024.2304901

22. Torres Manno MA, Gizzi FO, Martín M, Espariz M, Magni C, Blancato VS: **Metagenomic approach to infer rumen microbiome derived traits of cattle**. *World Journal of Microbiology and Biotechnology* 2023, **39**(9):250.https://doi.org/10.1007/s11274-023-03694-1

23. Svartström O, Alneberg J, Terrapon N, Lombard V, de Bruijn I, Malmsten J, Dalin A-M, El Muller E, Shah P, Wilmes P: **Ninety-nine de novo assembled genomes from the moose (Alces alces) rumen microbiome provide new insights into microbial plant biomass degradation**. *The ISME journal* 2017, **11**(11):2538-2551.https://doi.org/10.1038/ismej.2017.108

24. Tanca A, Fraumene C, Manghina V, Palomba A, Abbondio M, Deligios M, Pagnozzi D, Addis MF, Uzzau S: **Diversity and functions of the sheep faecal microbiota: a multi‐omic characterization**. *Microbial biotechnology* 2017, **10**(3):541-554.https://doi.org/10.1111/1751-7915.12462

25. Kelly WJ, Leahy SC, Kamke J, Soni P, Koike S, Mackie R, Seshadri R, Cook GM, Morales SE, Greening C: **Occurrence and expression of genes encoding methyl-compound production in rumen bacteria**. *Animal Microbiome* 2019, **1**(1):15.https://doi.org/10.1186/s42523-019-0016-0

26. Yan M, Yu Z: **Viruses contribute to microbial diversification in the rumen ecosystem and**

are associated with certain animal production traits. *Microbiome* 2024, **12**(1):82.https://doi.org/10.1186/s40168-024-01791-3
27. Stewart RD, Auffret MD, Warr A, Walker AW, Roehe R, Watson M: **Compendium of 4,941 rumen metagenome-assembled genomes for rumen microbiome biology and enzyme discovery**. *Nature biotechnology* 2019, **37**(8):953-961.https://doi.org/10.1038/s41587-019-0202-3
28. Glendinning L, Genç B, Wallace RJ, Watson M: **Metagenomic analysis of the cow, sheep, reindeer and red deer rumen**. *Scientific reports* 2021, **11**(1):1990.https://doi.org/10.1038/s41598-021-81668-9
29. Greening C, Geier R, Wang C, Woods LC, Morales SE, McDonald MJ, Rushton-Green R, Morgan XC, Koike S, Leahy SC: **Diverse hydrogen production and consumption pathways influence methane production in ruminants**. *The ISME journal* 2019, **13**(10):2617-2632.https://doi.org/10.1038/s41396-019-0464-2
30. Abdul Rahman N, Parks DH, Vanwonterghem I, Morrison M, Tyson GW, Hugenholtz P: **A phylogenomic analysis of the bacterial phylum Fibrobacteres**. *Frontiers in microbiology* 2016, **6**:1469.https://doi.org/10.3389/fmicb.2015.01469
31. Solden LM, Naas AE, Roux S, Daly RA, Collins WB, Nicora CD, Purvine SO, Hoyt DW, Schückel J, Jørgensen B: **Interspecies cross-feeding orchestrates carbon degradation in the rumen ecosystem**. *Nature microbiology* 2018, **3**(11):1274-1284.https://doi.org/10.1038/s41564-018-0225-4
32. Kirchberger PC, Ochman H: **Resurrection of a global, metagenomically defined gokushovirus**. *Elife* 2020, **9**:e51599.https://doi.org/10.7554/eLife.51599
33. Weinroth MD, Scott HM, Norby B, Loneragan GH, Noyes NR, Rovira P, Doster E, Yang X, Woerner DR, Morley PS: **Effects of ceftiofur and chlortetracycline on the resistomes of feedlot cattle**. *Applied and environmental microbiology* 2018, **84**(13):e00610-00618.https://doi.org/10.1128/AEM.00610-18
34. Li F, Hitch TC, Chen Y, Creevey CJ, Guan LL: **Comparative metagenomic and metatranscriptomic analyses reveal the breed effect on the rumen microbiome and its associations with feed efficiency in beef cattle**. *Microbiome* 2019, **7**(1):6.https://doi.org/10.1186/s40168-019-0618-5
35. Al-Masaudi S, El Kaoutari A, Drula E, Redwan EM, Lombard V, Henrissat B: **A metagenomics investigation of carbohydrate-active enzymes along the goat and camel intestinal tract**. *International Microbiology* 2019, **22**(4):429-435.https://doi.org/10.1007/s10123-019-00068-2
36. Lin L, Xie F, Sun D, Liu J, Zhu W, Mao S: **Ruminal microbiome-host crosstalk stimulates the development of the ruminal epithelium in a lamb model**. *Microbiome* 2019, **7**(1):83.https://doi.org/10.1186/s40168-019-0701-y
37. Naas A, Solden L, Norbeck A, Brewer H, Hagen L, Heggenes I, McHardy A, Mackie R, Paša-Tolić L, Arntzen M: **"Candidatus Paraporphyromonas polyenzymogenes" encodes multi-modular cellulases linked to the type IX secretion system**. *Microbiome* 2018, **6**(1):44.https://doi.org/10.1186/s40168-018-0421-8
38. Bolger AM, Lohse M, Usadel B: **Trimmomatic: a flexible trimmer for Illumina sequence data**. *Bioinformatics* 2014, **30**(15):2114-2120.https://doi.org/10.1093/bioinformatics/btu170
39. Langmead B, Salzberg SL: **Fast gapped-read alignment with Bowtie 2**. *Nature methods* 2012, **9**(4):357-359.https://doi.org/10.1038/nmeth.1923

40. Williams JL, Iamartino D, Pruitt KD, Sonstegard T, Smith TP, Low WY, Biagini T, Bomba L, Capomaccio S, Castiglioni B: **Genome assembly and transcriptome resource for river buffalo, Bubalus bubalis (2 n= 50)**. *Gigascience* 2017, **6**(10):gix088.https://doi.org/10.1093/gigascience/gix088
41. Khan A, Singh K, Jaiswal S, Raza M, Jasrotia RS, Kumar A, Gurjar AKS, Kumari J, Nayan V, Iquebal MA: **Whole-genome-based web genomic resource for water buffalo (Bubalus bubalis)**. *Frontiers in Genetics* 2022, **13**:809741.https://doi.org/10.3389/fgene.2022.809741
42. Lado S, Elbers JP, Rogers MF, Melo-Ferreira J, Yadamsuren A, Corander J, Horin P, Burger PA: **Nucleotide diversity of functionally different groups of immune response genes in Old World camels based on newly annotated and reference-guided assemblies**. *BMC genomics* 2020, **21**(1):606.https://doi.org/10.1186/s12864-020-06990-4
43. Khalkhali-Evrigh R, Hedayat N, Ming L, Jirimutu: **Identification of selection signatures in Iranian dromedary and Bactrian camels using whole genome sequencing data**. *Scientific reports* 2022, **12**(1):9653.https://doi.org/10.1038/s41598-022-14376-7
44. Ren Y, MacPhillamy C, To T-H, Smith TP, Williams JL, Low WY: **Adaptive selection signatures in river buffalo with emphasis on immune and major histocompatibility complex genes**. *Genomics* 2021, **113**(6):3599-3609.https://doi.org/10.1016/j.ygeno.2021.08.021
45. Ba H, Cai Z, Gao H, Qin T, Liu W, Xie L, Zhang Y, Jing B, Wang D, Li C: **Chromosome-level genome assembly of Tarim red deer, Cervus elaphus yarkandensis**. *Scientific data* 2020, **7**(1):187.https://doi.org/10.1038/s41597-020-0537-0
46. Wang Q, Han R, Xing H, Li H: **A consensus genome of sika deer (Cervus nippon) and transcriptome analysis provided novel insights on the regulation mechanism of transcript factor in antler development**. *BMC genomics* 2024, **25**(1):617.https://doi.org/10.1186/s12864-024-10522-9
47. Lukacs M, Nymo IH, Madslien K, Våge J, Veiberg V, Rolandsen CM, Bøe CA, Sundaram AY, Grimholt U: **Functional immune diversity in reindeer reveals a high Arctic population at risk**. *Frontiers in Ecology and Evolution* 2023, **10**:1058674.https://doi.org/10.3389/fevo.2022.1058674
48. Xie F, Zhao S, Zhan X, Zhou Y, Li Y, Zhu W, Pope PB, Attwood GT, Jin W, Mao S: **Unraveling the phylogenomic diversity of Methanomassiliicoccales and implications for mitigating ruminant methane emissions**. *Genome Biology* 2024, **25**(1):32.https://doi.org/10.1186/s13059-024-03167-0
49. Davenport KM, Bickhart DM, Worley K, Murali SC, Salavati M, Clark EL, Cockett NE, Heaton MP, Smith TP, Murdoch BM: **An improved ovine reference genome assembly to facilitate in-depth functional annotation of the sheep genome**. *Gigascience* 2022, **11**:giab096.https://doi.org/10.1093/gigascience/giab096
50. Yang Y, Li B, Luo W, Xu B, Luo P, Zhang T, You Z: **A high-quality Chromosome-level reference genome assembly of white-lipped deer (Przewalskium albirostris)**. *Scientific Data* 2025, **12**(1):727.https://doi.org/10.1038/s41597-025-04796-1
51. Li X, He S-G, Li W-R, Luo L-Y, Yan Z, Mo D-X, Wan X, Lv F-H, Yang J, Xu Y-X: **Genomic analyses of wild argali, domestic sheep, and their hybrids provide insights into chromosome evolution, phenotypic variation, and germplasm innovation**. *Genome Research* 2022, **32**(9):1669-1684.https://doi.org/10.1101/gr.276769.122

52. Chen H-X, Liu Z-D, Bai X, Wu B, Song R, Yao H-C, Chen Y, Chi W, Hua Q, Cheng L: **Accurate cross-species 5mC detection for Oxford Nanopore sequencing in plants with DeepPlant**. *Nature Communications* 2025, **16**(1):3227.https://doi.org/10.1038/s41467-025-58576-x
53. Hao L, Xu W, Qi G, Xin T, Xu Z, Lei H, Song J: **GAGE is a method for identification of plant species based on whole genome analysis and genome editing**. *Communications Biology* 2022, **5**(1):947.https://doi.org/10.1038/s42003-022-03894-9
54. He J, Tian D, Li X, Wang X, Wang T, Wang Z, Zang H, He X, Zhang T, Yun Q: **A chromosome-level genome assembly for Onobrychis viciifolia reveals gene copy number gain underlying enhanced proanthocyanidin biosynthesis**. *Communications Biology* 2024, **7**(1):19.https://doi.org/10.1038/s42003-023-05754-6
55. Li D, Luo R, Liu C-M, Leung C-M, Ting H-F, Sadakane K, Yamashita H, Lam T-W: **MEGAHIT v1. 0: a fast and scalable metagenome assembler driven by advanced methodologies and community practices**. *Methods* 2016, **102**:3-11.https://doi.org/10.1016/j.ymeth.2016.02.020
56. Danecek P, Bonfield JK, Liddle J, Marshall J, Ohan V, Pollard MO, Whitwham A, Keane T, McCarthy SA, Davies RM: **Twelve years of SAMtools and BCFtools**. *Gigascience* 2021, **10**(2):giab008.https://doi.org/10.1093/gigascience/giab008
57. Kang DD, Li F, Kirton E, Thomas A, Egan R, An H, Wang Z: **MetaBAT 2: an adaptive binning algorithm for robust and efficient genome reconstruction from metagenome assemblies**. *PeerJ* 2019, **7**:e7359.https://doi.org/10.7717/peerj.7359
58. Parks DH, Imelfort M, Skennerton CT, Hugenholtz P, Tyson GW: **CheckM: assessing the quality of microbial genomes recovered from isolates, single cells, and metagenomes**. *Genome research* 2015, **25**(7):1043-1055.https://doi.org/10.1101/gr.186072.114
59. Olm MR, Brown CT, Brooks B, Banfield JF: **dRep: a tool for fast and accurate genomic comparisons that enables improved genome recovery from metagenomes through de-replication**. *The ISME journal* 2017, **11**(12):2864-2868.https://doi.org/10.1038/ismej.2017.126
60. Chaumeil P-A, Mussig AJ, Hugenholtz P, Parks DH: **GTDB-Tk: a toolkit to classify genomes with the Genome Taxonomy Database**. *Bioinformatics* 2019, **36**(6):1925-1927.https://doi.org/10.1093/bioinformatics/btz848
61. Letunic I, Bork P: **Interactive Tree of Life (iTOL) v6: recent updates to the phylogenetic tree display and annotation tool**. *Nucleic acids research* 2024, **52**(W1):W78-W82.https://doi.org/10.1093/nar/gkae268
62. Seemann T: **Prokka: rapid prokaryotic genome annotation**. *Bioinformatics* 2014, **30**(14):2068-2069.https://doi.org/10.1093/bioinformatics/btu153
63. Huerta-Cepas J, Szklarczyk D, Heller D, Hernández-Plaza A, Forslund SK, Cook H, Mende DR, Letunic I, Rattei T, Jensen LJ: **eggNOG 5.0: a hierarchical, functionally and phylogenetically annotated orthology resource based on 5090 organisms and 2502 viruses**. *Nucleic acids research* 2019, **47**(D1):D309-D314.https://doi.org/10.1093/nar/gky1085
64. Lombard V, Golaconda Ramulu H, Drula E, Coutinho PM, Henrissat B: **The carbohydrate-active enzymes database (CAZy) in 2013**. *Nucleic acids research* 2014, **42**(D1):D490-D495.https://doi.org/10.1093/nar/gkt1178
65. Chan PP, Lin BY, Mak AJ, Lowe TM: **tRNAscan-SE 2.0: improved detection and functional classification of transfer RNA genes**. *Nucleic acids research* 2021, **49**(16):9077-9096.https://doi.org/10.1093/nar/gkab688

66. Chen C, Zhou Y, Fu H, Xiong X, Fang S, Jiang H, Wu J, Yang H, Gao J, Huang L: **Expanded catalog of microbial genes and metagenome-assembled genomes from the pig gut microbiome**. *Nature communications* 2021, **12**(1):1106.https://doi.org/10.1038/s41467-021-21295-0

67. Jin H, Quan K, He Q, Kwok L-Y, Ma T, Li Y, Zhao F, You L, Zhang H, Sun Z: **A high-quality genome compendium of the human gut microbiome of Inner Mongolians**. *Nature Microbiology* 2023, **8**(1):150-161.https://doi.org/10.1038/s41564-022-01270-1

68. Lesker TR, Durairaj AC, Gálvez EJ, Lagkouvardos I, Baines JF, Clavel T, Sczyrba A, McHardy AC, Strowig T: **An integrated metagenome catalog reveals new insights into the murine gut microbiome**. *Cell Reports* 2020, **30**(9):2909-2922. e2906.https://doi.org/10.1016/j.celrep.2020.02.036

69. Jain C, Rodriguez-R LM, Phillippy AM, Konstantinidis KT, Aluru S: **High throughput ANI analysis of 90K prokaryotic genomes reveals clear species boundaries**. *Nature communications* 2018, **9**(1):5114.https://doi.org/10.1038/s41467-018-07641-9

70. Shaw J, Yu YW: **Rapid species-level metagenome profiling and containment estimation with sylph**. *Nature Biotechnology* 2024:1-12.https://doi.org/10.1038/s41587-024-02412-y

71. Bowers RM, Kyrpides NC, Stepanauskas R, Harmon-Smith M, Doud D, Reddy T, Schulz F, Jarett J, Rivers AR, Eloe-Fadrosh EA: **Minimum information about a single amplified genome (MISAG) and a metagenome-assembled genome (MIMAG) of bacteria and archaea**. *Nature biotechnology* 2017, **35**(8):725-731.https://doi.org/10.1038/nbt.3893

72. Weiss W: **Perspective and Commentary: Use of soy-based feedstuffs in low-alfalfa, high–corn silage diets for dairy cows**. *Applied Animal Science* 2024, **40**(4):478-486.https://doi.org/10.15232/aas.2024-02563

73. Srisaikham S: **A comparison of nutritional values, bioactive compounds, amino acids, and antioxidant activities of alfalfa (Medicago sativa) plant and Pellet for use as beneficial material ruminant feed**. *Walailak Journal of Science and Technology (WJST)* 2021, **18**(5):10312 (10316 pages)-10312 (10316 pages).https://doi.org/10.48048/wjst.2021.10312

74. Panahiha P, Mirzaei-Alamouti H, Kazemi-Bonchenari M, Aschenbach JR: **Growth performance, nutrient digestibility, and ruminal fermentation of dairy calves fed starter diets with alfalfa hay versus corn silage as forage and soybean oil versus palm fatty acids as fat source**. *Journal of Dairy Science* 2022, **105**(12):9597-9609.https://doi.org/10.3168/jds.2022-22165

75. Feng T, Wu Y, Xu Y, Chen W. A comprehensive ruminant microbial catalog (CRMC) reveals convergent selection for key vitamin-synthesizing pathways and genes across ruminants and human. figshare. https://doi.org/10.6084/m9.figshare.30580403

76. Feng T, Wu Y, Xu Y, Chen W. A comprehensive ruminant microbial catalog (CRMC) reveals convergent selection for key vitamin-synthesizing pathways and genes across ruminants and human. figshare.https://doi.org/10.6084/m9.figshare.30580667

77. Feng T, Wu Y, Xu Y, Chen W. A comprehensive ruminant microbial catalog (CRMC) reveals convergent selection for key vitamin-synthesizing pathways and genes across ruminants and human. figshare. https://doi.org/10.6084/m9.figshare.30580790

Figure 1

Click here to access/download;Figure;FIG1_V1.1.pdf

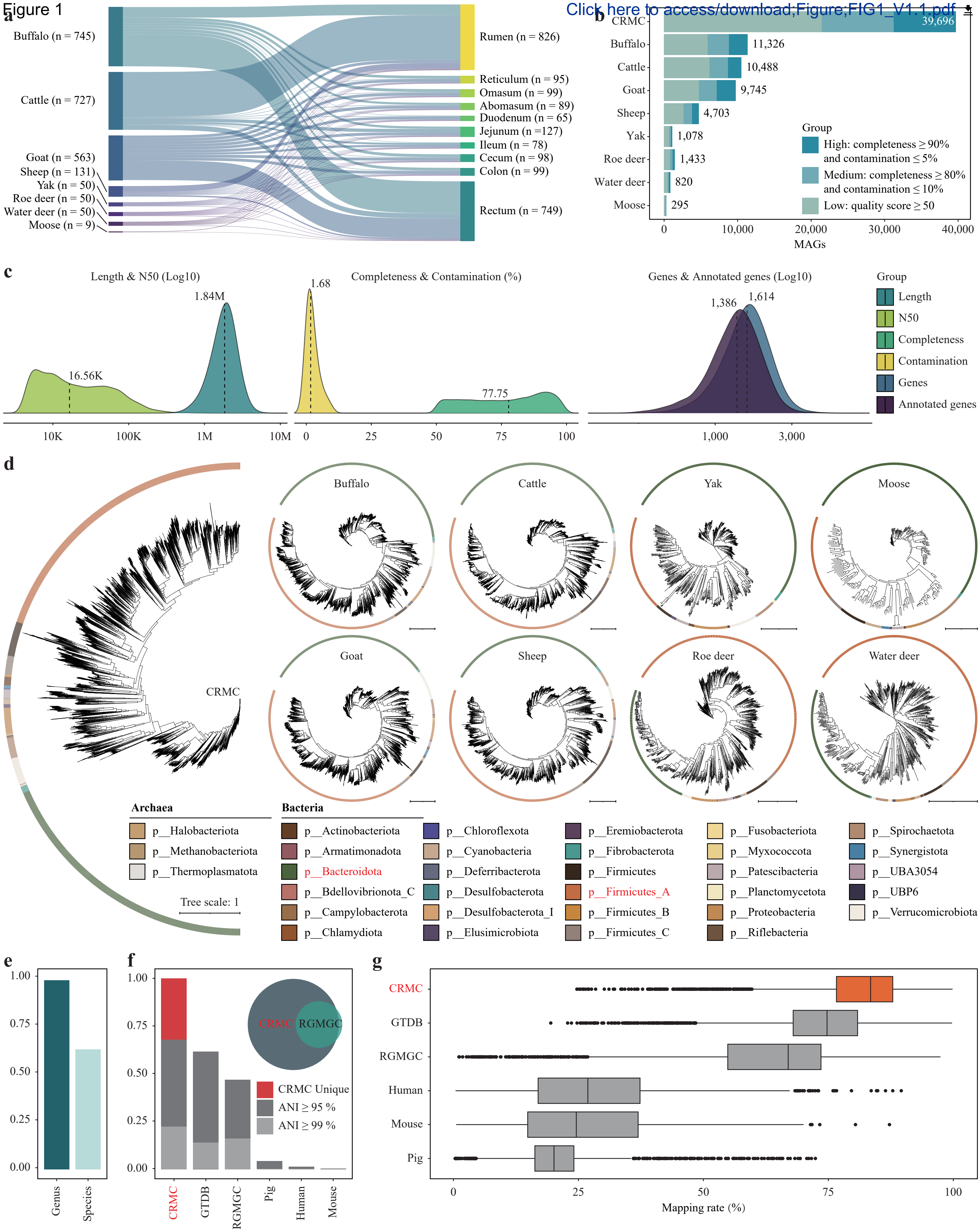

Figure 2

Click here to access/download;Figure;FIG2_V1.pdf

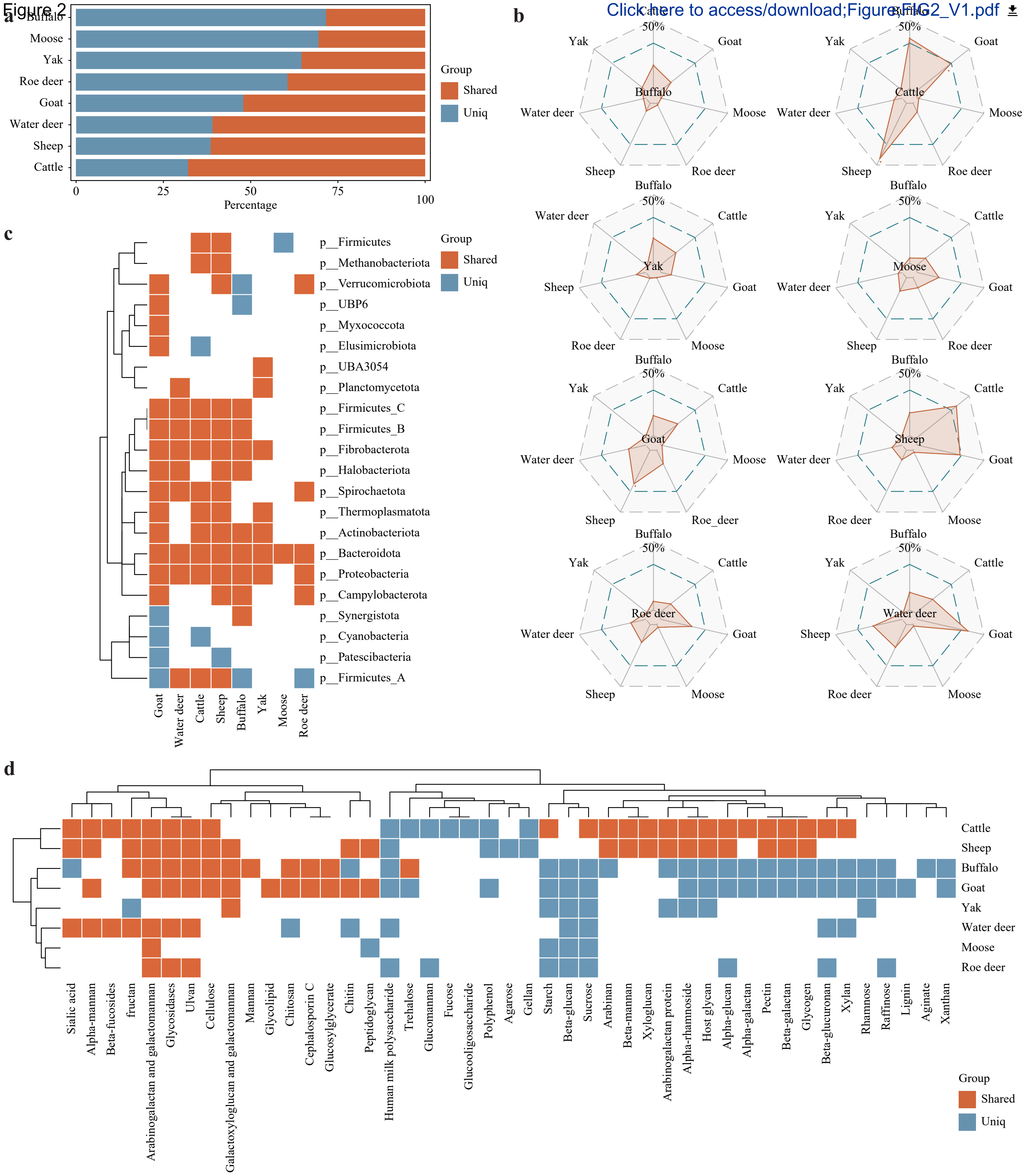

Figure 3

Click here to access/download;Figure;FIG3_V11.pdf

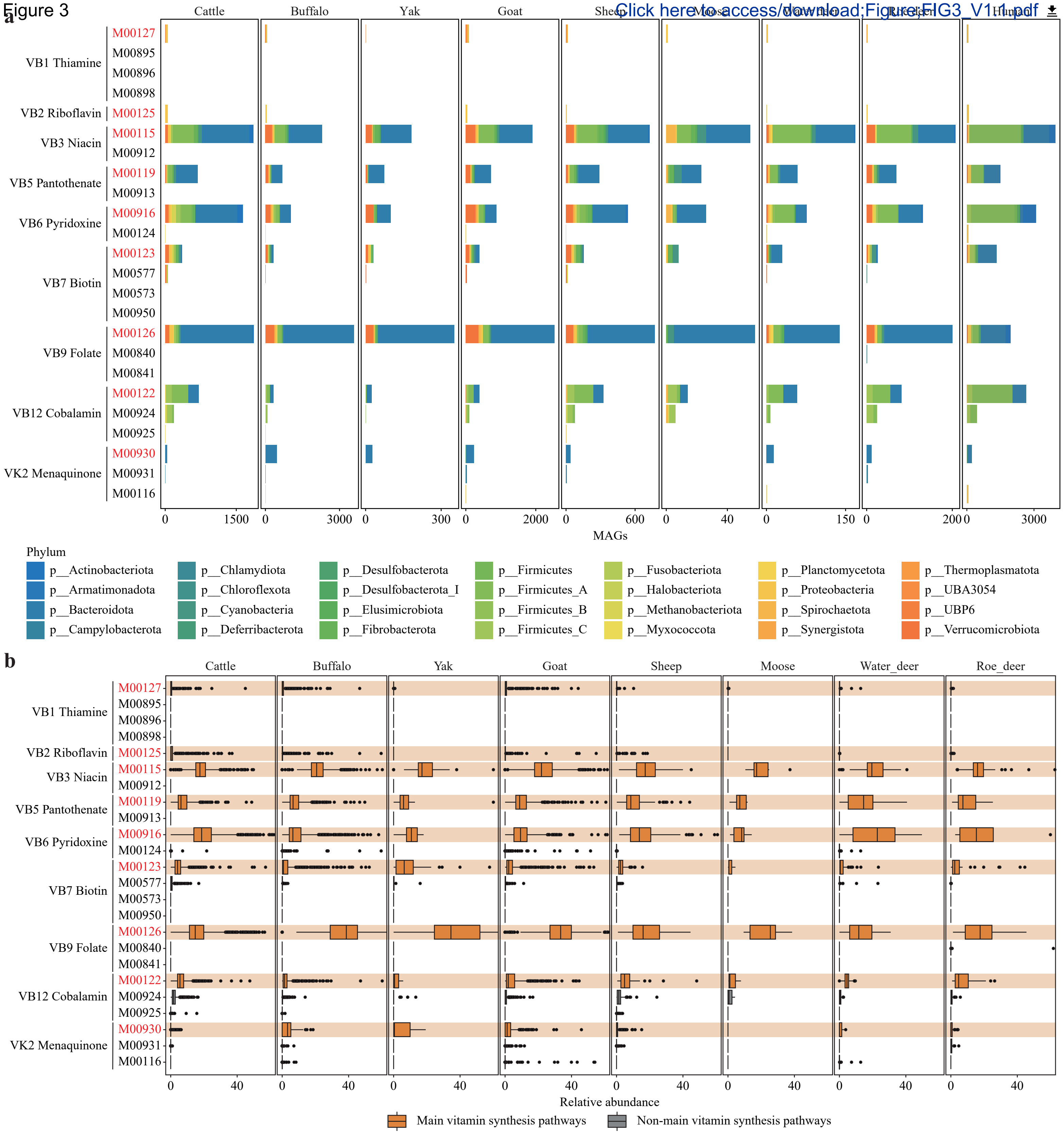

Figure 4

Click here to access/download;Figure;FIG4_V1.pdf

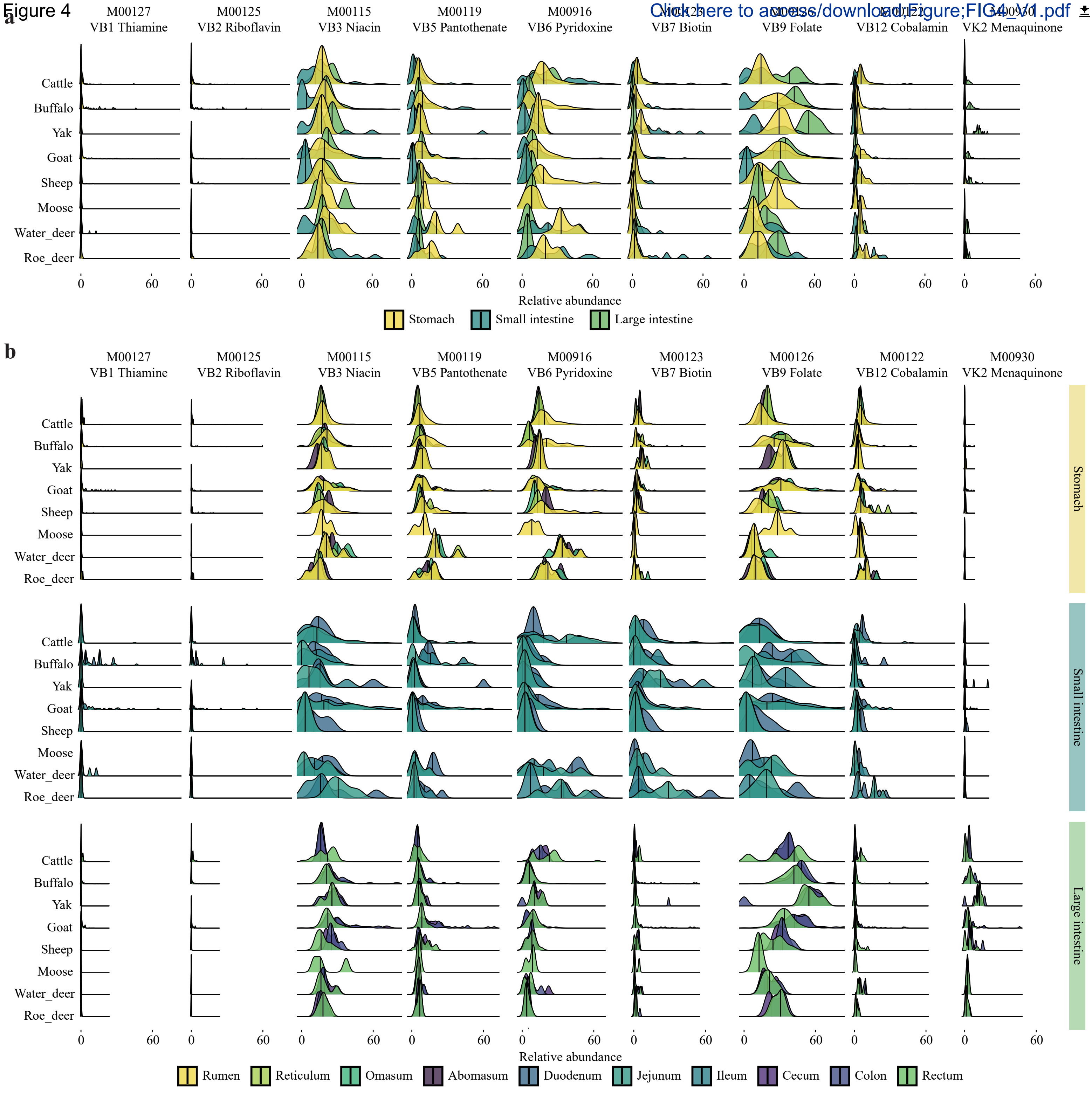

Figure 5

Click here to access/download;Figure;FIG5_V1.pdf

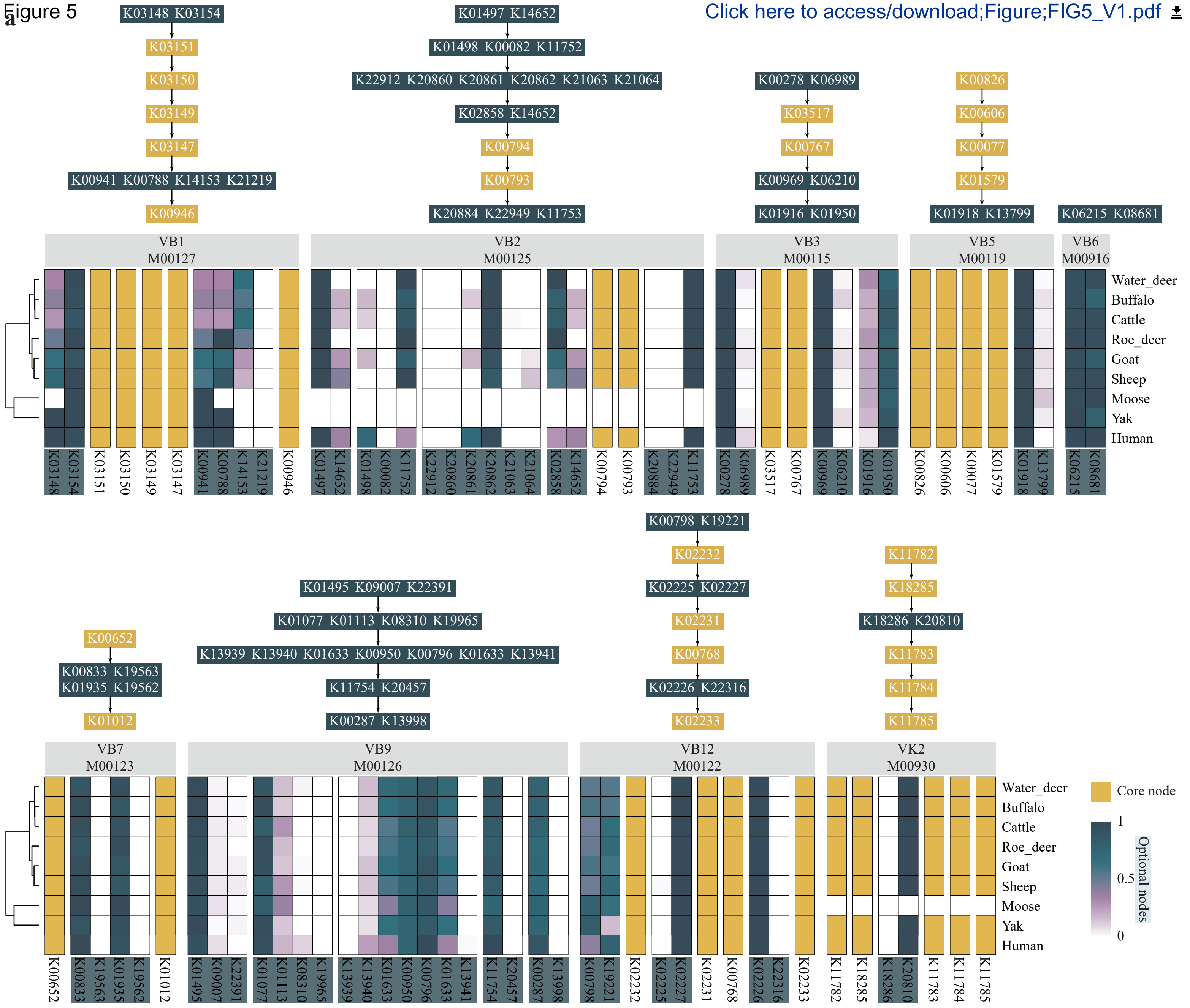

Figure 6

Click here to access/download;Figure;FIG6_V1.pdf

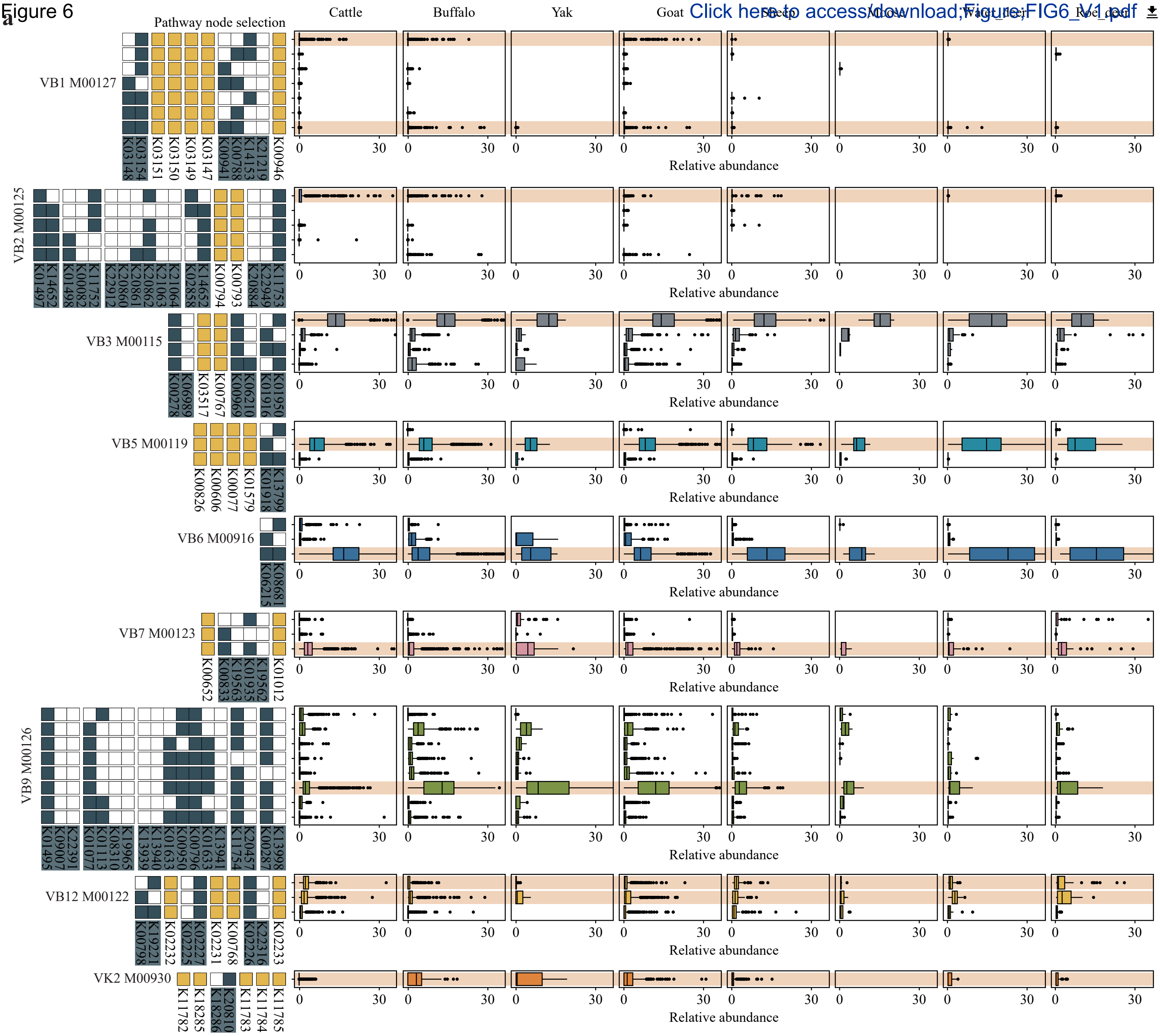

Click here to access/download
Supplementary Material
Point-by-point response - ver 1.0 -FENG.docx

Supplementary figure

Click here to access/download
Supplementary Material
Feng_etal_Ruminant_CRMC_vitamin_Supplement_Figure Ver 1.1.pdf

Supplementary table

Click here to access/download
Supplementary Material
renamed_72393.xlsx

Cover letter
Click here to access/download;Personal Cover;Feng_etal_Ruminant_CRMC_vitamin_cover_letter_Ver_

Dear Editor,

We would like to submit our manuscript "A comprehensive ruminant microbial catalog (CRMC) reveals convergent selection for key vitamin-synthesizing pathways and genes across ruminants and human" for publication as a Research Article in *GigaScience.*

The unique gastrointestinal tract structure of ruminants serves as a natural microbial reservoir, playing a vital role in digestion, absorption, and nutritional balance. Although previous studies have begun to reveal functional features of the ruminant gastrointestinal tract microbiome, no large-scale reference genome catalog has yet been established to systematically characterize the core microbial taxa and functional traits of the ruminant gastrointestinal tract microbiome[1-3].

Here, building upon our previous research[4-6], we analyzed 2,325 metagenomic samples collected from 10 gastrointestinal tract regions across 8 ruminant hosts to comprehensively reconstruct and explore the structure of the ruminant gastrointestinal tract microbiome and the distribution patterns of vitamin-synthesizing microbes. Our main findings include:

- We reconstructed a comprehensive ruminant gastrointestinal microbiome reference genome catalog (CRMC) comprising 39,696 MAGs, including 981 archaeal and 38,715 bacterial genomes, and established eight host-specific catalogs under a unified analytical framework.
- At the species level (ANI 95%), the CRMC nearly encompassed the existing ruminant genome collection (RGMGC) and achieved the highest mapping rate (~83.45%) across 2,325 metagenomic datasets, outperforming GTDB and other catalogs.
- We revealed eight core phyla shared among ruminants that underpin digestion and methanogenesis, while feeding practices shaped host-specific and convergent adaptations in polysaccharide metabolism.

- Furthermore, we identified 17,349 vitamin-synthesizing microbes showing unified pathway selection consistent with the human gut and mapped their spatial distribution across gastrointestinal regions.
- Importantly, we uncovered unified and conserved patterns of multi-gene node selection in vitamin biosynthesis, encompassing core-node preference and multi-gene co-selection, highlighting the functional stability and evolutionary conservation of ruminant vitamin-synthesizing mechanisms.

In summary, by reconstructing the ruminant gastrointestinal microbiome reference genome catalog (CRMC), we elucidated the core microbial taxa and functional traits across ruminants, together with the pathway preferences and spatial distribution of vitamin-synthesizing microbes. These findings provide a robust genomic framework for advancing ruminant microbiome research, offering insights into gene co-selection for microbial synthetic biology and guiding microbiome-based applications in ruminant systems. Our work will be of high interest to those in the fields of microbiome and ruminant researches and the broad readership of *GigaScience*.

We confirm that this manuscript has not been published elsewhere and is not under consideration by another journal.

All authors have approved the manuscript and agree with its submission to *GigaScience*.

Yours, Sincerely,

Wei-Hua Chen (on behalf of all authors)

weihuachen@hust.edu.cn

Huazhong University of Science and Technology

References:

1. Mi J, Jing X, Ma C, Shi F, Cao Z, Yang X, Yang Y, Kakade A, Wang W, Long R: **A metagenomic catalogue of the ruminant gut archaeome**. *Nature Communications* 2024, **15**(1):9609.
2. Xie F, Jin W, Si H, Yuan Y, Tao Y, Liu J, Wang X, Yang C, Li Q, Yan X: **An integrated gene catalog and over 10,000 metagenome-assembled genomes from the gastrointestinal microbiome of ruminants**. *Microbiome* 2021, **9**(1):137.
3. Jiang Q, Lin L, Xie F, Jin W, Zhu W, Wang M, Qiu Q, Li Z, Liu J, Mao S: **Metagenomic insights into the microbe-mediated B and K2 vitamin biosynthesis in the gastrointestinal microbiome of ruminants**. *Microbiome* 2022, **10**(1):109.
4. Tong F, Wang T, Gao NL, Liu Z, Cui K, Duan Y, Wu S, Luo Y, Li Z, Yang C: **The microbiome of the buffalo digestive tract**. *Nature Communications* 2022, **13**(1):823.
5. Cao Y, Feng T, Wu Y, Xu Y, Du L, Wang T, Luo Y, Wang Y, Li Z, Xuan Z: **The multi-kingdom microbiome of the goat gastrointestinal tract**. *Microbiome* 2023, **11**(1):219.
6. Wu Y, Gao N, Sun C, Feng T, Liu Q, Chen W-H: **A compendium of ruminant gastrointestinal phage genomes revealed a higher proportion of lytic phages than in any other environments**. *Microbiome* 2024, **12**(1):69.
