# Supplementary material for: A comprehensive ruminant microbial catalog (CRMC) reveals convergent selection for key vitamin-synthesizing pathways and genes across ruminants and human
Source: Gigascience. 2026 Feb 25;15:giag016. doi: 10.1093/gigascience/giag016 (PMC13108260; doi:10.1093/gigascience/giag016)
Supplement: giag016_Authors_Response_To_Reviewer_Comments_original_submission [file giag016_authors_response_to_reviewer_comments_original_submission.pdf]

Author's Response To Reviewer Comments

Dear Dr. Dongni Ma,
We would like to extend our sincere appreciation for your dedicated efforts throughout our manuscript entitled “A comprehensive ruminant microbial catalog (CRMC) reveals convergent selection for key vitamin-synthesizing pathways and genes across ruminants and human”. Your guidance and support have been instrumental in helping us refines our work. We have carefully addressed the reviewer's suggestions and believe our revisions now offer a clear and comprehensive presentation of our research.
Thank you for your valuable support in enhancing the quality of our work. To facilitate your review of our revisions, the following is a point-by-point response to the questions and comments.

Sincerely yours,
Wei-Hua Chen (on behalf of all authors)

Response to referee 1
NOTE: for convenience purposes, we broke the reviewers’ comments into relevant sections, numbered them, and provided point-to-point responses. We did not truncate nor rephrase the comments by any means.
General remark
In this manuscript, the authors constructed a comprehensive ruminant microbial reference catalog (CRMC) based on 2,325 metagenomic samples across diverse ruminant hosts. The study systematically compares shared and unique microbial taxa and functions among different ruminants and highlights an interesting convergence in vitamin-synthesizing functional characteristics between the ruminant and human gut microbiomes. Overall, the study holds certain significance and provides a valuable resource for the field. The figures and the main text are well-organized.
Response:
Thank you for your positive feedback for our manuscript. We greatly appreciate your thorough review and the specific comments you provided. Your thoughtful feedback and suggestions have significantly contributed to the refinement and quality enhancement of this manuscript. We have carefully reviewed the attached document and will now address each comment in detail.

Comments for the Author
Comment #1
This study is based on second-generation metagenomics, the discussion should discuss the potential enhancements in MAG completeness and functional gene retrieval offered by third-generation sequencing in future studies.
Response:
Thank you for your suggestion. We have revised the manuscript to acknowledge the limitations of using only second-generation sequencing and to highlight the potential of third-generation sequencing for improving data quality in future work. The revised text now reads: "Although this study did not involve third-generation sequencing data, resulting in fewer MAGs reaching complete status, the CRMC not only nearly … for future studies. In future studies, the incorporation of third-generation sequencing data will further enhance the CRMC, yielding more complete-level MAGs." (Lines 588 - 596).
Action:
Corrected. You can find the revised text at line 588 - 596.

Comment #2
Although Archaea constitute only 2.47% of the MAGs, they are critical for rumen function (e.g., methanogenesis). Need to list the major archaeal families and genera annotated in the CRMC.
Response:
Thank you for your suggestion. We have updated the Results section to include the taxonomic composition of the archaeal MAGs. The revised text now reads: "The archaeal MAGs mainly belong to three phyla: Halobacteriota (predominantly family Methanocorpusculaceae and genus Methanocorpusculum), Methanobacteriota (predominantly family Methanobacteriaceae and genus Methanobrevibacter), and

Thermoplasmatota (predominantly family Methanomethylophilaceae and genus UBA71)." (Lines 272 - 276).
Action:
Corrected. You can find the revised text at line 272 - 276.

Comment #3
Regarding the identification of vitamin-synthesizing microbes, the criteria need clarification. Did it require complete pathways on a single contig or within a MAG?
Response:
Thank you for your suggestion. We have clarified the identification criteria in the Methods section to specify that the presence of all pathway genes within a single MAG is enough. The revised text now reads: "1. Only MAGs encoding the full complement of genes for an entire vitamin biosynthesis pathway were considered as vitamin-synthesizing candidates. Specifically, the identification criteria required the presence of all pathway genes within a single MAG, but not necessarily on a single contig." (Lines 196 - 198).
Action:
Corrected. You can find the revised text at line 196 - 198.

Comment #4
The discussion needs to speculate on the underlying reasons for the observed functional convergence between ruminant and human gut microbiomes.
Response:
Thank you for your suggestion. We have added a detailed explanation in the Discussion section regarding the consistent vitamin synthesis pathways observed in both ruminant and human guts. The revised text now reads: "We speculate that this functional convergence is driven by shared environmental selective pressures within the gut, such as stable temperature, anaerobic conditions, and consistent physicochemical properties. Consequently, the vitamin-synthesizing bacteria that stably colonize these gut environments hold significant potential for identification and application as next-generation probiotics." (Lines 632 - 636).
Action:
Corrected. You can find the revised text at line 632 - 636.

Comment #5
To ensure project reproducibility, the open-source link to the workflow needs to be added in the methods section.
Response:
Thank you for your suggestion. We have added the open-source link to our workflow in the methods section. The revised text now reads: "The software commands and scripts used in this study are available on GitHub (https://github.com/fengtong-bio/CRMC)." (Lines 244 - 245).
Action:
Corrected. You can find the revised text at line 244 - 245.

Response to referee 2
NOTE: for convenience purposes, we broke the reviewers' comments into relevant sections, numbered them, and provided point-to-point responses. We did not truncate nor rephrase the comments by any means.
General remark
The unique gastrointestinal tract structure of ruminants serves as a natural microbial reservoir, playing a vital role in digestion, absorption, and nutritional balance. In this study, the authors constructed a comprehensive ruminant microbial reference catalog (CRMC) using 2,325 metagenomic samples and summarized the functional characteristics of vitamin-synthesizing microbes in ruminants. And a meaningful ruminant gastrointestinal tract microbiome reference genome catalog (CRMC) was constructed, which included 39,696 l MAGs. A total of 17,349 vitamin-synthesizing microbes spanning 9 biosynthetic pathways (thiamine, riboflavin, niacin, pantothenate, pyridoxine, biotin, folate, cobalamin, and menaquinone) were identified. This paper will be of interest to scientists who study the rumen function of herbivorous animals, including cattle and sheep, and is particularly meaningful for research on precision nutrition in the rumen. Overall, this study holds certain significance as a valuable resource for the ruminant microbial field. I agree to be published after minor revision.

Response:
Thank you for your positive assessment and for the opportunity to revise our manuscript. We greatly appreciate your detailed review and the constructive spirit of your comments. Your suggestions have been exceptionally helpful in guiding us to strengthen the arguments presented in our paper and to improve the presentation of our data. We have treated your feedback with the utmost seriousness and have conducted a comprehensive revision of the manuscript. In the following section, we provide a detailed account of how we have addressed each of your specific comments and incorporated your suggestions into the final version.

Comments for the Author
Comment #1
The terms "gastrointestinal tract" and "GIT" are used interchangeably; "GIT" needs to be used consistently throughout the text after the first definition.
Response:
Thank you for your suggestion. We have checked the full text and ensured that "GIT" is used consistently throughout the manuscript after its first mention.

Comment #2
In Figure 1f, the red portion needs to be explicitly labeled as "CRMC Unique" in the legend to prevent ambiguity.
Response:
Thank you for your suggestion. We have updated the legend in Figure 1f from "Unique" to "CRMC Unique" to ensure clarity and accuracy.

Comment #3
In Figure 3a, the figure legend needs to confirm that the color coding for phyla is consistent between the human and ruminant datasets.
Response:
Thank you for your suggestion. We have updated the legend for Figure 3a to explicitly confirm that the color scheme is consistent across datasets. The revised legend now reads: " Each color represents a different phylum, and bar length indicates the number of MAGs. The color coding for phyla is consistent between the ruminant and human datasets." (Lines 460 - 461).
Action:
Corrected. You can find the revised text at line 460 - 461.

Comment #4
The Methods section needs to clarify whether the screening criteria for identifying human gut vitamin-synthesizing microbes were consistent with the standards used for the CRMC.
Response:
Thank you for your suggestion. We have clarified in the Methods section that the human dataset was processed using the exact same criteria to ensure valid comparisons. The revised legend now reads: " Candidate vitamin-synthesizing MAGs were defined according to the following criteria (the same screening workflow was applied to the human IMGG dataset):" (Lines 193 - 194).
Action:
Corrected. You can find the revised text at line 193 - 194.

Comment #5
Open-source links for the software and code used in the Methods section need to be provided.
Response:
Thank you for your suggestion. We have added the open-source link to our workflow in the methods section. The revised text now reads: "The software commands and scripts used in this study are available on GitHub (https://github.com/fengtong-bio/CRMC)." (Lines 244 - 245).
Action:
Corrected. You can find the revised text at line 244 - 245.

Cookie Preference Center

We use cookies which are necessary to make our site work. We may also use additional cookies to analyze, improve and personalize our content and your digital experience. For more information, see our Cookie Policy.

You may choose not to allow some types of cookies. However, blocking some types may impact your experience of our site and the services we are able to offer. See the different category headings below to find out more or change your settings.

Allow all

Manage Consent Preferences

Strictly Necessary Cookies

Always active

These cookies are necessary for the website to function and cannot be switched off in our systems. They are usually only set in response to actions made by you which amount to a request for services, such as setting your privacy preferences, logging in or filling in forms. You can set your browser to block or alert you about these cookies, but some parts of the site will not then work.

Functional Cookies

These cookies enable the website to provide enhanced functionality and personalisation. They may be set by us or by third party providers whose services we have added to our pages. If you do not allow these cookies then some or all of these services may not function properly.

Performance Cookies

These cookies allow us to count visits and traffic sources so we can measure and improve the performance of our site. They help us to know which pages are the most and least popular and see how visitors move around the site.

Targeting Cookies

These cookies may be set through our site by our advertising partners. They may be used by those companies to build a profile of your interests and show you relevant adverts on other sites. If you do not allow these cookies, you will experience less targeted advertising.

Cookie List

Clear

Apply Cancel
Consent Leg.Interest

Confirm my choices

Power
