# Supplementary material for: A comprehensive ruminant microbial catalog (CRMC) reveals convergent selection for key vitamin-synthesizing pathways and genes across ruminants and human
Source: Gigascience. 2026 Feb 25;15:giag016. doi: 10.1093/gigascience/giag016 (PMC13108260; doi:10.1093/gigascience/giag016)
Supplement: giag016_Reviewer_2_Report_original_submission — Reviewer 2 -- 1/15/2026 [file giag016_reviewer_2_report_original_submission.pdf]

Reviewer Report

Title: A comprehensive ruminant microbial catalog (CRMC) reveals convergent selection for key vitamin-synthesizing pathways and genes across ruminants and human

Version: Original Submission **Date:** 1/15/2026

Reviewer name: Reviewer 2

Reviewer Comments to Author:

The unique gastrointestinal tract structure of ruminants serves as a natural microbial reservoir, playing a vital role in digestion, absorption, and nutritional balance. In this study, the authors constructed a comprehensive ruminant microbial reference catalog (CRMC) using 2,325 metagenomic samples and summarized the functional characteristics of vitamin-synthesizing microbes in ruminants. And a meaningful ruminant gastrointestinal tract microbiome reference genome catalog (CRMC) was constructed, which included 39,696 l MAGs. A total of 17,349 vitamin-synthesizing microbes spanning 9 biosynthetic pathways (thiamine, riboflavin, niacin, pantothenate, pyridoxine, biotin, folate, cobalamin, and menaquinone) were identified. This paper will be of interest to scientists who study the rumen function of herbivorous animals, including cattle and sheep, and is particularly meaningful for research on precision nutrition in the rumen. Overall, this study holds certain significance as a valuable resource for the ruminant microbial field. I agree to be published after minor revision.
Minor revision comments:
1. The terms "gastrointestinal tract" and "GIT" are used interchangeably; "GIT" needs to be used consistently throughout the text after the first definition.
2. In Figure 1f, the red portion needs to be explicitly labeled as "CRMC Unique" in the legend to prevent ambiguity.
3. In Figure 3a, the figure legend needs to confirm that the color coding for phyla is consistent between the human and ruminant datasets.
4. The Methods section needs to clarify whether the screening criteria for identifying human gut vitamin-synthesizing microbes were consistent with the standards used for the CRMC.
5. Open-source links for the software and code used in the Methods section need to be provided.

Methods

Are the methods appropriate to the aims of the study, are they well described, and are necessary controls included? Choose an item.

Conclusions

Are the conclusions adequately supported by the data shown? Choose an item.

Reporting Standards

Does the manuscript adhere to the journal's guidelines on minimum standards of reporting? Choose an item.

Choose an item.

Statistics

Are you able to assess all statistics in the manuscript, including the appropriateness of statistical tests used? Choose an item.

Quality of Written English

Please indicate the quality of language in the manuscript: Choose an item.

Declaration of Competing Interests

Please complete a declaration of competing interests, considering the following questions:

- Have you in the past five years received reimbursements, fees, funding, or salary from an organisation that may in any way gain or lose financially from the publication of this manuscript, either now or in the future?
- Do you hold any stocks or shares in an organisation that may in any way gain or lose financially from the publication of this manuscript, either now or in the future?
- Do you hold or are you currently applying for any patents relating to the content of the manuscript?
- Have you received reimbursements, fees, funding, or salary from an organization that holds or has applied for patents relating to the content of the manuscript?
- Do you have any other financial competing interests?
- Do you have any non-financial competing interests in relation to this paper?

If you can answer no to all of the above, write 'I declare that I have no competing interests' below. If your reply is yes to any, please give details below.

I declare that I have no competing interests.

I agree to the open peer review policy of the journal. I understand that my name will be included on my report to the authors and, if the manuscript is accepted for publication, my named report including any attachments I upload will be posted on the website along with the authors' responses. I agree for my report to be made available under an Open Access Creative Commons CC-BY license (http://creativecommons.org/licenses/by/4.0/). I understand that any comments which I do not wish to be included in my named report can be included as confidential comments to the editors, which will not be published.

Choose an item.

To further support our reviewers, we have joined with Publons, where you can gain additional credit to further highlight your hard work (see: https://publons.com/journal/530/gigascience). On publication of this paper, your review will be automatically added to Publons, you can then choose whether or not to claim your Publons credit. I understand this statement.

Yes Choose an item.
